# Loss of the endoplasmic reticulum protein canopy 1 disrupts the function and circuit organization of V2R-expressing vomeronasal sensory neurons

**DOI:** 10.1242/dev.205386

**Published:** 2026-06-22

**Authors:** Nicholas A. Mathias, Nikki M. Dolphin, Noah M. LeFever, Enrico Amato, Carlsy S. Ybanez, Anthony Ruquet, Paolo E. Forni

**Affiliations:** Department of Biological Sciences, The Center for Neuroscience Research, The RNA Institute, University at Albany, State University of New York, Albany, NY 12222, USA

**Keywords:** Canopy 1 (Cnpy1), Vomeronasal neurons, Endoplasmic reticulum, ER stress, V2R receptors, Tfap2e (Ap-2ε)

## Abstract

The vomeronasal organ (VNO) is a specialized chemosensory structure that detects chemical cues involved in predator avoidance, social interactions and reproductive behaviors. In mice, the VNO contains distinct vomeronasal sensory neurons (VSNs) that express vomeronasal receptors (VRs) and formyl peptide receptors. Apical VSNs express Meis2, V1Rs and Gαi2, whereas basal VSNs express Tfap2e (also known as AP-2ε), V2Rs and Gαo. Type 2 VRs (V2Rs) are classified into families A-E, and basal neurons co-express a family C receptor with a VR from another family. Single-cell RNA-sequencing identified ∼980 genes differentially expressed between V1R- and V2R-expressing neurons, many of which are linked to endoplasmic reticulum (ER) functions. Notably, canopy 1 (Cnpy1) is highly enriched in V2R-expressing neurons. The VNO of *Cnpy1* knockout mice develops normally but undergoes progressive loss of V2R-expressing neurons, which is associated with increased ER stress gene expression, upregulation of family C V2R mRNAs and reduced V2R protein levels. V2R-expressing VSNs in Cnpy1 knockouts fail to respond to pheromones, show altered guidance and adhesion gene expression, and display disrupted connectivity with the accessory olfactory bulb. These findings emphasize the role of cell-specific ER protein repertoires in maintaining neuronal function.

## Teaser

**Summary:** Cnpy1 is an ER protein essential for proteostasis in V2R-expressing vomeronasal neurons. Its loss reduces V2R expression and localization to the lumen, impairing neuronal function and connectivity, and leading to progressive degeneration of basal VSNs.

## INTRODUCTION

Different cell types are defined by the specific genes and proteins they express. However, how specialized differences in the endoplasmic reticulum support the processing of cell-specific proteomes remains poorly investigated in development, aging and neurodegeneration.

The sensory epithelium of the rodent vomeronasal organ (VNO) is primarily populated by vomeronasal sensory neurons (VSNs), which express either V1R or V2R vomeronasal receptor gene families (Francia et al., 2014; Herrada and Dulac, 1997; Matsunami and Buck, 1997; Ryba and Tirindelli, 1997; Silvotti et al., 2007) or formyl peptide receptors (FPRs) (Ackels et al., 2014; Dietschi et al., 2013; Liberles et al., 2009; Rivière et al., 2009). Both V1R- and V2R-expressing VSNs arise from same pool of progenitors primarily located in the marginal zones postnatally. The VNO, like the main olfactory epithelium, exhibits postnatal neurogenesis throughout life (Brann and Firestein, 2010). After differentiation, V1R-expressing VSNs localize apically toward the lumen, while V2R-expressing VSNs localize toward the basal lamina. The V1R/V2R dichotomy is determined by differential Notch signaling in newly formed VSNs (Katreddi et al., 2022), after which these neuronal types begin to diverge in their expression of transcription factors, G-protein subunits, adhesion molecules, specific VR genes and guidance receptors (Hills et al., 2024; Katreddi et al., 2022; Lin et al., 2022). V1R- and V2R-expressing VSNs project and synapse onto projection neurons of the anterior and posterior accessory olfactory bulb (AOB), respectively.

We have previously reported that the gene encoding the transcription factor AP-2ε (*Tfap2e*), which is among the most differentially expressed genes between V1R^+^ and V2R^+^ VSNs, is essential to sustain the basal (V2R^+^) VSN program after it is initiated by Notch signaling and Bcl11b expression (Enomoto et al., 2011; Katreddi et al., 2022; Lin et al., 2022). In the VNO, AP-2ε upregulates the expression of about one-third of the basal (V2R) neuronal type-specific genes, while repressing approximately one-third of the apical (V1R) genes. Ectopic expression of AP-2ε in the apically located V1R-expressing VSNs was found to enhance the mRNA expression of the basally enriched endoplasmic reticulum (ER) genes, including canopy 1 (*Cnpy1*), and the ER protein calreticulin 4, which has been proposed to play a key role in V2R receptor post-translational processing (Dey and Matsunami, 2011; Lin et al., 2022). Repressor activity for AP-2ε has been observed for the V1R-expressing apical enriched ER protein calreticulin (Lin et al., 2022).

The V1R and V2R families are 7-transmembrane G-protein-coupled receptors that differ in their levels of molecular complexity and evolution (Matsunami and Buck, 1997; Mombaerts, 2004; Yang et al., 2005; Young and Trask, 2007). V1R- and V2R-expressing VSNs show significant differences in ER structure and expression of ER protein repertoires (Devakinandan et al., 2024). These findings suggest that the two major VSN neuronal types rely on specialized ER protein compositions to sustain proteostasis and ER stress arising from molecular differences between V1R and V2Rs, and thus their processing and abundance. Consequently, if each neuronal subtype relies on its own unique ER protein repertoire, this could lead to different genetic vulnerabilities across chemosensory neuronal populations when specific ER proteins are disrupted.

Cnpy1 is the founder of the canopy family (Li et al., 2025). The canopy proteins are part of the saposin-like superfamily and all share a common saposin fold, which is believed to facilitate their interaction with plasma membranes and enable dimerization (Schildknegt et al., 2019). Previous *in vivo* studies in zebrafish pointed to roles for Cnpy1 in establishing the mid-hindbrain barrier with a proposed role in Fgf8 signaling through Fgfr1 (Hirate and Okamoto, 2006; Matsui et al., 2011). In this study, we examined Cnpy1 expression in the VNO of mice and the impact of Cnpy1 loss of function. Our research shows that Cnpy1 is a key component in the gene regulatory networks responsible for function and homeostasis of V2R-expressing VSNs. Effects of Cnpy1 loss of function on family C V2Rs suggest differences in regulation of gene expression among V2Rs belonging to different families.

## RESULTS

### V1R- and V2R-expressing VSNs have different ER molecular compositions

**Fig. 1.**
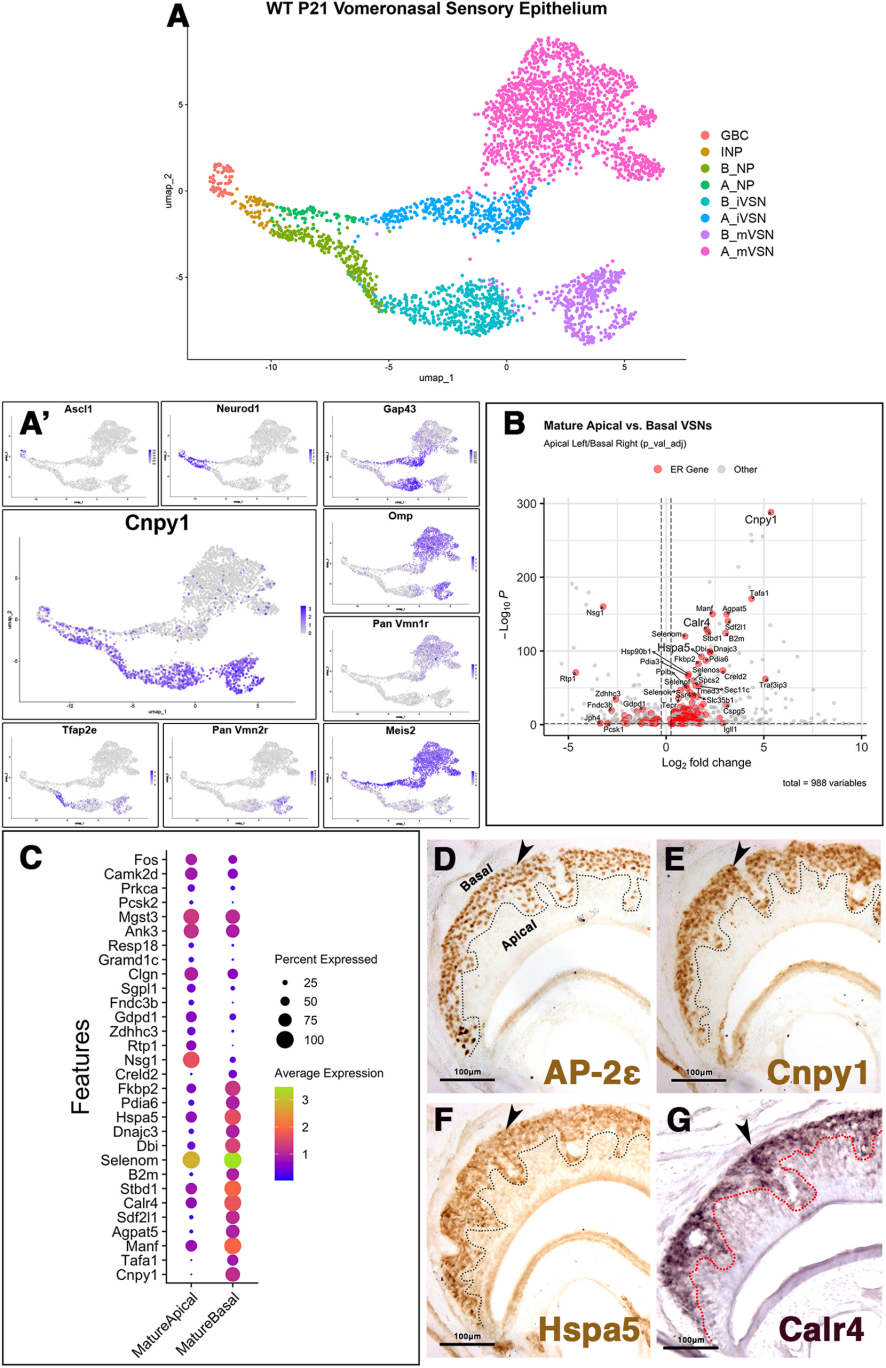
**Transcriptomic and histological characterization of apical and basal VSNs.** (A) UMAP of wild-type P21 VNO single-cell RNA-seq showing progression from *Ascl1*^+^ GBCs and *Neurod1*^+^ INPs to *Gap43*^+^ immature and mature apical (V1R) and basal (V2R) VSNs. (A′) Feature plots for key markers, including *Ascl1*, *Neurod1*, *Gap43*, *Omp*, *Tfap2e* (AP-2ε), pan-*Vmn1r*, pan-*Vmn2r*, *Meis2* and the ER gene *Cnpy1*. Note the enrichment of *Cnpy1* along the basal VSNs from immature to mature stages. (B) Volcano plot of mature apical versus basal VSNs highlighting ER-enriched genes (*Cnpy1*, *Hspa5* and *Calr4*). (C) Dot plot of the top ER-related differentially expressed genes based on GO analysis. (D-F) Immunohistochemistry for AP-2ε, Cnpy1 and HSPA5 (BiP) confirms basal enrichment (arrowheads). (G) *In situ* hybridization for *Calr4* shows strong basal expression (arrowhead). Scale bars: 100 μm.

Single-cell suspensions were obtained from VNOs of wild-type P21 mice, then sequenced and processed. The resulting single-cell transcriptomic data were filtered to isolate the VNO sensory epithelium based on expression of known markers (Fig. 1, Fig. S1), and a UMAP illustrates the main stages of VSN differentiation. This analysis resolved Ascl1^+^ globose basal progenitor cells (GBCs) and their transition into Neurog1^+^ and NeuroD1^+^ immediate neuronal precursors (iNPs), followed by more advanced neuronal precursors in which Neurod1 expression declines as GAP43 rises in immature VSNs (Fig. 1A). These will ultimately mature into OMP^+^ VSNs along either the Meis2/V1R or AP-2ε/V2R trajectories. Together, these data recapitulate the expected developmental continuum from progenitor to fully differentiated V1R- or V2R-expressing sensory neurons endowed with mature sensory and synaptic competence. We performed differential gene expression analysis between mature basal (V2R) and apical (V1R) VSNs. In line with a recent study (Devakinandan et al., 2024), we observed a significant representation of genes encoding proteins belonging to the endoplasmic reticulum or that control ER biosynthesis and functions according to the ‘endoplasmic reticulum’ Gene Ontology (GO) term (GO:0005783). In mature V1R-expressing VSNs, 31 of the 279 genes (11.1%) that are enriched were classified as ER-related genes. In mature V2R-expressing VSNs, 145 of the 709 genes (20.5%) enriched were identified as ER-related proteins. To visualize this diversity in ER protein expression across mature cell types, we created a dot plot (Fig. 1C) showing the top 15 ER-related genes for apical and basal VSNs. Among the various enriched genes in the V2R-expressing VSNs, we noticed strong expression of *Hspa5*, *Cnpy1* and calreticulin 4 (*Calr4*) (Fig. 1A-C). Immunolabeling and *in situ* hybridization confirmed enrichments of these ER genes in the basal VSNs (Fig. 1D-G).

### Cnpy1 expression is selectively enriched in mature V2R-expressing VSNs

**Fig. 2.**
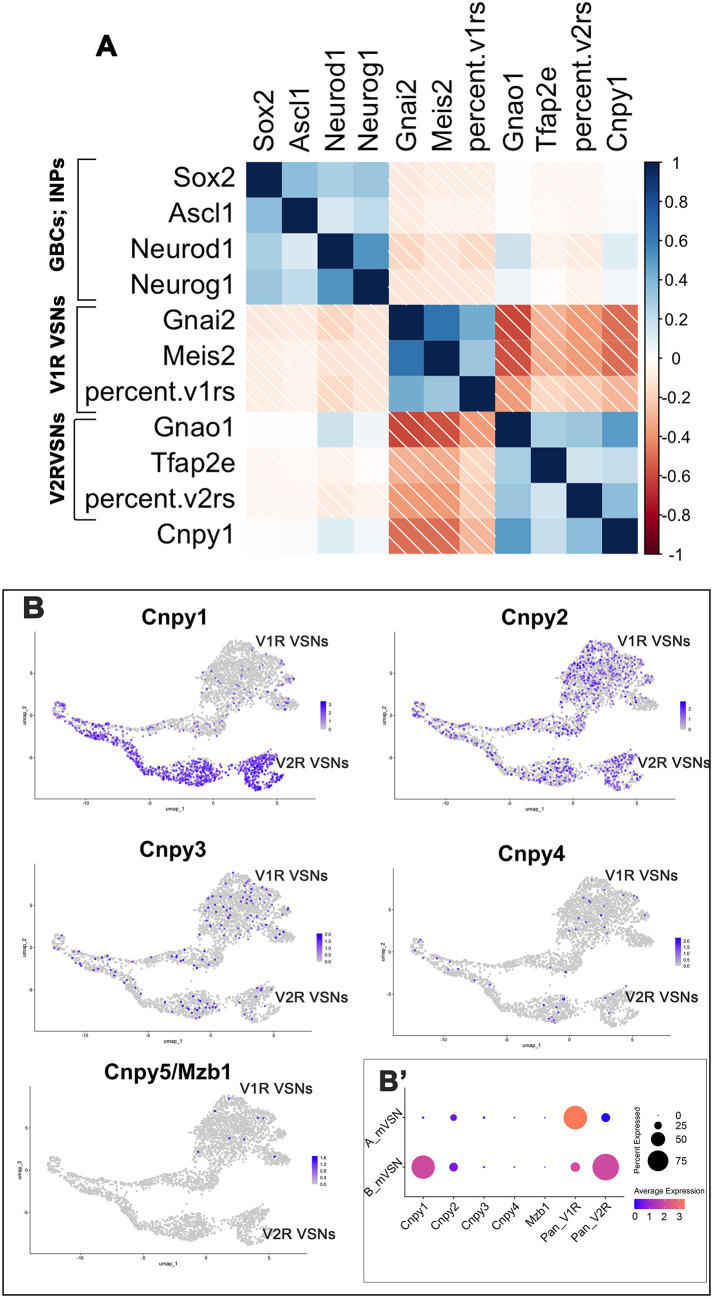
**Cnpy1 expression correlates with the maturation program of basal V2R-expressing VSNs.** (A) Correlation matrix showing that Cnpy1 expression positively correlates with markers of differentiating and maturing basal VSNs, including Gnao1 (Gαo) and *Tfap2e* (AP-2ε), and the proportion of V2R-expressing neurons, and is inversely correlated with apical V1R markers. Blue coloring indicates positive correlation; red coloring with diagonal lines indicates negative correlation. (B) Feature plots of Cnpy1, Cnpy2, Cnpy3, Cnpy4 and Cnpy5 (Mzb1) across the VSNs. UMAP shows that only Cnpy1 is selectively enriched in V2R-expressing VSNs. (B′) Dot plot summarizing relative expression levels and the percentage of cells positive for CNPY family members in mature apical and basal VSNs, highlighting the selective basal enrichment of Cnpy1.

Feature plot analyses of VNO sc-RNAseq revealed that Cnpy1 mRNA is first detected in proliferative globose basal cells (Ascl1^+^) and early neuronal progenitors/precursors (Neurog1^+^ and Neurod1^+^) (Fig. 1A; Fig. 2B). As differentiation progresses, Cnpy1 mRNA expression is rapidly downregulated in the V1R lineage (Meis2^+^; Gnαi2^+^), while its expression remains sustained in the lineage (Fig. 1A; Fig. 2A). Correlation analyses confirmed a strong association between Cnpy1 mRNA expression and markers of basal VSN maturation, including Tfap2e (AP-2ε), Gαo (Gnao1) and V2R genes (Fig. 2A).

Other members of the Cnpy family (Schildknegt et al., 2019), including Cnpy2, Cnpy3, Cnpy4 and Cnpy5 (also known as Mzb1), showed distinct expression profiles in the VNO. Cnpy2 was broadly expressed across both V1R and V2R lineages, whereas Cnpy3, Cnpy4 and Cnpy5/Mzb1 were detected only in sparse subsets of VSNs (Fig. 2B). Together, these data identify Cnpy1 as the only Cnpy family member selectively associated with the V2R lineage.

### In juvenile animals, loss of Cnpy1 did not affect gross VNO morphology

**Fig. 3.**
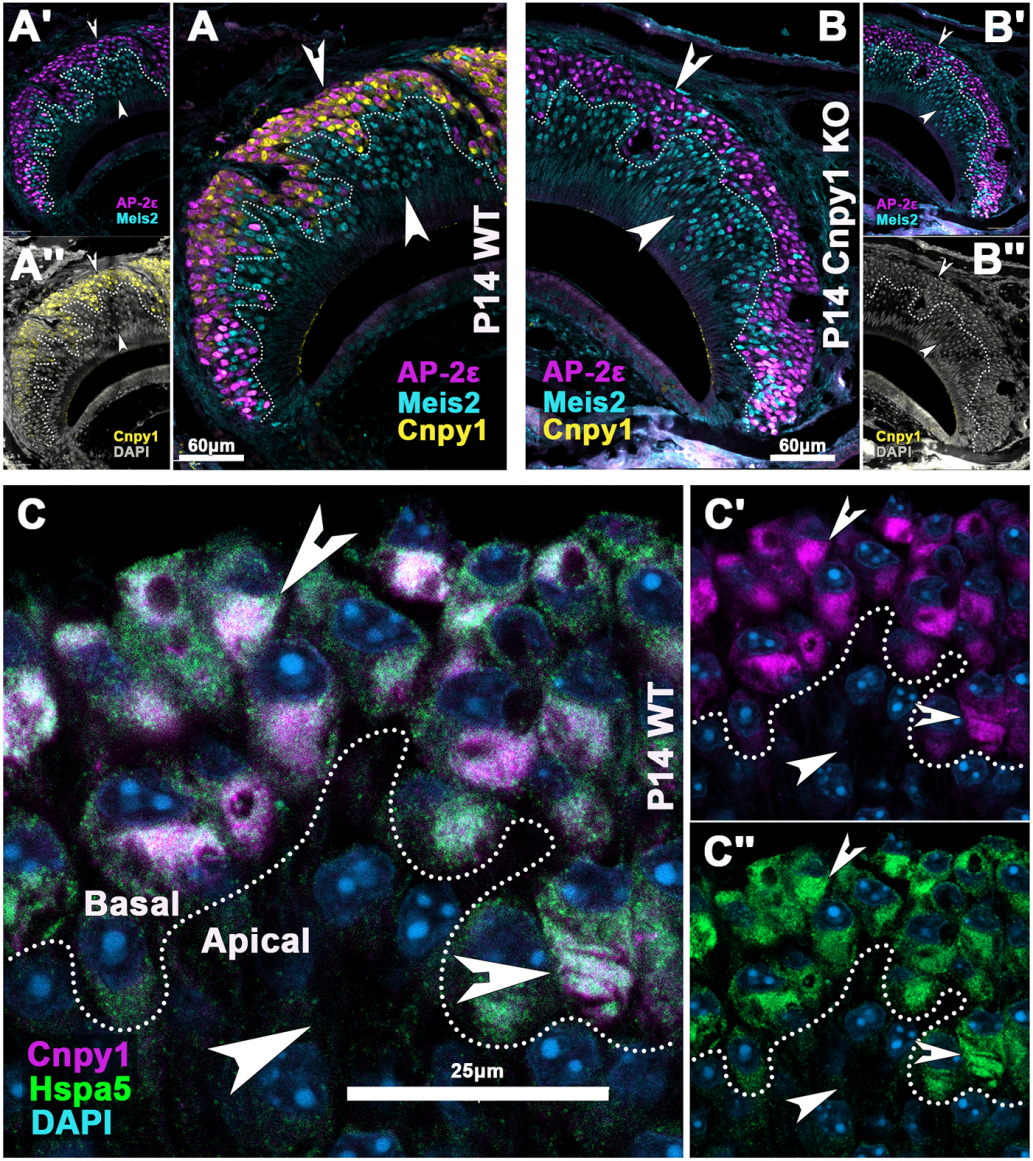
**CNPY1 protein is enriched in basal VSNs and colocalizes with the ER chaperone HSPA5.** (A,A′) Immunofluorescence for AP-2ε, Meis2 and Cnpy1 in P14 wild-type VNO shows robust Cnpy1 expression in basal V2R-expressing VSNs (notched arrowheads). (B-B″) P14 *Cnpy1* KO VNOs show AP-2ε immunoreactivity and normal apical-basal layering but complete loss (notched arrowheads) of CNPY1 immunoreactivity (arrowheads). (C-C″) High-magnification images showing that CNPY1 and the ER chaperone HSPA5 (BiP) colocalize in basal VSNs (notched arrowheads), whereas neither protein is detectable in apical VSNs (arrowheads). Scale bars: 60 μm in A and B; 25 μm in C.

To assess Cnpy1 function, we analyzed a commercially available *Cnpy1* knockout (KO) mouse line (C57BL/6J-Cnpy1em1cyagen) carrying a 6.2 kb deletion of the coding sequence. *Cnpy1* KO mice were viable and fertile, and heterozygous animals were intercrossed to generate *Cnpy1*^−/−^ mutants and wild-type controls. Immunolabeling at 2 weeks after birth confirmed that Cnpy1 protein was restricted to AP-2ε^+^ basal VSNs in wild-type animals, while *Cnpy1*^−/−^ littermates showed complete loss of Cnpy1 immunoreactivity but preserved AP-2ε expression (Fig. 3B).

Analysis of the postnatal vomeronasal epithelium showed that both major VSN subtypes, highlighted by Meis2 and AP-2ε expression, were present in *Cnpy1* KOs, with normal segregation and overall morphology comparable to wild-type controls (Fig. 3A-B″).

Notably, unlike the Cnpy1 orthologue of zebrafish and other Cnpy family members, mammalian Cnpy1 lacks a canonical ER localization signal (Schildknegt et al., 2019). Nonetheless, Cnpy1 expression overlapped with the ER chaperone Hspa5, supporting its localization to the endoplasmic reticulum in V2R-expressing VSNs (Fig. 3C). Cnpy1 transcripts were also found in the developing hindbrain, GBCs and olfactory sensory neuron precursors, but protein was detectable only in V2R-expressing VSNs (Fig. 3; Fig. S2).

### Cnpy1 KO mice exhibit a progressive loss of V2R-expressing VSNs

**Fig. 4.**
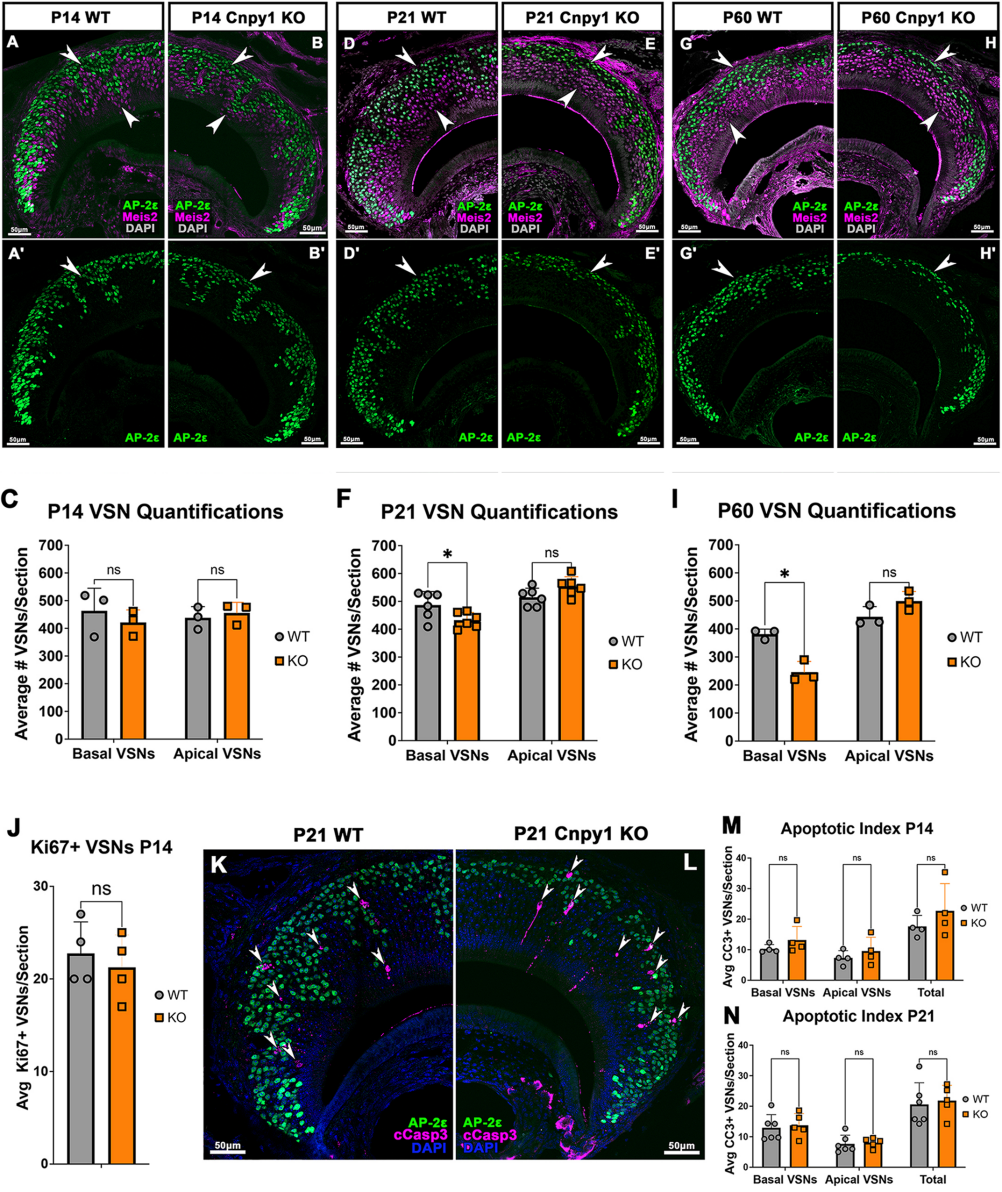
**Loss of Cnpy1 leads to progressive loss of basal VSNs.** (A-B′) P14 wild-type and *Cnpy1* KO VNO sections stained for AP-2ε (green, notched arrowheads), Meis2 (magenta, arrowheads) and DAPI show no obvious differences. (C) Quantification confirms no significant changes in AP-2ε-expressing or Meis2-expressing VSNs at P14. (D-F) At P21, *Cnpy1* KO shows reduced AP-2ε^+^ basal VSNs, while apical VSNs remain unchanged. (G-I) Basal VSN loss is more pronounced at P60. In D-E′,G-H′, notched arrowheads indicate AP-2ε-expressing cells and arrowheads indicate Meis2-expressing cells. (J) Ki67^+^ proliferation is comparable at P14. (K-N) cCasp3 staining (notched arrowheads) and apoptotic index analyses at P14 and P21 show no significant differences. Data are mean±s.e.m. (*n*≥3). *P<0.05; ns, not significant (unpaired *t*-test with Welch's correction). Scale bars: 50 μm.

We immunostained controls and Cnpy1 KOs for transcription factors AP-2ε and Meis2 to quantify V1R- and V2R-expressing VSNs at various developmental stages (Fig. 4A-I). Two weeks after birth, wild-type and Cnpy1 KO mice showed similar numbers of apical Meis2-expressing and basal AP-2ε-expressing VSNs (Fig. 4C). Additionally, there were no significant differences in the number of proliferative cells (mKi67^+^) (Fig. 4J). However, at P21, the KO animals showed a significant 11% decrease in the number of basal AP-2ε-expressing neurons (Fig. 4D-E′,F). Examination of KO VNOs at P60 (Fig. 4G-H′,I) revealed a 42% reduction in number of the basal VSNs compared to wild-type controls.

To determine whether the dramatic reduction between P14 and P60 could be associated with increased apoptosis, we performed immunohistochemistry and quantification of cleaved caspase 3 (cCasp3) at P14 and P21 (Fig. 4K-N). At these stages, neither the total number of cCasp3^+^ cells nor the number of basal (Ap-2ε^+^ and cCasp3^+^) VSNs differed across genotypes (Fig. 4M,N). This finding is consistent with previous reports on Gγ8 KO (Montani et al., 2013), where progressive loss of V2R-expressing VSNs was not accompanied by a significant increase in apoptotic markers.

### Transcriptomic analysis suggests a degenerative phenotype

**Fig. 5.**
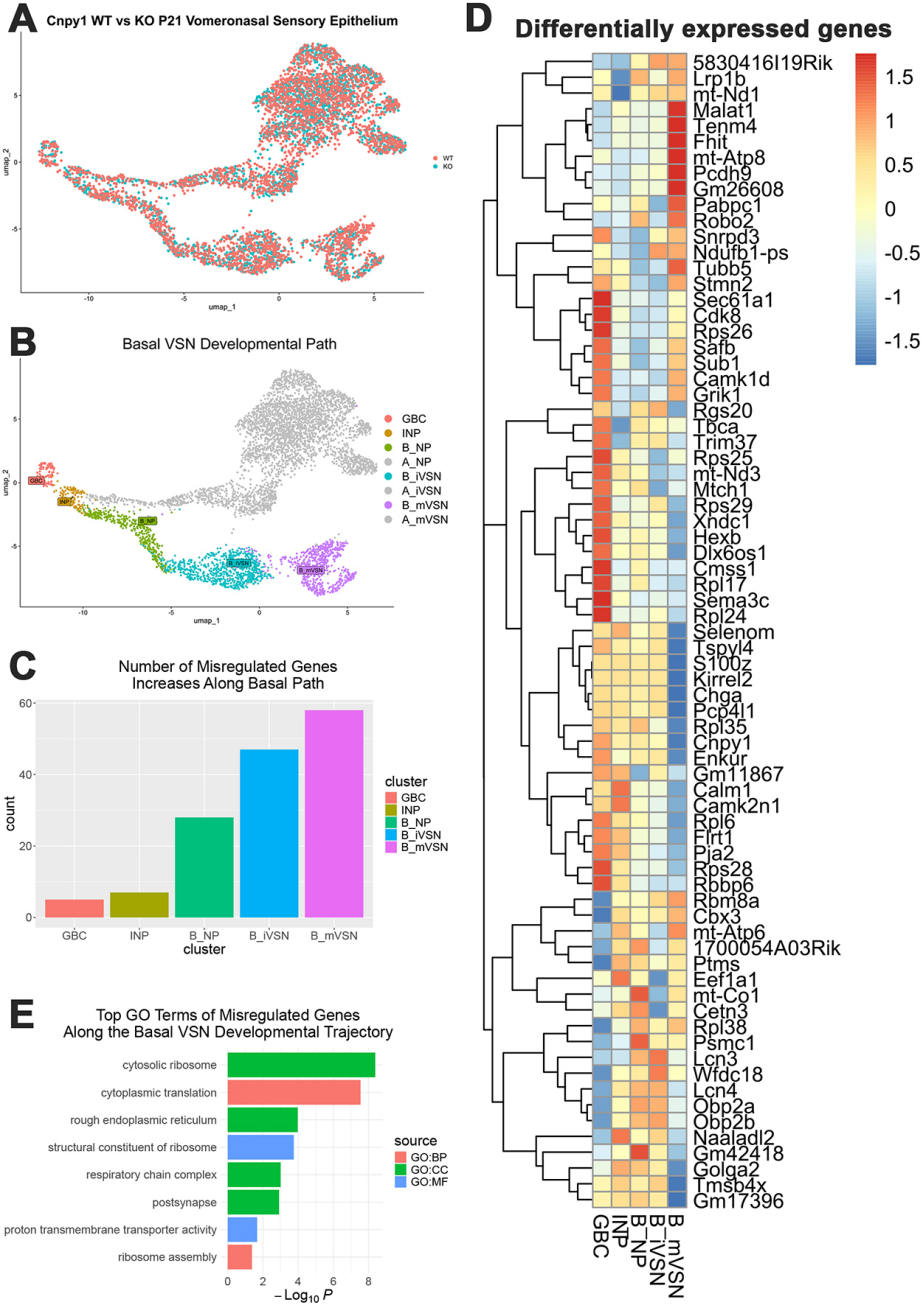
**Single-cell transcriptomics reveal stage-progressive gene misregulation along the basal VSN developmental trajectory in *Cnpy1* KO mice.** (A) UMAP overlay of wild-type (red) and *Cnpy1* KO (blue) P21 VNO scRNA-seq shows that both genotypes follow similar developmental trajectories from GBCs to mature VSNs. (B) Basal VSN differentiation progresses through five transcriptional stages: GBC, INP, basal neuronal precursors (B_NP), basal immature VSNs (B_iVSN) and basal mature VSNs (B_mVSN). (C) The number of differentially expressed genes increases with maturation, with the highest dysregulation observed in B_mVSNs. (D) For DEGs (p_adj≤0.05), KO-WT expression differences were Z-scored across stages: positive values (red) indicate higher expression in the KO and negative values (blue) indicate lower expression, revealing widespread transcriptional disruption in KO basal VSNs. (E) GO-term enrichment analysis identifies pathways related to neurodegeneration, protein folding, quality control, ribosome biogenesis, translation and cellular homeostasis.

Our experiments on Cnpy1 KOs suggest normal VNO development followed by progressive cell loss. To gain mechanistic insight, we performed scRNA-seq of P21 wild-type and *Cnpy1* KO mouse VNOs. The UMAP of wild-type (red) and KO (blue) cells (Fig. 5A) shows significant overlap, suggesting similar gene expression and development. The cells of the VNO were classified based on their maturity markers and whether they followed the apical or basal developmental pathway after divergence (Fig. 5B). For each group along the basal developmental pathway, the number of significantly misregulated genes was determined using an FDR threshold of 0.05. Notably, as maturation progresses, the number of differentially expressed genes across genotypes increases (Fig. 5C), suggesting a gradual progression of a degenerative phenotype. We conducted a GO term analysis from GBC through mature basal VSNs (Fig. 5E). A list of genes differentially expressed in mature basal VSNs is provided in Fig. 5D.

### *Cnpy1* KOs show reduced activation of V2R-expressing VSNs in response to pheromonal stimuli

**Fig. 6.**
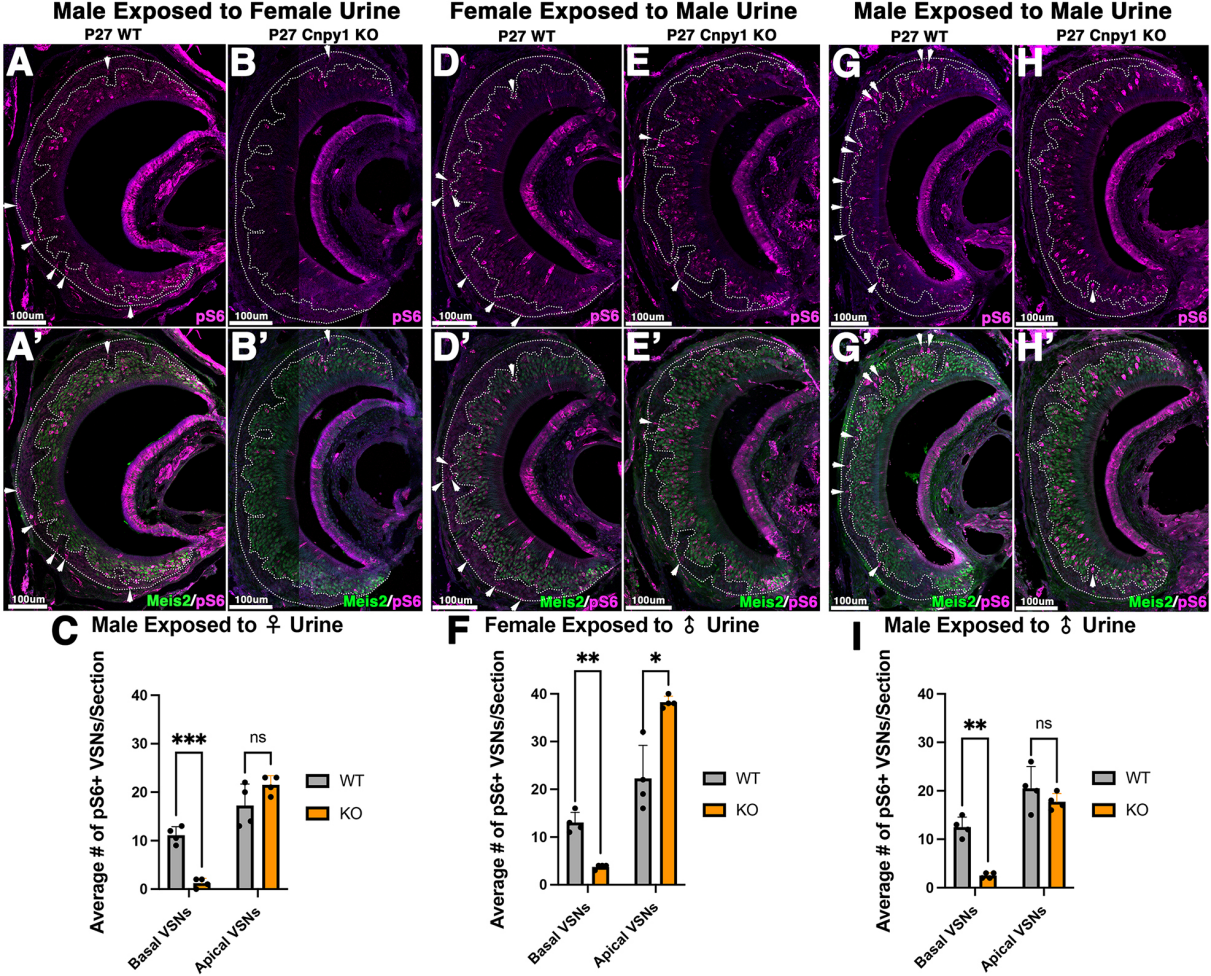
**Basal VSNs fail to activate in response to urine in *Cnpy1* KO mice.** (A-B′) In the P27 wild-type male VNO, pS6 staining reveals activation of apical (Meis2^+^) and basal (Meis2^−^) VSNs following exposure to female urine. In *Cnpy1* KO mice, apical activation is preserved, whereas activated basal pS6^+^ VSNs are nearly absent. (D-E′,G-H′) Similar reductions in basal activation are observed in female mice exposed to male urine (D-E′) and in male mice exposed to male urine (G-H′). Arrowheads indicate activated basal VSNs. (C,F,I) Quantification confirms a marked loss of basal VSN activity in KO mice (C, *P*=0.000268; F, *P*=0.002442; I, *P*=0.001466; unpaired *t*-test with Welch's correction), while apical responses remain comparable to wild type except in KO females exposed to male urine, which show increased apical activation (F, *P*=0.017505; unpaired *t*-test with Welch's correction). Scale bars: 100 μm. Data are mean±s.e.m. (*n*≥3).

Previous research indicates that the VSNs of mice with mutations impacting their ability to transduce chemosensory stimuli, such as Gαo cKO, AP-2ε KO, Smad4 cKO, ATF5 KO, Gγ8 KO and TRPC2 KO, are progressively lost during the first 2 months after birth (Chamero et al., 2011; Lin et al., 2018; Montani et al., 2013; Naik et al., 2020; Nakano et al., 2016; Stowers et al., 2002). This led us to test if the V2R-expressing VSNs of *Cnpy1* KOs were functional (Fig. 6). We exposed control and KO males and females to opposite-sex urine (Fig. 6A-F), and males were also exposed to male urine (Fig. 6G-I). Immunofluorescent staining against phosphorylated ribosomal subunit S6 (pS6) was used as a proxy for VSN signal transduction and/or activation (Fig. 6A-B′) (Silvotti et al., 2018). Quantification of pS6^+^ basal VSNs revealed significantly reduced activation in *Cnpy1* KO mice across all tested conditions (Fig. 6C-I). Notably, although the number of pS6^+^ apical VSNs was comparable to controls in males exposed to either sex, *Cnpy1* KO females showed a significant increase in apical VSN activation in response to male urine (Fig. 6F).

### *Cnpy1* KO mice exhibit elevated mRNA levels of family C V2R genes, while expression of families A, B and D remains unchanged

**Fig. 7.**
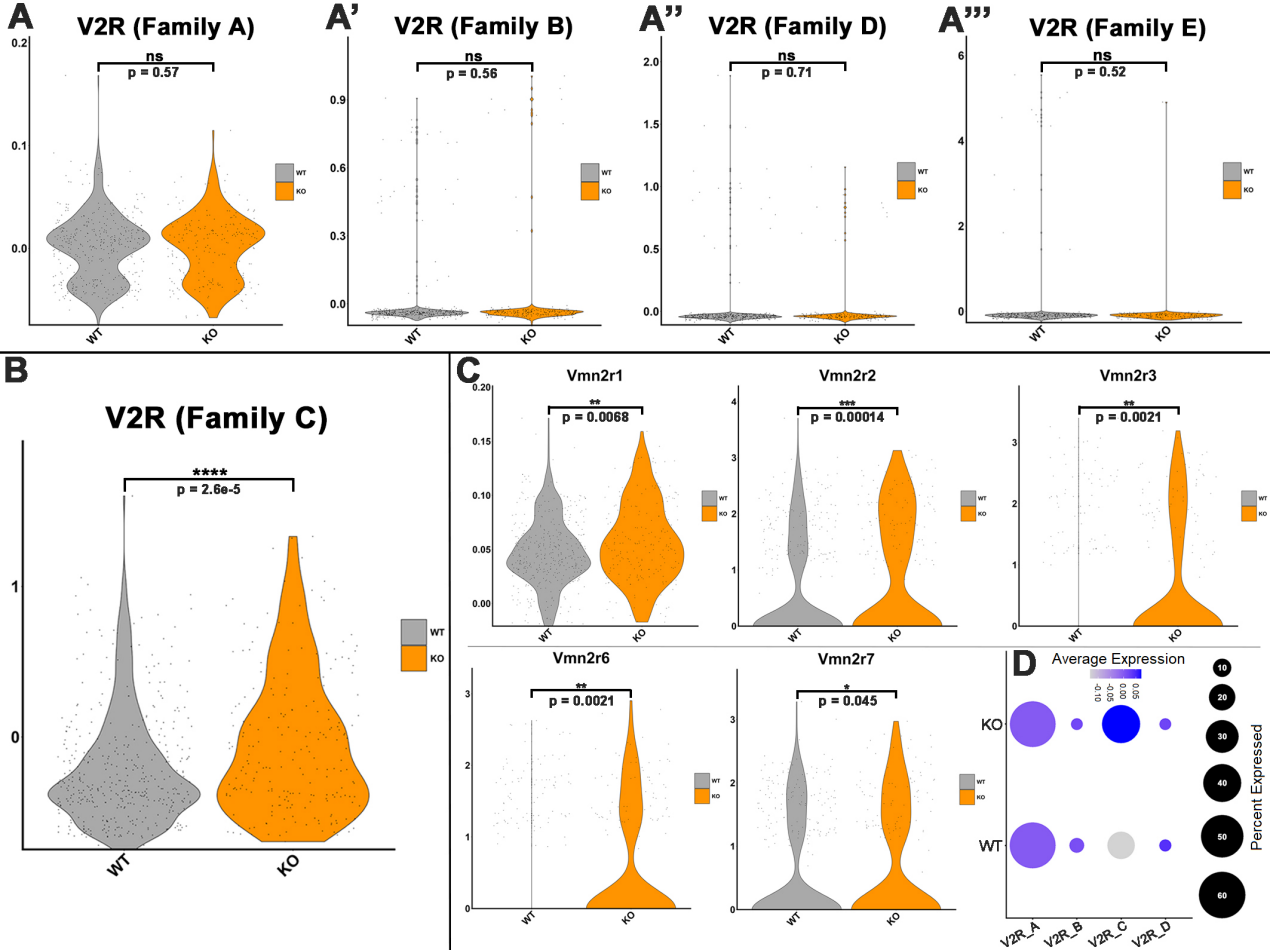
**Cnpy1 loss selectively alters V2R family C transcript levels in P21 VNO single-cell RNA-seq.** (A-C) Violin plots show AddModuleScore-based gene expression scores for representative V2R families across P21 vomeronasal sensory neurons (VSNs) from wild-type (gray) and *Cnpy1* KO (orange) mice. No significant genotype differences were detected at the global family level for V2R family C (A), family B (A′), family D (A″) or family E (A‴) (ns; exact *P* values indicated). (B) Aggregate analysis of all family C V2R transcripts revealed significantly increased expression in KO VSNs compared to wild type (****P=2.6×10^−5^). (C) Violin plots for individual family C genes (*Vmn2r1*, *Vmn2r2*, *Vmn2r3*, *Vmn2r6* and *Vmn2r7*) also showed significantly elevated expression in KO mice, indicating coordinated upregulation across multiple receptors. (D) Dot plot summarizing scaled expression (color intensity) and percentage of expressing cells (dot size) across V2R families A-D demonstrates selective enrichment of family C V2Rs in KO VSNs, whereas other V2R families remain largely unchanged. Each dot represents one cell.

Based on these findings, we assessed whether VR gene expression levels varied in the Cnpy1 KOs. Mice possess around 100 active V2R genes that, based on sequence homology, are classified into families A, B, C and D (Ishii and Mombaerts, 2011; Silvotti et al., 2007). Of note, family E in mouse includes only one gene (Francia, 2014). The V2R family-C is atypical because it is co-expressed with a V2R from another family in a monoallelic manner (Silvotti et al., 2007). Interestingly, scRNA-seq data at P21 showed that the V2R-expressing VSNs of *Cnpy1* KO mice have comparable expression levels of V2R genes from families A, B, D and E. However, we found significantly higher mRNA expression for V2R genes belonging to family C (Fig. 7B,C). Vmn2R4 and Vmn2R5, which belong to family C, have been excluded from this analysis due to low representation in our dataset. Interestingly, aside from the higher expression levels of family C V2Rs, the dot plot analysis (Fig. 7D) revealed a comparable distribution of V2Rs across the VSN populations of controls and mutants.

### *Cnpy1* KOs exhibit significantly reduced V2R2 immunodetectability at the lumen, while preserving normal VSN gross cytoarchitecture, and expressing β2m and villar markers

**Fig. 8.**
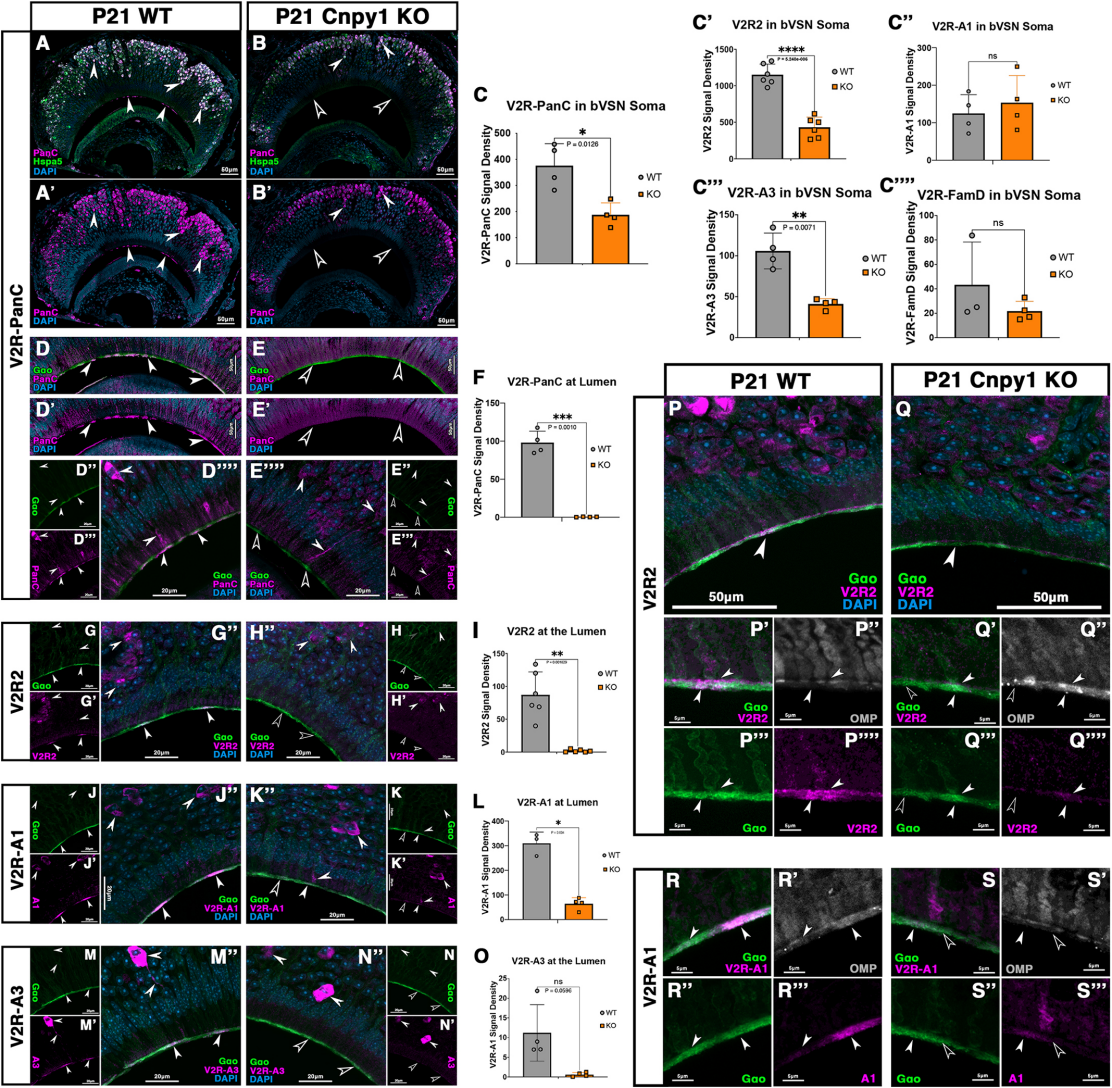
**Cnpy1 loss reduces V2R immunoreactivity in basal VSN somas and at the luminal surface at P21.** (A-B′) Coronal sections of P21 wild-type (A,A′) and *Cnpy1* KO (B,B′) VNOs were stained for V2R-PanC, HSPA5 and DAPI. HSPA5 labels the ER of basal VSNs. Arrowheads mark the luminal border; notched arrowheads indicate the basal neuronal layer. (C-C⁗) Quantification of V2R signal within HSPA5-defined ER masks shows reduced V2R-PanC (*P*=0.013), V2R2 (*P*=5.24E-6) and V2R-A3 (*P*=0.007) in *Cnpy1* KO, while V2R-A1 and V2R-FamD are not significantly different. (D-F) High-magnification luminal views confirm reduced V2R-PanC signal in mutants. (G-O) Luminal staining for V2R2, V2R-A1 and V2R-A3 with Gαo masks shows reduction of V2R2 (*P*=0.002) (G-I), V2R-A1 (*P*=0.004) (J-L) and V2R-A3 (*P*=0.0596) (M-O). (P-S‴) Super-resolution imaging highlights reduced V2R2 and partially preserved V2R-A1 in knobs and/or villi. Filled arrowheads indicate positive staining; open arrowheads indicate weak or absent signal. Data are mean±s.e.m. (*n*≥3). Data were analyzed using an unpaired two-tailed *t*-test with Welch's correction. Scale bars: 50 μm in A-B′,D,D′,E,E′,P,Q, 20 μm in D″-D⁗,G-H″,J-K″,M-N″ and 5 μm in P′-P⁗,Q′-Q⁗,R-S‴.

To determine whether the V2R mRNA differences could be detected at the protein level, we performed immunofluorescence staining on P21 wild-type and *Cnpy1* KO mice using antibodies against AP-2ε, Hspa5, Gαo and V2R2, and a battery of antibodies against different V2Rs (V2R families A1, A3, C and D) (Fig. 8; Figs S3-S7). Hspa5 was used to highlight the ER of basal VSNs and to create a three-dimensional mask of the ER using Imaris (Fig. S3). V2R2 immunofluorescent intensity in the cell soma, within the Hspa5 regions, was measured and summed. To prevent bias from varying mask sizes, the total V2R2 signal was divided by the total volume of the Hspa5 mask, yielding a V2R2 immunofluorescence density. Despite higher mRNA expression in *Cnpy1* KO mice, these mice showed an overall reduction in V2R signal density in the cell soma across all tested antibodies, except for V2R-A1 (Fig. 8C-C″″). However, the data for V2R-FamD escaped significance.

To assess whether V2Rs were detectable in the lumen, a similar masking strategy was used that leveraged immunofluorescence of Gαo, which is enriched in the microvilli (Berghard and Buck, 1996). Wild-type and *Cnpy1* KO VNOs at P21 were stained for various V2Rs with Gαo (Fig. 8D-S, Fig. S3D-F). A 3D mask of the lumen was derived from the Gαo signal, and V2R signal was measured and normalized by volume (Fig. S3; Fig. 8D-S). Notably, while signal was detectable in the soma, anti-family-D antibodies produced weak signal in the lumen and therefore were not used for quantification at this level. These measurements in the lumen indicate a notable reduction in immunodetectable V2R across all tested antibodies. High-resolution imaging of sections stained with V2R2 and V2R-A1 confirmed low detectability of V2R in the villi (Fig. 8P-S). This implies that Cnpy1 deficiency impairs protein production in the cell soma and, more significantly, hinders V2R receptor localization to the villi in the lumen.

**Fig. 9.**
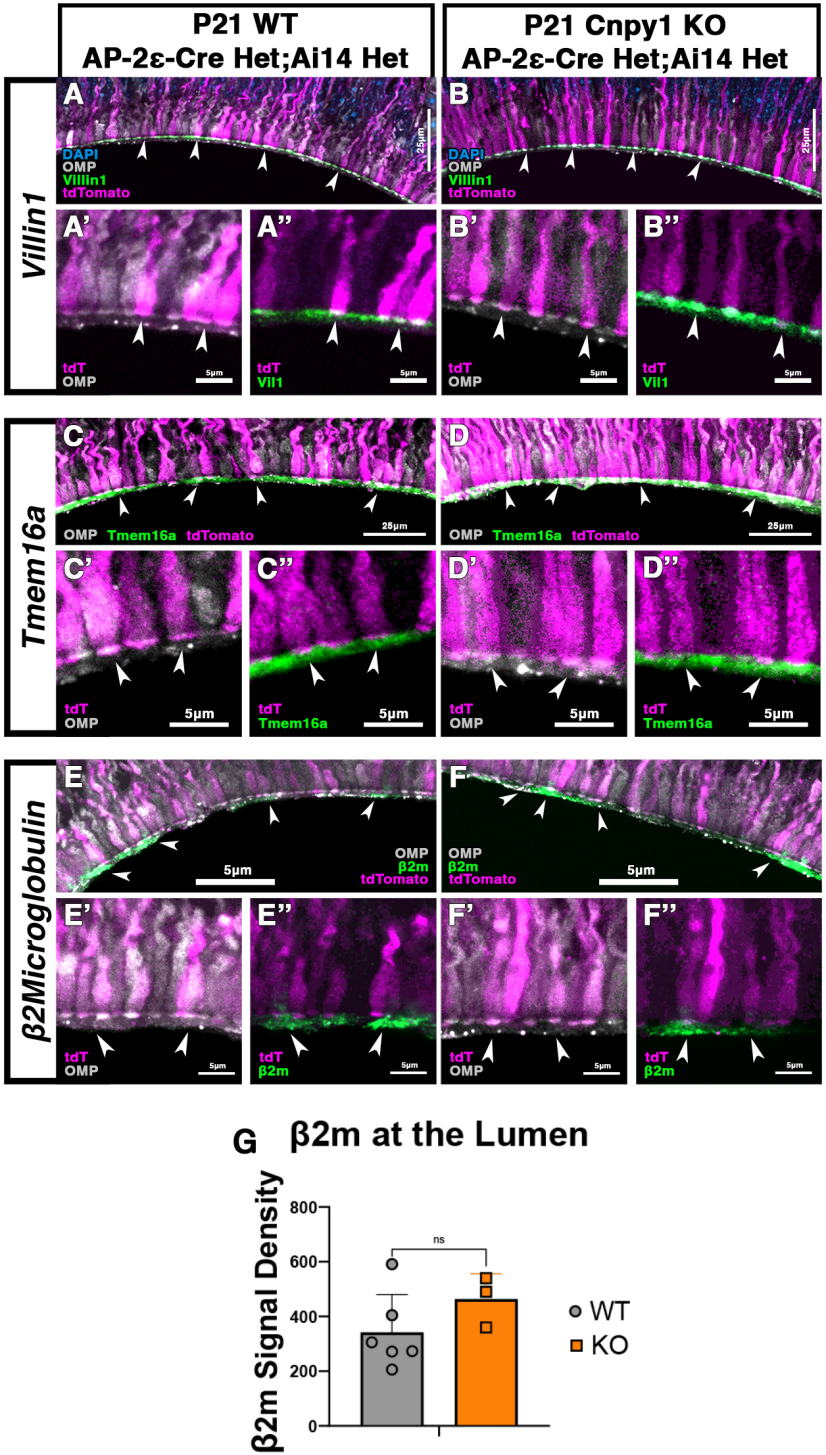
**Luminal structure and membrane-associated markers are preserved in *Cnpy1* KO VNO at P21.** (A-B″) VNO coronal sections of P21 wild-type (AP-2ε-Cre^+/−^;Ai14^+/−^) (A-A″) and *Cnpy1* KO (AP-2ε-Cre^+/−^;Ai14^+/−^) (B-B″) mice stained for villin (green), tdTomato (magenta), OMP (white) and DAPI (blue) . Arrowheads indicate the luminal border. (C-D″) VNO coronal sections stained for Tmem16a (green), tdTomato (magenta), OMP (white) in wild type (C-C″) and *Cnpy1* KO (D-D″). (E-F″) VNO coronal sections stained for β2-microglobulin (β2m; green), tdTomato (magenta), OMP (white) in wild type (E-E″) and *Cnpy1* KO (F-F″). (A′,A″,B′,B″,C′,C″,D′,D″,E′,E″,F′,F″) Higher magnification views of the luminal surface. (G) Quantification of β2m signal density within the Gαo-positive mask shows no significant difference between wild-type and *Cnpy1* KO mice. Data are mean±s.e.m. Each point represents one VNO. Statistical analyses were carried out using Welch's unpaired two-tailed *t*-test. Scale bars: 25 μm in A,B,C,D; 5 μm in A′,A″,B′,B″,C′,C″,D′,D″,E-F″.

The reduction in V2R detectability led us to check if the basal VSNs in the KOs had abnormal morphology or functions (Fig. 9). We conducted immunostaining for anti-villin, anti-TMEM16A and anti-β2-microglobulin (β2M) on control and KO samples. To better visualize the cell architecture of the basal VSNs, controls and *Cnpy1* KOs were crossed with an AP-2εCre/R26Ai14(tdTomato) background (Fig. 9). Basal VSN dendrites and knobs highlighted by tdTomato show similar gross cytoarchitecture across genotypes and maintained reactivity at the lumen for Gαo, villin 1, Tmem16a and β2M.

### V2R-expressing VSNs have higher expression of ER stress genes, and *Cnpy1* KOs show higher expression of ER stress genes and abnormal expression of ribosomal genes

**Fig. 10.**
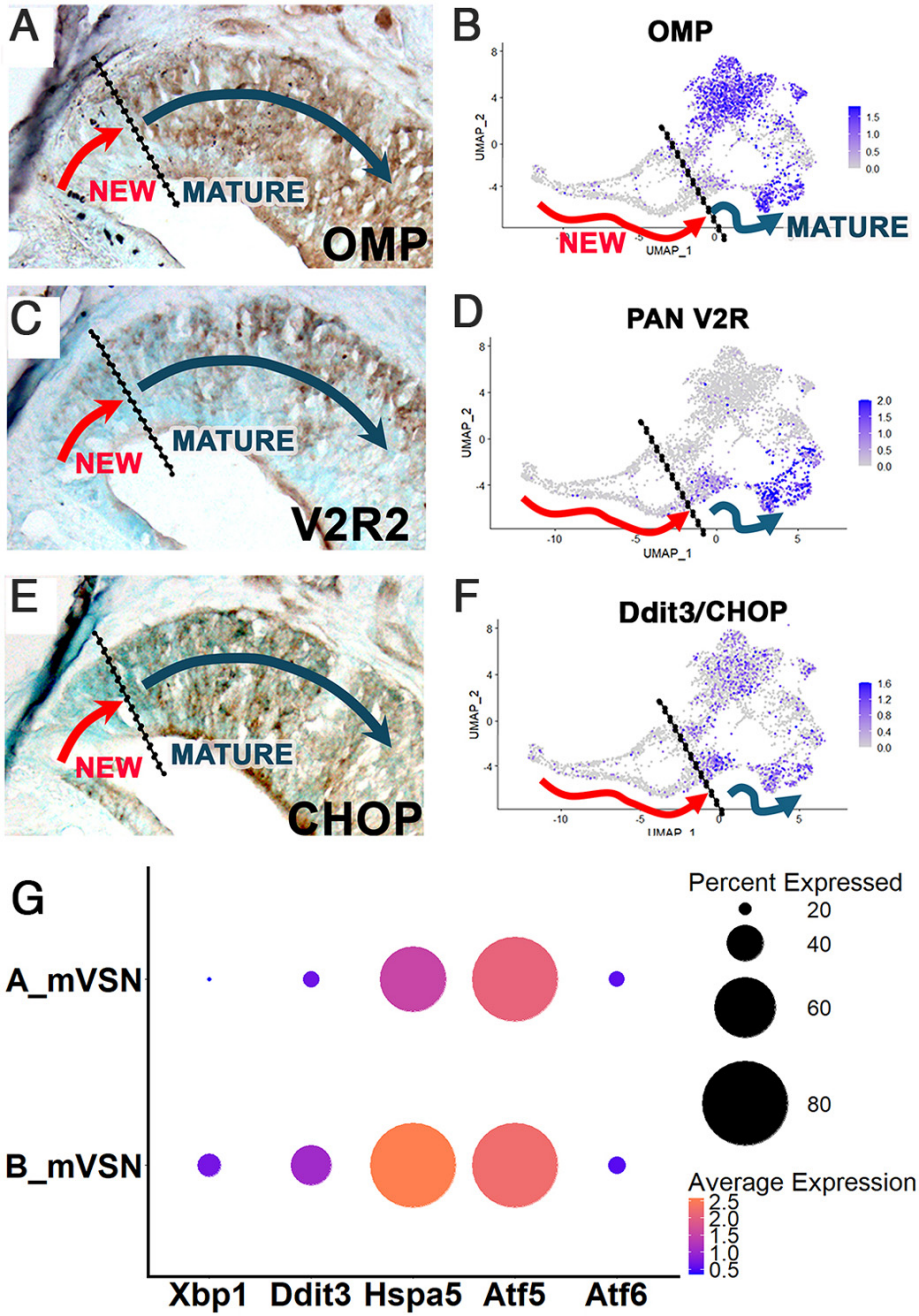
**ER stress gene expression changes in the vomeronasal organ as V1R- and V2R-expressing VSNs mature.** (A,C,E) Immunohistochemistry on P15 VNO sections shows progressive VSN maturation in the marginal zone. OMP, V2R2 and CHOP immunoreactivity increases from newly generated immature neurons toward mature VSNs, indicating coordinated upregulation of maturation markers, sensory receptors and ER-stress/UPR effectors. (B,D,F) scRNA-seq feature plots similarly show increasing OMP, pan-V2R and CHOP (Ddit3) expression along the developmental trajectory into mature V2R-expressing VSNs. (G) Dot plot from wild-type P21 scRNA-seq demonstrates elevated expression of ER stress-response genes, including Xbp1, Hspa5 and Ddit3 (CHOP) in basal V2R-expressing VSNs compared to apical V1R-expressing VSNs, suggesting increased physiological ER stress in V2R^+^ neurons.

**Fig. 11.**
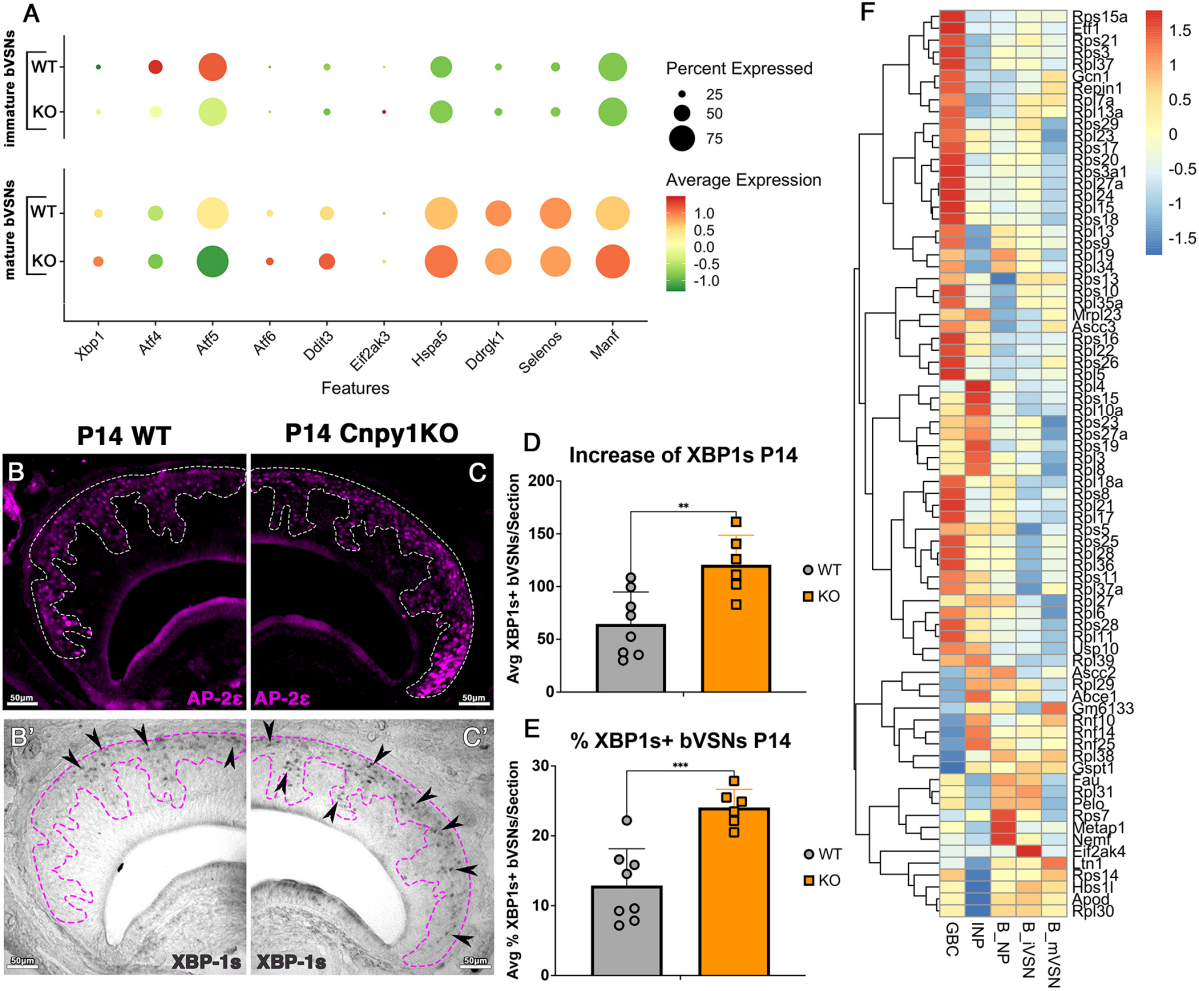
**Cnpy1 loss increases XBP1s and alters ER stress-related gene expression in basal VSNs at P14.** (A) Dot plot showing ER stress and UPR-related gene expression in immature and mature basal VSNs (bVSNs) from wild-type and Cnpy1 KO VNO. Several ER stress genes increase during maturation in both genotypes, while KO mature bVSNs show elevated Xbp1, Atf6, Ddit3, Hspa5 and Manf expression. (B-C′) P14 wild-type and KO VNO sections stained for AP-2ε and XBP1s reveal increased XBP1s^+^ cells in KO basal layers (arrowheads). (D,E) Quantification confirms increased numbers and percentages of XBP1s^+^ bVSNs in KO mice. Data are mean±s.e.m.; each dot represents one VNO (*n*≥3). **P<0.05, ***P<0.001 (unpaired two-tailed *t*-test). Scale bars: 50 μm. (F) Heatmap highlights genotype-dependent changes in ribosomal and translation-related gene expression across developmental stages.

Recent studies indicate that the physiological ER stress levels across olfactory-chemosensory neurons are determined by which odorant receptor is expressed and its specific amino acid sequences (Shayya et al., 2022). In line with this, we observed that in V2R-expressing VSNs there is an increase in ER stress gene expression coincident with initial V2R expression. In fact, immunohistochemistry and transcriptomic data revealed that Ddit3 (also known as CHOP) expression increases as the VSNs express V2R (Fig. 10A-F). Notably, by comparing classic ER stress genes such as *Hspa5*, *Ddit3* (CHOP), *Atf5*, *Atf6* and *Xbp1*, we observed that V2R-expressing VSNs exhibit higher expression of these genes than V1R-expressing VSNs (Fig. 10G). Moreover, ER stress physiologically increases from immature VSN (iVSNs) to mature VSN (mVSNs); in fact, Xbp1, Atf6, Ddit3 (CHOP), selenos, Manf and Hspa5 levels increased in both wild-type and Cnpy1 KOs as the neurons matured (Fig. 11A). Notably, these transcriptional changes in *Cnpy1* KOs affected only a percentage (∼25-50%) of the mature V2R-expressing VSN population (Fig. 10G).

Since VSNs are genetically heterogeneous and asynchronous, because the VNO continues to regenerate throughout life (Brann and Firestein, 2010), we decided to verify how these transcriptomic hints translated into detectable ER stress proteins. Therefore, we performed histochemistry using antibodies against XBP1s, the active spliced isoform of XBP1 that is produced in response to increased ER stress (Calfon et al., 2002). This analysis revealed immunoreactivity across the V2R-expressing VSNs with a significantly higher number and percentage of immunoreactive cells in *Cnpy1* KOs (Fig. 11B-E).

Notably, both ER stress levels and changes in neuronal activity can alter ribosome biology, ribosomal RNA transcription, expansion, remodeling and translation, as well as ribosomal composition (Choe and Cho, 2020; Dastidar and Nair, 2022; Fusco et al., 2021; Ron, 2002; Wolzak et al., 2022). Analysis of scRNA-seq data from wild type and *Cnpy1* KO revealed differences in the expression of multiple genes related to the ‘ribosomal cellular’ GO term (GO:0005840) (Fig. 11F).

### *Cnpy1* KO shows changes in adhesion and guidance molecules, and changes in the number and size of glomeruli in the AOB

**Fig. 12.**
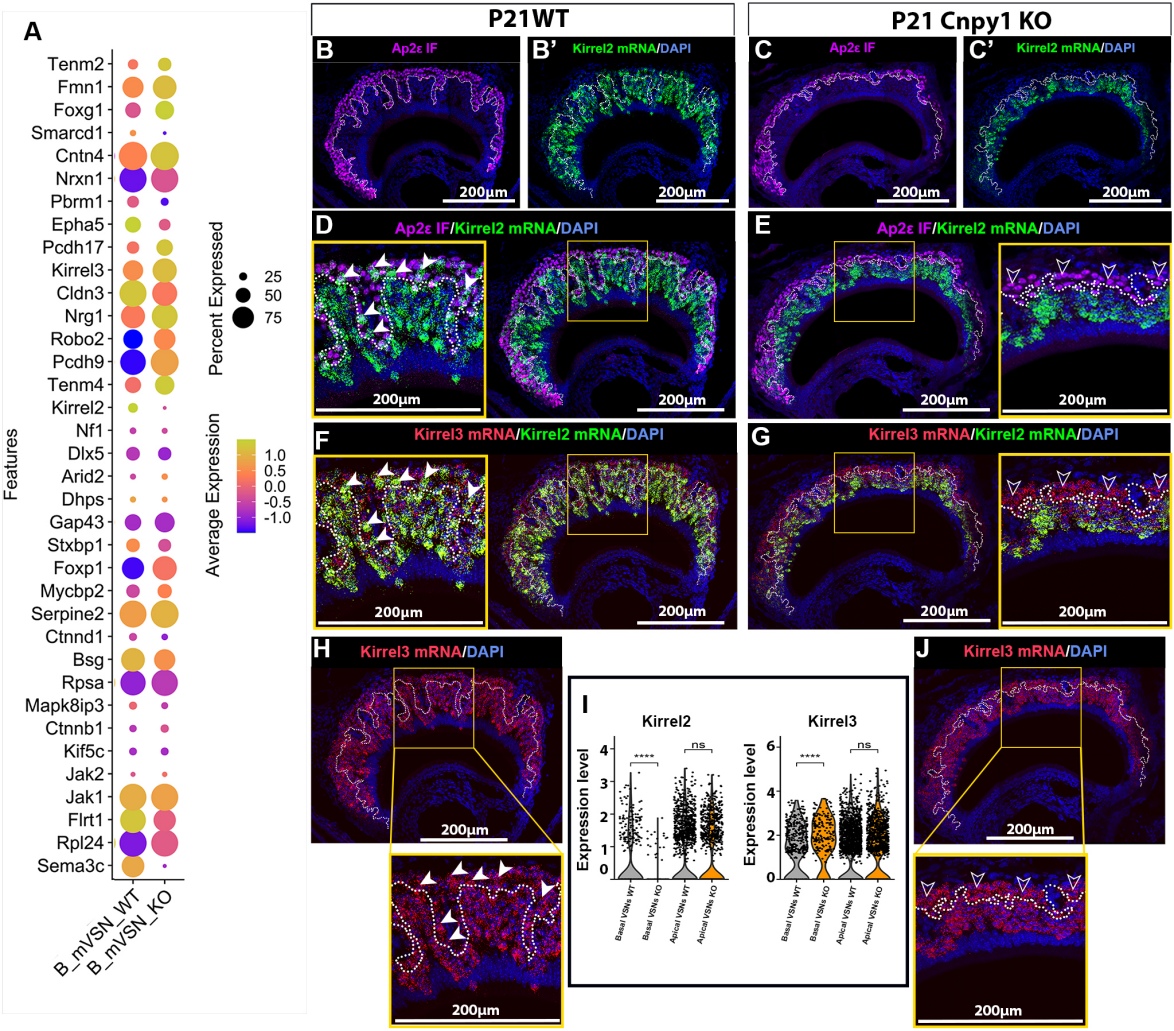
**Altered Kirrel2 and Kirrel3 expression patterns in the VNO of P21 *Cnpy1* KO mice.** (A) Dot plot from P21 scRNA-seq shows differential expression of guidance and adhesion genes in mature basal VSNs between wild type and *Cnpy1* KO. (B-C′) Coronal VNO sections from P21 wild-type and KO mice stained for AP-2ε and Kirrel2 using RNAscope. (D,E) Higher magnification views of merged images from B,B′ (D) and C,C′ (E) show broad Kirrel2 expression across wild-type basal layers, segregated from AP-2ε domains (arrowheads in D), whereas *Cnpy1* KO basal VSNs largely lack Kirrel2 signal (open arrowheads in E). (F,G) RNAscope for Kirrel3 and Kirrel2 reveals complementary expression in wild type and marked Kirrel2 loss in *Cnpy1* KO basal layers. (H-J) Kirrel3 labeling is preserved and expanded in the *Cnpy1* KO. scRNA-seq confirms significantly reduced Kirrel2 (*P*<0.000004) and increased Kirrel3 (*P*<0.0000035) in *Cnpy1* KO basal VSNs, with no changes in apical populations. Scale bars: 200 μm.

Variation in ER stress (Fig. 11) and altered neuronal activity (Fig. 6) alter the expression of genes involved in axonal guidance and coalescence (Prince et al., 2013; Shayya et al., 2022). Strikingly, single-cell analysis (Fig. 12A) at P21 revealed a near-complete loss of Sema3C mRNA and clear changes in the expression of multiple other guidance genes, including *Epha5*, *Kirrel2*, *Pcdh9*, *Pcdh17* and *Robo2*, in mature V2R-expressing VSNs of the *Cnpy1* KOs.

In non-functional VSNs, Kirrel2 expression diminishes while Kirrel3 increases as VSN activity influences their levels (Prince et al., 2013; Stowers et al., 2002). Both transcriptomic data and RNAscope show a loss of Kirrel2 mRNA in (AP-2ε^+^) V2R-expressing VSNs in the basal region, with higher Kirrel3 expression in *Cnpy1* KOs (Fig. 12A-J).

**Fig. 13.**
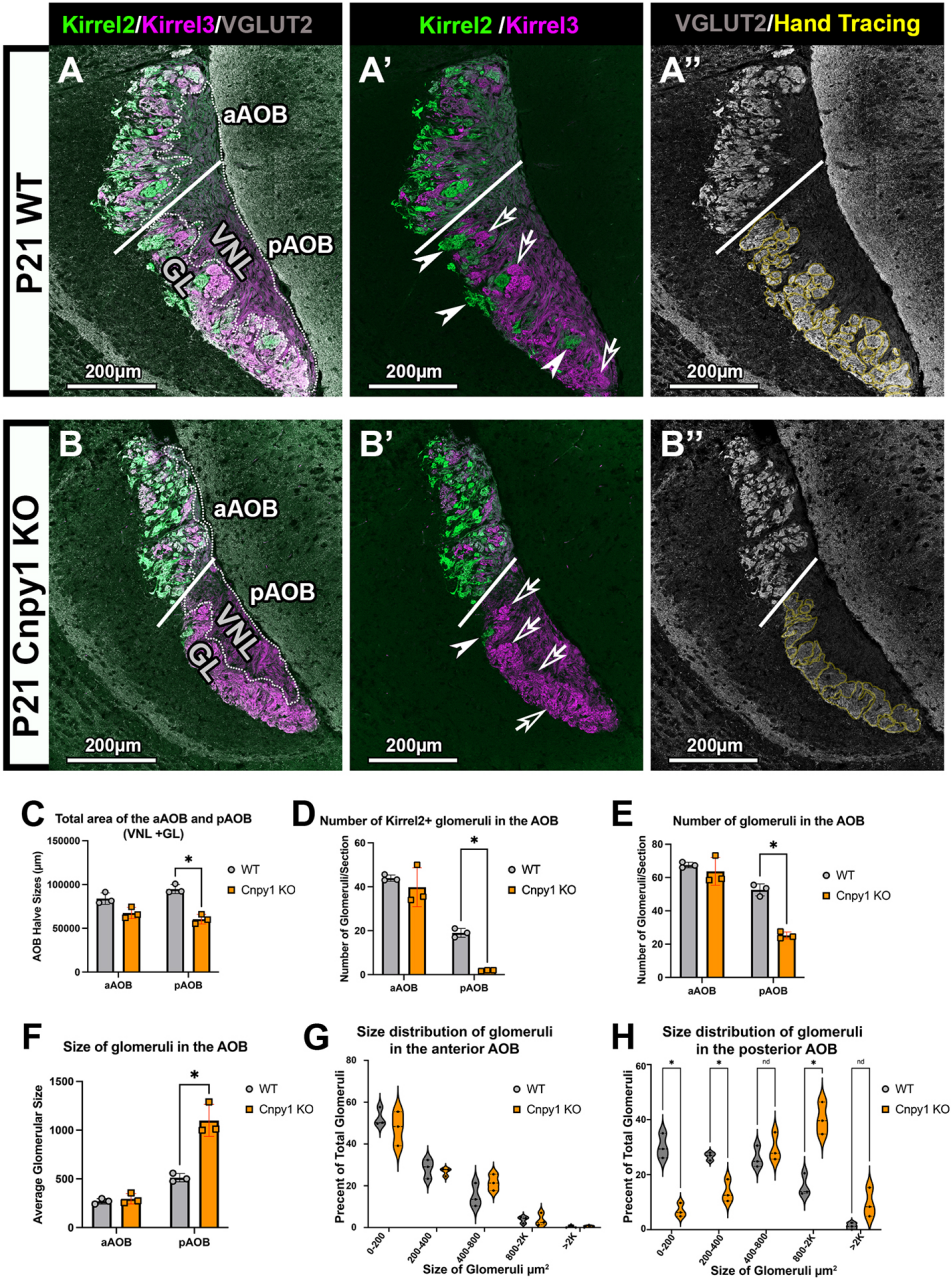
**Loss of Cnpy1 disrupts posterior AOB organization and glomerular architecture.** (A-A″) P21 wild-type AOB sections stained for Kirrel2 (green), Kirrel3 (magenta) and VGLUT2 (gray) show normal segregation of anterior (aAOB) and posterior (pAOB) glomeruli. (B-B″) P21 *Cnpy1* KO sections show a reduced pAOB territory and altered Kirrel2^+^ and Kirrel3^+^ glomerular patterning. (A″,B″) VGLUT2 labeling. Hand-tracing highlights the distribution and extent of glomeruli in wild-type and *Cnpy1* KO AOB. (C) Quantification of total AOB area (vomeronasal nerve layer, VNL+glomerular layer, GL) shows a significant reduction in the pAOB of *Cnpy1* KO mice (*P<0.001372). (D) Reduced number of Kirrel2^+^; VGLUT2^+^ glomeruli in the pAOB of *Cnpy1* KO mice (*P<0.000123). (E) Reduced number of VGLUT2^+^ glomeruli in *Cnpy1* KO mice [aAOB, *P*=0.477788 (not significant); pAOB, *P<0.000342]. (F) Increased average size of VGLUT2^+^ glomeruli in the pAOB of *Cnpy1* KO mice (*P<0.003861). Data in C-F are mean±s.e.m. (G,H) Size distribution analysis shows no preferential loss of glomeruli in the aAOB, whereas the pAOB displays selective changes across size bins [0-200 μm, *P*<0.001478; 201-400 μm, *P*<0.006615; 401-800 μm (not significant); 801-2000 μm, *P*<0.004068; >2000 μm (not significant)]. *n*=3 animals per genotype. Data were analyzed using an unpaired two-tailed *t*-test. Scale bars: 200 μm.

In line with the transcriptomic changes in the accessory olfactory bulb (AOB) immunostaining at P21 (Fig. 13) and P30 (Fig. S8) revealed an almost complete loss of Kirrel2-immunoreactive glomeruli in the posterior accessory olfactory bulb, accompanied by an abnormal expansion of Kirrel3 expression across all glomeruli. Kirrel proteins are responsible for homophilic adhesion and clustering of similar olfactory axons, ensuring the correct size and number of glomeruli in the olfactory map (Prince et al., 2013). Measurements of glomeruli number and size at P21 and P30 indicated an overall reduction in the number of glomeruli in the posterior AOB (pAOB) while the anterior AOB (aAOB) shows no significant difference in any measured metrics (Fig. 13A-B″,E; Fig. S8). We observed that the reduction in glomeruli seemed linked to an increased proportion of larger or less-defined glomeruli (Fig. 13F), although these were also decreased in number (Fig. 13G,H; Fig. S8G-H). We did find a significant loss of glomeruli number under 400 μm^2^ and an increase in number of glomeruli 800 μm^2^ and larger (Fig. 13H). Of note, overall area of both the glomerular and vomeronasal nerve layer decreased in the pAOB of Cnpy1 KOs (Fig. 13C). This phenotype appears similar to changes caused by alterations in Kirrel2 and/or Kirrel3 expression (Prince et al., 2013).

### Loss of Fgfr1 in newly formed VSNs does not recapitulate Cnpy1 KO degenerative phenotype

**Fig. 14.**
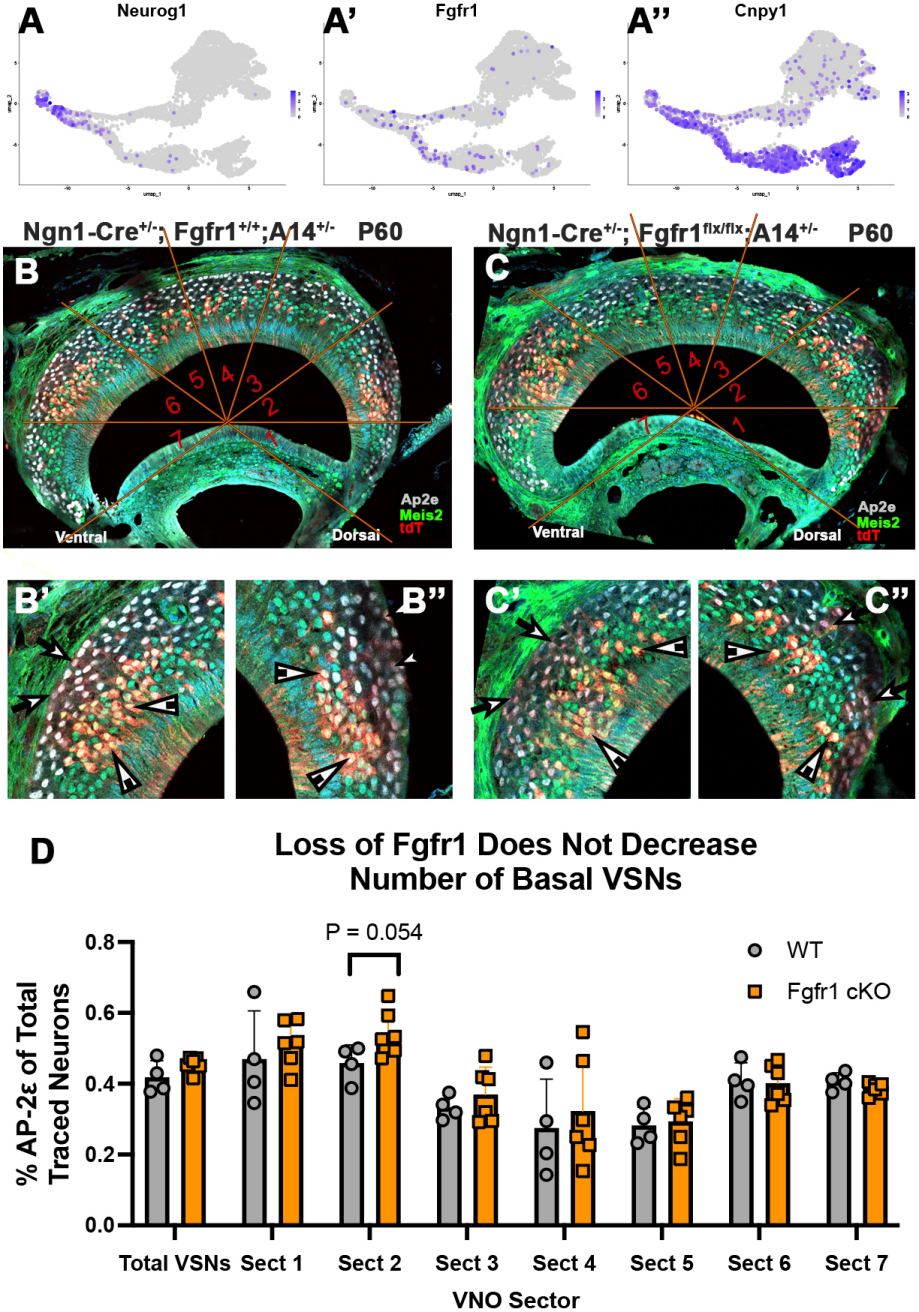
**FGFR1 loss does not phenocopy the *Cnpy1* KO and does not cause basal VSN loss.** (A-A″) Feature plots show Neurod1, Fgfr1 and Cnpy1 across the VSN UMAP. Fgfr1 is expressed in immature neurons but does not overlap with the Cnpy1-defined basal ER program, indicating distinct pathways. (B-B″) Ngn1-CreERT2; Fgfr1^+/+^; Ai14^+/−^ controls at P60 (53 days post injection) show recombination in both apical (Meis2^+^) and basal (AP-2ε^+^) VSNs; basal neurons display lower tdTomato intensity. (C-C″) Ngn1-CreERT2; Fgfr1^flx/flx^; Ai14+/− mutants show similar recombination with no basal VSN depletion; recombined cells are also present in the juvenile organ. (D) Quantification shows no significant reduction in AP-2ε^+^ basal VSNs in Fgfr1 cKO (wild type, *n*=4; knockout, *n*=6), except a minor increase in one sector. Basal VSN numbers remain stable, indicating FGFR1 loss does not phenocopy *Cnpy1* KO. Data are mean±s.e.m.

Prior to our study and another similar study, Cnpy1 has been characterized only in zebrafish, where it was proposed to be a key regulator of FGFR1 expression (Matsui et al., 2011). While Cnpy1 is expressed throughout the development of the V2R-expressing VSNs, FGFR1 is transiently expressed in newly formed VSNs. (Fig. 14A,A′). To test whether Cnpy1 phenotypes could partially result from defective FGFR1 signaling, we crossbred Fgfr1^flx/flx^ mice with Neurog(Ngn)1-CreERt2 /Fgfr1^flx/flx^/Ai14^−/−^ reporter mice. We have previously used this approach (Katreddi et al., 2022) to induce chimeric recombination in newly formed VSNs. Tamoxifen injections at P7 induced Cre-mediated recombination in newly generated apical and basal VSNs, which was monitored by the Ai14Rosa-tdTomato reporter. Mice were analyzed at P60 (53 days post-injection). Notably, we observed no significant differences between Ngn1-CreERt2^+/−^;Ai14^+/−^ and Ngn1-CreERt2^+/−^;Ai14^+/−^;Fgfr1^flx/flx^ mice. The proportion of tdTomato-labeled VSNs co-expressing either AP-2ε or Meis2 was comparable across genotypes, indicating that loss of Fgfr1 does not lead to VSN degeneration, in contrast to the pronounced neuronal loss observed in the Cnpy1 KO phenotype (Fig. 14).

## DISCUSSION

The mouse VNO has a complex sensory epithelium featuring two main neuron types and hundreds of cellular subtypes with different vomeronasal receptors expressed (Hills et al., 2024). Exhaustive research is ongoing to understand how signals are detected and which receptors identify specific ligands and how these shape behaviors (Chamero et al., 2007; Trouillet et al., 2021; Weiss et al., 2023; Wong et al., 2020). Basal VSNs express a single V2R from families A, B or D in a monogenic fashion, together with family C V2Rs, forming receptor combinations that likely define neuronal identity and functional specificity (Silvotti et al., 2007). To better understand the molecular distinctions between VSN cell types, we utilized single-cell transcriptomics and mouse genetics. Consistent with recent reports (Hills et al., 2024), we found that mature V1R- and V2R-expressing neurons differ by ∼980 genes. In line with a recent study (Devakinandan et al., 2024), the largest functional category of differentially expressed genes encodes proteins involved in ER synthesis, function and protein processing. This observation highlights an underappreciated layer of neuronal diversity: beyond transcriptional regulation, the ER is the organelle responsible for folding, modifying and trafficking proteins. The ER may itself define neuronal diversity by establishing the competence of neurons to produce certain sets of proteins within the constraints of its transcriptome.

*Cnpy1* is among the most highly expressed genes in V2R-expressing VSNs and is the most significantly differentially expressed ER-associated gene (Lin et al., 2022). Among the ER proteins with differential expression, we also found that Hspa5 (also known as BiP), which is a crucial regulator of ER homeostasis involved in protein folding and stress response modulation (Daverkausen-Fischer et al., 2022), is highly enriched in V2R-expressing VSNs in the VNO. Differential Hspa5 expression and differential expression of other ER stress genes indicate that V2R neurons have distinct basal ER stress levels and varying capacities to manage ER stress compared to V1R-expressing neurons (Figs 1,10,11).

*Cnpy1* encodes a member of the evolutionarily conserved saposin-like protein family (Cnpy1-Cnpy5 in mammals). In the VNO, we observed broad Cnpy2 expression. Apart from the findings presented here and those reported in a simultaneous independent work (Devakinandan et al., 2026), *Cnpy1* has previously been studied only in zebrafish at the midbrain–hindbrain boundary and in cell culture, where it was found to function as a chaperone influencing FGFR1 maturation and FGF8 signaling (Hirate and Okamoto, 2006; Matsui et al., 2011). Rodent Cnpy1 lacks the ER retention signal; however, immunostaining with the ER marker Hspa5 support its expression in the ER. Notably, our analysis of Fgfr1 conditional KO does not reveal any overlap between Cnpy1 loss of function and defective FGF signaling in the VNO (Fig. 14), suggesting that, in the VNO, Cnpy1 may have other functions or that compensatory mechanisms may mask the loss of Fgfr1.

In the VNO, Cnpy1 mRNA is expressed from globose basal cells through mature V2R-expressing VSNs (Fig. 2). Cnpy1 transcripts were also transiently detected along the V1R trajectory in the GBCs of the MOE and in the hindbrain, but we could never detect the proteins (Fig. S2). These data suggest that the translation of Cnpy1 protein is primarily, if not entirely, restricted to the V2R-expressing VSNs. Based on these observations, we cannot speculate on functions for Cnpy1 outside the vomeronasal system.

Morphological and transcriptomic analyses of control and Cnpy1 KO mice show that the loss of Cnpy1 does not impact proliferation or hinder VSN neurogenesis and maturation (Fig. 4). However, a pathological phenotype develops gradually: quantitative assessments from P14 to P60 reveal a slow decline in basal VSNs, indicating a degenerative process. V1R-expressing neuron counts slightly increased at P60, possibly due to cell redistribution or compensatory proliferation after V2R loss.

Our quantifications at P14 and P21 do not indicate a significant difference in the number of apoptotic cells in the KOs. Notably, VSN cell loss not associated with a proportional increase in apoptosis has been previously reported in other mice with V2R-expressing VSN loss of function (Montani et al., 2013). As VSNs have asynchronous development and aging, we cannot exclude the possibility that apoptosis escaping statistical significance plays a role in the loss of VSNs in the KOs. Our data partially differ from recent findings by others (Devakinandan et al., 2026). However, we believe that the difference between the two studies may emerge from how individual points were used for statistical analysis.

Functional testing showed that V2R-expressing neurons in *Cnpy1* KO mice fail to respond to stimuli. This prompted us to examine receptor expression and trafficking. Basal neurons in the mouse VNO express V2Rs belonging to families A, B and D, together with one member from family C (Silvotti et al., 2007). Though our scRNA-seq data at P21 lacked sufficient cells to reliably compare expression differences among individual V2R genes, we could still perform comparisons between grouped genes belonging to families A, B and D. Moreover, we could analyze the expression of V2R genes of family C between controls and mutants. Grouping V2Rs by family showed selective increases in family C receptor mRNA levels in Cnpy1 KO animals, while the relative mRNA expression of V2R receptors from families A, B and D stayed comparable across genotypes. Our finding contrast with results from bulk transcriptomic data in *Cnpy1* KO at P60, which reported reduced mRNA levels for most V2R genes (Devakinandan et al., 2026). We believe that conclusions drawn from bulk-seq analyses might be misleading due to a reduced number of V2R-expressing VSNs. We believe that bulk results indicate this simply represents neuronal loss rather than genuine changes in V2R transcript levels. Our P21 data indicate that Cnpy1 KOs do not experience a biased loss of VSNs related to the expression of a specific V2R gene family at this stage.

We performed immunostaining using previously published antibodies against V2R family C (PanC, V2R2) and family A members V2R-A1 and V2R-A3, as well as family D (Silvotti et al., 2011). While family D immunoreactivity was readily detected in the soma, the luminal signal was too weak for reliable quantification. In *Cnpy1* KO mice, we observed a marked loss of family C V2R detectability and a significant reduction in family A V2Rs at the dendritic lumen. Somatic immunoreactivity was also reduced for V2R2, PanC, V2R-A3 and family D receptors. By contrast, V2R-A1 staining in the soma was preserved across genotypes, although its luminal signal was significantly decreased. The reduction in family C V2R protein in the KO contrasts with the increase in family-C V2R mRNA levels, whereas family A V2R mRNA levels remained unchanged (Fig. 7). These findings suggest that Cnpy1 loss impairs the translation, processing or trafficking of family C V2Rs, with broader effects on additional V2R families. The selective increase in family C V2R transcripts further suggests the presence of a compensatory feedback mechanism and a distinct regulatory control for the family-C V2Rs.

Our data support that some V2Rs can reach the villi; however, we found marked and consistent reduction for all the antibodies tested that were detectable in the lumen, especially those of family C (V2R2 and PanC) (Fig. 8, Figs S3 and S4). While V2R-A1 immunoreactivity appeared unchanged in *Cnpy1* KO soma, its expression at the lumen was drastically reduced, consistent with reduced protein synthesis and trafficking in *Cnpy1* KOs. Although some V2Rs may still reach the lumen in the absence of Cnpy1, our findings suggest that this occurs less efficiently. Importantly, labeling of villin and Tmem16a indicated comparable knob integrity between genotypes, and β2-microglobulin (B2M), a component implicated in V2R trafficking, remained detectable in both controls and mutants, arguing against structural defects as the cause of reduced luminal signal. Reduced activation of V2R neurons in response to external stimuli suggests a loss of function in basal VSNs in *Cnpy1* KOs. It remains to be determined whether family-C V2R expression falling below a certain threshold is sufficient to impair the ability of the V2R-expressing VSNs to detect stimuli. Future studies should explore this by using strategies to ablate the entire family C. Interestingly, we observed increased activation of V1R-expressing VSNs in females exposed to male urine. A possible explanation could be the loss of competition from V2R-expressing VSNs for shared ligands.

ER stress differs physiologically among cells with different GPCRs (Shayya et al., 2022). In line with this, we observed increased expression of ER stress genes as VSNs mature and express their receptors. Moreover, we found higher levels of ER stress genes in V2R-expressing VSNs compared to V1R-expressing VSNs. In *Cnpy1* KO mice, we detected increased expression of ER stress-response genes in only 10-25% of the V2R population, suggesting that Cnpy1 loss of function elicits a heterogeneous and asynchronous ER stress response rather than a uniform effect across all V2R neurons. Accordingly, immunostaining for XBP1s showed increased immunoreactivity in *Cnpy1* KOs, recapitulating the increase of ER-stress genes observed in our transcriptomic analysis (Fig. 11). We propose that the high cellular heterogeneity and asynchronous development of VSNs limit the sensitivity of bulk analyses, masking changes in specific neuronal populations. Accordingly, our findings differ from those of bulk transcriptomic studies, where such changes might have remained undetected. (Devakinandan et al., 2026).

Our scRNA-seq data indicate altered expression of elongation factors and a large number of ribosome-associated proteins in *Cnpy1* KOs. Interestingly, both ER stress and neuronal activity can regulate mRNA selection, ribosome engagement and the molecular composition of ribosomes themselves (Biever et al., 2020; Fusco et al., 2021; Harding et al., 2000; Vattem and Wek, 2004). This indicates that variations in gene expression related to ribosomal components and translation may mirror both ER stress and the diminished ability of neurons to perceive stimuli. ER stress levels and neuronal activity influence the expression of adhesion molecules and axon guidance cues (Prince et al., 2013; Shayya et al., 2022). Consistent with other studies, our data show that *Cnpy1* KO mice exhibit reduced function of basal VSNs, confirming that the disruptions in sensory signal transduction can further affect circuit development. *Cnpy1* KO mice exhibit a marked reduction in Kirrel2 and an increase in Kirrel3 expression within V2R-expressing VSNs, accompanied by disorganized glomerular architecture in the posterior accessory olfactory bulb (AOB). These changes include variations in glomerular number and size, indicating cell loss and differences in glomerular dimensions and definition. These morphological alterations are consistent with existing research on the roles of Kirrel proteins in axonal diversity and selectivity in axonal coalescence (Prince et al., 2013).

Basal VSN identity is established through sequential activation of Notch signaling, Bcl11b and, ultimately, AP-2ε (Katreddi et al., 2022). Ectopic AP-2ε expression in apical neurons demonstrated its role as a master regulator, inducing ∼30% of basal-enriched genes, including the ER proteins Cnpy1 and Calr4, while repressing apical-enriched genes, including Calr and Nsg1 (Lin et al., 2022). These findings suggest that the ER-protein repertoire of V2R-expressing VSNs is an integral component of the basal gene regulatory network governed by AP-2ε, with Cnpy1 emerging as a factor required for maintaining V2R proteostasis. Disrupting a neural type-specific ER protein repertoire can affect receptor trafficking, which, in turn, can alter downstream signaling and activity-dependent transcriptional programs, including the regulation of cell-adhesion molecules. The changes we observed after Cnpy1 loss of function suggest a mechanistic link between ER proteostasis and loss of glomerular organization in the accessory olfactory bulb. While this manuscript was in revision, the independent study by Devakinandan and co-workers reached both convergent and divergent conclusions from those presented here, using a different *Cnpy1* KO mouse and different experimental approaches (Devakinandan et al., 2026).

GPCRs are the largest and most diverse group of receptors in eukaryotes (Garcia De Las Bayonas and King, 2025). Our results suggests that distinct cell types may possess specialized ER compositions tailored to support the proper processing of these evolutionarily diversified receptors. A systematic investigation of the ER diversity across neurons could open new perspectives on the evolution of gene regulatory networks and developmental processes, and identify new mechanisms underlying neuronal degeneration and aging (Roux et al., 2023).

### Limitations

A major limitation of this study is that we cannot disentangle the relative contributions of ER stress and reduced neuronal activity from the observed changes in gene expression and connectivity. Although *Cnpy1* KO mice showed altered expression of genes linked to ribosome function and translation, we did not directly assess translational output, e.g. by ribosome profiling, and therefore the corresponding protein-level consequences remain unresolved. In addition, the limited number of cells prevented a comprehensive analysis of mRNA changes across the full V2R gene repertoire. Finally, although reduced detection of family C V2Rs in *Cnpy1* KO mice correlates with impaired basal VSN function, this association does not establish causality. Defining whether family C V2Rs are directly required for stimulus detection or receptor trafficking, or both, will require targeted genetic approaches.

## MATERIALS AND METHODS

### The Cnpy1KO(C57BL/6J-Cnpy1^em1cyagen^) mouse line

The Cnpy1KO(C57BL/6J-Cnpy1em1cyagen) mouse line was generated for us by Cyagen Biosciences using CRISPR/Cas9-mediated deletion. Briefly, four gRNAs (ACTTGTTCACTGGACCCGCGTGG, TTGTAACAAATGCCGTGGGCTGG, GATGAGGAACGCCTCGGACTGGG and AGAGCATTAAATCTGGCCGTGGG) targeting sequences flanking the critical coding region of Cnpy1 were co-injected with Cas9 mRNA into C57BL/6J zygotes. This strategy produced a precise 6236 bp deletion, confirmed by PCR and Sanger sequencing. Genotyping was performed using primers F1 (5′-CTTCAGCCTCTACTCAACAACTTTT-3′) and R1 (5′-CTGTGCGACGTATTCACTCTTTTA-3′), yielding a 490 bp product for the knockout allele and a 604 bp product for the wild-type allele. A secondary primer set [F2: 5′-TTCCAGATCTATGTGGCACTCCTA-3′ and R1 (above)] was used to distinguish all genotypes: 604 bp (wild type), 490 bp+604 bp (heterozygous) and 490 bp (homozygous KO). *Cnpy1* KO mice were viable and fertile. Heterozygous mice were intercrossed to establish the colony and generate *Cnpy1^−/−^* mice for experiments.

### Additional mouse lines

B6;129P-Tg(Neurog1-cre/ERt2)1Good/J, Strain #:008529 (Koundakjian et al., 2007) and the Rosa26 tdTomato reporters Ai14/B6.Cg-Gt(ROSA)26Sortm14(CAG-tdTomato)Hze/J (Madisen et al., 2010) were purchased from the Jackson Laboratory. B6.129S4-Fgfr1tm5.1Sor/J, Strain #:007671 (Hoch and Soriano, 2006) was donated to us by Dr Pei-San Tsai (University of Colorado Boulder, CO, USA) and AP-2εCre/Tfap2etm1(cre)Will (Feng et al., 2009) was donated to us by Trevor Williams (University of Colorado Anschutz Medical Campus, Denver, CO, USA). These mice were backcrossed on C57BL/6J for over 10 generations. All experiments using animals were completed in accordance with the guidelines of the Institutional Animal Care and Use Committee (IACUC) at the University at Albany (SUNY, USA).

### Tissue preparation, immunohistochemistry and immunofluorescence

Mice for immunohistochemistry and immunofluorescence were injected with sodium pentobarbital prior to transcardial perfusion with PBS, followed by 4% paraformaldehyde (PFA) in PBS. Brains were separated from noses and post-fixed in 1% PFA overnight at 4°C, while noses were post-fixed in 4% PFA overnight at 4°C. Brains were cryoprotected in 30% sucrose in PBS at 4°C overnight, and noses of animals older than P21 were incubated in 500 mM EDTA in PBS for decalcification. After EDTA incubation, noses were cryoprotected in 30% sucrose in PBS.

E14.5 embryos were collected from time-mated females; the observation of a vaginal plug was designated as embryonic day 0.5 (E0.5). Collected embryos were fixed in 3.7% formaldehyde in PBS at 4°C for 2 h and then transferred to 30% sucrose in PBS overnight at 4°C.

Samples were embedded in OCT and stored at −80°C prior to cryosectioning into serial sections on Superfrost Plus charged slides. Depending on the antibody, heat-induced epitope retrieval was performed on slides using citric acid buffer at 95°C for 15 min prior to blocking and primary antibody incubation (for selected antibodies marked with an asterisk). Primary antibodies were incubated at 4°C overnight. Secondary antibodies were used at a 1:1000 concentration and incubated for 1 h at room temperature.

The following primary antibodies were utilized: chicken anti-Vglut2 (1:3000, 135416, Synaptic Systems); goat anti-Kirrel2 (1:500, AF2930, R&D Systems); mouse anti-Kirrel3 (1:500, 75-333, NeuroMab); rabbit anti-Gαo (1:500, PA5-59337, Invitrogen); mouse anti-Gαo (1:200, 271 111, Synaptic Systems); mouse anti-Meis2 63-T* (1:500, sc-81986, Santa Cruz); goat anti-AP-2ε* (1:500, AF5060, R&D Systems); rabbit anti-Cnpy1* (1:100, NBP2-82720, Novus Biologicals); rabbit anti-cleaved caspase 3* (1:500, AB3623, Millipore); rabbit anti-Hspa5-488 (1:500, CL488-11587, ProteinTech Group); rabbit anti-pS6* Ser244/247 (1:2500, 44-923G, Invitrogen); rabbit anti-DsRed (1:500, 600-901-379S, Rockland); goat anti-DsRed (1:1000, 200-101-379, Rockland); rabbit anti-CHOP* (1:500, 15204-1-AP, Proteintech); rabbit anti-Tmem16a* (1:200, ab53212, Abcam); rabbit anti-Villin1 (1:2000, ab130751, Abcam); rabbit anti-Ki67* (1:750, AB9260, Millipore); rabbit anti-β2m (1:1250, PA5-81587, Invitrogen); goat anti-OMP (1:4000, 54410001, Wako); and rabbit anti-Xbp1-s* (1:200, 40435, Cell Signaling Technology). The V2R antibodies used were kindly provided by Roberto Tirindelli (University of Parma, Italy): rabbit anti-V2R2* (1:5000), rabbit anti-V2R-A1* (1:200), rabbit anti-V2R-A3* (1:200), rabbit anti-V2R-PanC* (1:2000) and rabbit anti-V2R-FamD* (1:500). The host species of all secondary antibodies used was donkey: anti-chicken Alexa Fluor 680 (1:1000, 703-625-155, Jackson ImmunoResearch), anti-goat Alexa Fluor 488 (1:1000, 705-545-003, Jackson ImmunoResearch), anti-goat Alexa Fluor 647 (1:1000, 705-605-003, Jackson ImmunoResearch), anti-goat Alexa Fluor Plus 680 (1:1000, A32860, Thermo Fisher/Invitrogen), anti-goat Biotin-SP (1:1000, 705-065-147, Jackson ImmunoResearch), anti-mouse Alexa Fluor 488 (1:1000, 715-545-151, Jackson ImmunoResearch), anti-mouse Alexa Fluor 594 (1:1000, 715-585-150, Jackson ImmunoResearch), anti-mouse Alexa Fluor 647 (1:1000, A31571, Thermo Fisher/Invitrogen), anti-mouse Alexa Fluor Plus 680 (1:1000, A32788, Thermo Fisher/Invitrogen), anti-rabbit Alexa Fluor 488 (1:1000, 711-545-152, Jackson ImmunoResearch), anti-rabbit Alexa Fluor 594 (1:1000, 711-585-152, Jackson ImmunoResearch) and anti-rabbit Biotin-SP (1:1000, 711-065-152, Jackson ImmunoResearch).

DAB/NiDAB reactions were performed as described by Forni et al. (2011). Slides were treated with 30% methanol, 0.1% H_2_O_2_ to block endogenous peroxides. Following incubation with primary antibody and corresponding biotinylated secondary antibody, sections were incubated with ABC Peroxidase Solution (PK-6100, Vector) for 1 h at room temp prior to DAB reaction. Nickel (II) sulfate heptahydrate (Sigma) was used to intensify the DAB reaction (black). Bright-field images were taken using a Leica DM4000 B LED fluorescence microscope equipped with a Leica DFC310 FX 422 camera.

### RNAscope

Single-molecule fluorescence *in situ* hybridization was performed using the RNAscope Multiplex Fluorescence v2 assay and the Kirrel2 (RNAscope Probe- Mm-Kirrel2-C1 #491421-C1) and Kirrel3 (RNAscope Probe- Mm-Kirrel3-C2 #463651-C2) probes from ACD Bio. The assay was performed on 18 μm fixed-frozen P21 mouse cryosections by following the manufacturer's protocol.

### *In situ* hybridization

A Calr4 double-stranded gene block was purchased from Integrated DNA Technologies (IDT) containing the cDNA of interest obtained by Genepaint and flanked by an upstream T7 promoter and downstream SP6. *In situ* hybridization was performed as described previously (Lin et al., 2022).

### Tamoxifen preparation and Use

Tamoxifen (Sigma–Aldrich, CAS # 10540–29–1), was mixed and dissolved in corn oil at a concentration of 20 μg/μl. Pups were injected at 80 mg tamoxifen/kg body weight. Animals were then perfused at the appropriate time point.

### Urine exposure and pS6 assay

Mice were weaned, isolated and habituated in clean static cages for 3 days prior to urine exposure. The mice were placed in a cage saturated with urine from either males or females, according to the experimental paradigm, for 90 min prior to perfusion. Cells immunoreactive for pS6 above a set threshold were counted to determine activity levels of VSNs in wild-type mice and *Cnpy1* KO mice at age P24.

### Vomeronasal organ single-cell mRNA sequencing

Two animals per genotype were euthanized with CO_2_, and their VNOs removed and placed immediately in PBS on ice. The VNO was removed from the cartilaginous capsule and digested in neuronal isolation enzyme/papain, DNAse I, collagenase A and L-cysteine for ∼15 min at 37°C. Digestion was stopped using 100% FBS and the cell suspension filtered with a 70 μm followed by a 40 μm cell strainer. Cells were pelleted at 400 ***g*** at 4°C for 5 min and resuspended in FBS just prior to carrying out the 10X protocol.

The 10X Cell Ranger pipeline was used to align FASTQ files and generate Filtered Feature Matrices. Cells were filtered out if the percentage of mitochondrial reads was higher than 12%. Principal component analysis (PCA) enables nearest neighbor calculations and clustering of cells based on gene expression, while non-linear dimensional reduction, uniform manifold approximation and projection (UMAP) allows visualization. Cell identity is assigned based on known markers and VSN-specific clusters can be grouped modularly for analysis. The SCTransformV2 (Choudhary and Satija, 2022) Seurat method was used to scale and normalize aligned reads, addressing technical variances in read depth across samples. Clustering resolution was optimized using the Clustree (Zappia and Oshlack, 2018) package to clarify cluster boundaries and facilitate targeted exploration of developmental space. Differential gene expression (DGE) and representation of UMAPs, dot plots and heatmaps were performed using the FindMarkers function within the Seurat package utilizing Wilcox ranked sums test. GO Term analysis was performed using the Gost function from the gProfiler package (Kolberg et al., 2023). Olfactory epithelium single-cell sequencing data was previously published by Li et al. (2024) and is available in the CNGB Sequence Archive (CNSA) of China National GeneBank DataBase (CNGBdb) under the accession number CNP0004813.

### Olfactory epithelium single-cell mRNA sequencing

Four 3-month-old olfactory epithelia single-cell sequencing data were used from Li et al. (2024). The datasets were individually processed using cell cycle regression and a doublet finder (McGinnis et al., 2019). Quality control (QC) and downstream analysis were performed using the Seurat (5.4.0) package in RStudio. Basic filtering was carried out in which all genes that were expressed in at least three cells and all cells with at least 200 detected genes were included. QC was based on the number of genes and percentage of mitochondrial genes – all cells that expressed >7500 genes, <100 genes and >25% mitochondrial genes were not included in the analysis. The top 2000 highly variable genes across the population were selected to perform PCA and the first 50 principal components were used for cell clustering, which was then visualized using UMAP. Neuronal progenitors, neuronal precursors, immature olfactory sensory neurons (OSNs) and mature OSNs were identified using known markers. These clusters were analyzed for gene expression using feature plots and correlation matrices. Gene correlations were determined using the Pearson correlation coefficient.

### Quantification, figure preparation and statistical analysis of microscopy data

Confocal microscopy images were captured on a Zeiss LSM 710 and 980. Epifluorescence images were acquired using a Leica DM4000 B LED fluorescence microscope equipped with Leica DFC310 FX camera. Composite images and figure preparation were prepared using Adobe Photoshop 24.7.0. Prism 10.6.0 was used for all statistical analyses, including calculation of mean values and s.e.m. Two-tailed, unpaired *t*-tests were performed for all comparisons and *P*<0.05 was considered statistically significant. Sample sizes and *P*-values are reported as individual data points in each graph and/or in the figure legends. Percentage values were derived following log10 transformation of the data.

### VNO cell counts and AOB quantifications

For AOB quantifications, established markers were used to determine anterior (Nrp2^+^, Gαi2^+^ and Kirrel2^+^) and posterior (Robo2^+^, Gαo^+^ and Kirrel3^+^) domains of the AOB, and VGLUT2 immunostaining was used to define the glomerular layer. Cell counts were obtained by both manual assessment and using a software for automated quantifications that we have previously described (Bahreini Jangjoo et al., 2021). For VNO quantifications, VNOs were collected at a thickness of 18 μm and manual cell counts were performed on the most medial coronal sections using ImageJ (cell counter) and analyzed as described above. Mice of either sex were used for all experiments unless otherwise stated. For behavioral experiments, manual cell counts were conducted on confocal or bright-field sections to assess pS6 immunoreactivity in the VNO.

### Image analysis using Imaris

Confocal image stacks were imported into Imaris (Bitplane) for 3D rendering, mask creation and fluorescence quantification. Regions of interest were defined by creating surface or volume masks based on a reference marker channel (e.g. ER marker or cell-identity marker), using standardized intensity thresholds and smoothing parameters applied consistently across samples. The fluorescence signal of the target channel was then through the Imaris MeasurementPro module yielding the sum of fluorescent intensity and volume for each 3D masked region. To account for variability in mask size, fluorescence values were normalized to mask volume. All parameters (thresholds, filter values and mask creation steps) were kept constant for wild-type and experimental groups. Data were exported to Excel and then Prism for statistical analysis.

## Supplementary Material



10.1242/develop.205386_sup1 Supplementary information
