# Supplementary material for: Loss of the endoplasmic reticulum protein canopy 1 disrupts the function and circuit organization of V2R-expressing vomeronasal sensory neurons
Source: Development. 2026 Jun 22;153(12):dev205386. doi: 10.1242/dev.205386 (PMC13354959; doi:10.1242/dev.205386)
Supplement: Supplementary information [file develop-153-205386-s1.pdf]

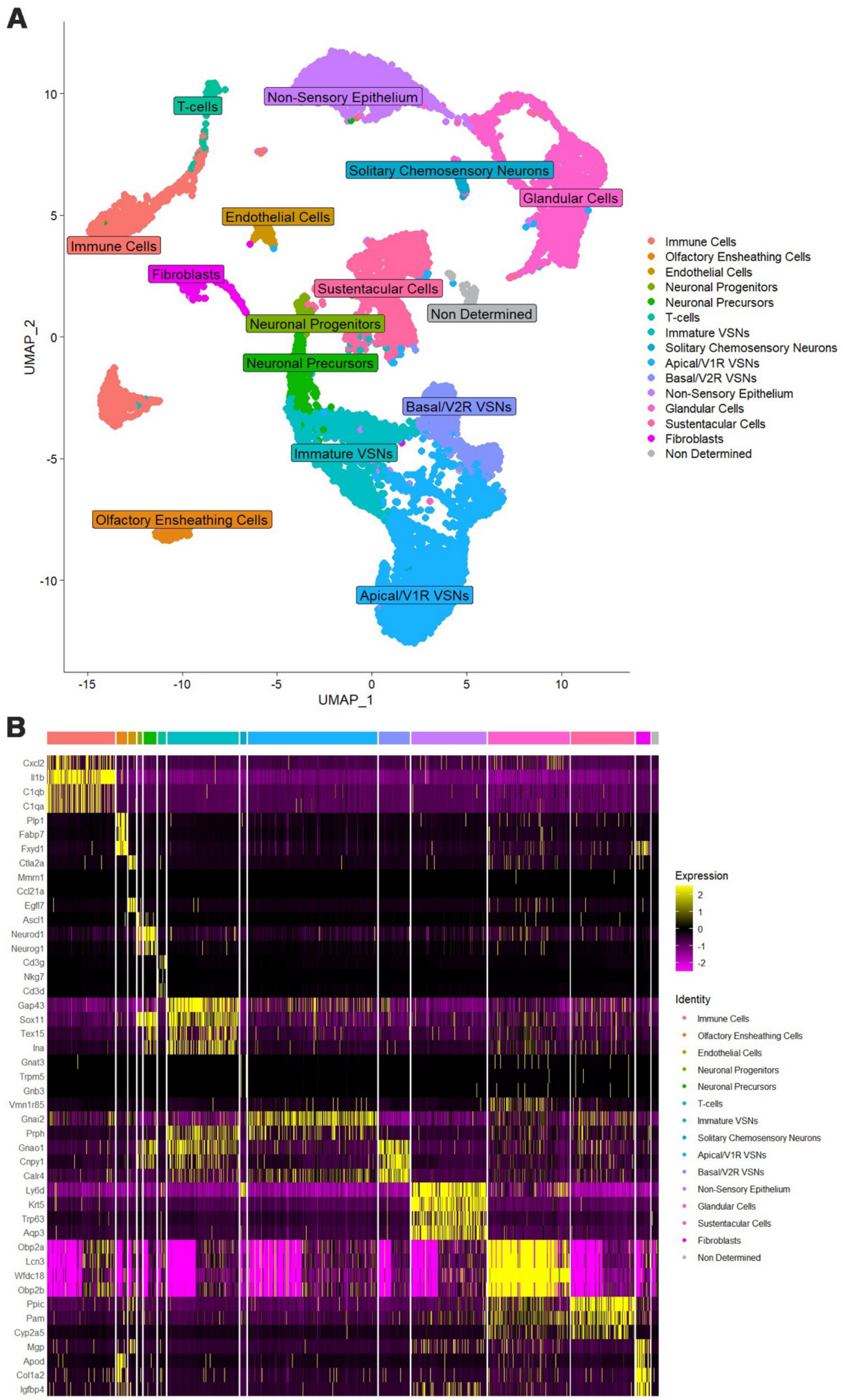

Fig. S1. Neuronal and Non-Neuronal Cell Type Clustering of the Whole Vomeronasal Organ. (A) P21 WT and KO single cell sequencing of the vomeronasal organ was used to generate a UMAP dimensional reduction plot. Each individually colored and labeled cluster identifies a different cell type in the VNO that shares a similar transcriptomic profile identified based on known associated gene expression. (B) Heatmap of WT and KO single cell sequencing showing the top genes expressed in each labeled cluster of the whole VNO on the Y-axis and each color associated with the labeled UMAP clusters on the top X-axis.

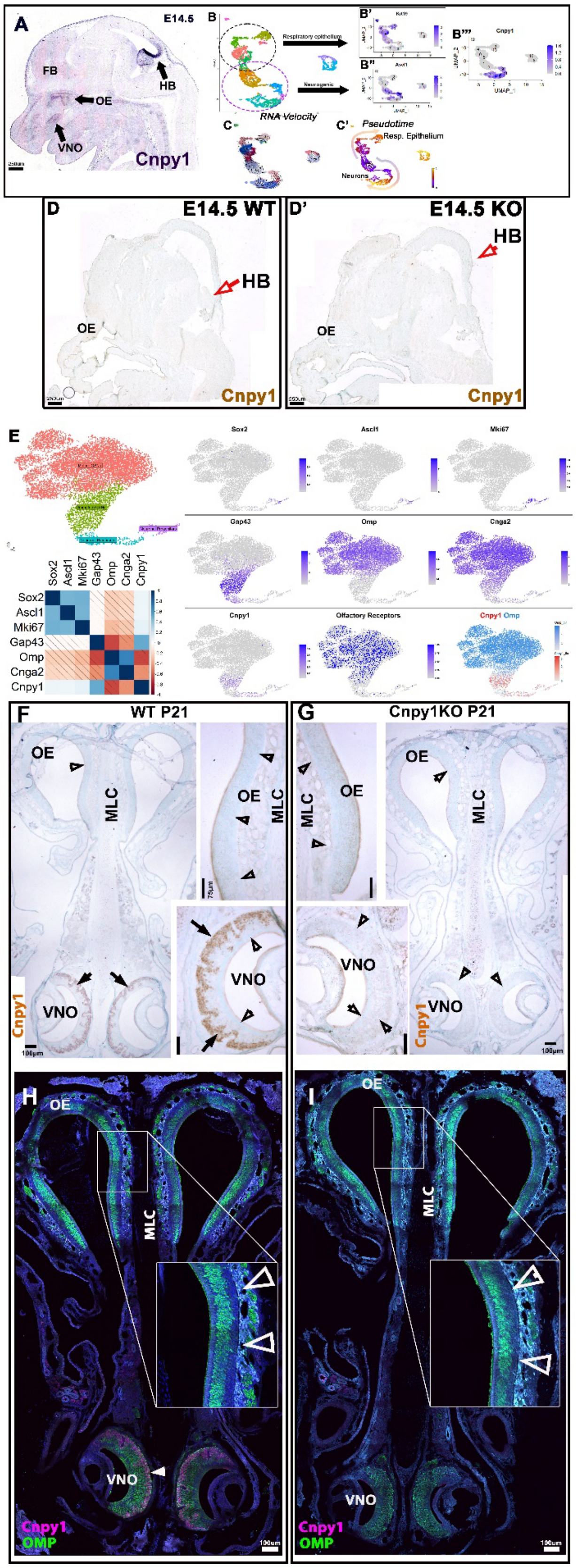
A
E14.5
FB
OE
HB
VNO
Cnpy1
B
Respiratory epithelium
Neurogenic
RNA Velocity
B'
B''
B'''
Cnpy1
C
C'
Pseudotime
Resp. Epithelium
Neurons
D
E14.5 WT
HB
OE
Cnpy1
D'
E14.5 KO
HB
OE
Cnpy1
E
Sox2
Ascl1
Mki67
Gap43
Omp
Cnga2
Cnpy1
Olfactory Receptors
Cnpy1 Omp
F
WT P21
OE
MLC
VNO
Cnpy1
100µm
75µm
G
Cnpy1KO P21
OE
MLC
VNO
Cnpy1
100µm
H
OE
MLC
VNO
Cnpy1
OMP
100um
I
OE
MLC
VNO
Cnpy1
OMP
100um

Fig. S2. Cnpy1 translation is limited to the VNO

(A) In situ hybridization for Cnpy1 on E14.5 embryonic heads shows expression in the vomeronasal organ (VNO), olfactory epithelium (OE), and mid-hindbrain boundary (HB). No signal in the forebrain (FB). (B-B''') Single-cell RNA-seq of the E14 nasal region identifies major cell types via UMAP clustering, with expression of epithelial (Krt19), respiratory (Ascl1), and Cnpy1 transcripts in developing chemosensory neurons. (C-C') RNA velocity and pseudotime analyses showing differentiation from progenitors to mature neurons, with Cnpy1 cells along the neurogenic pathway. (D-D') Immunohistochemistry at E14.5 in WT and Cnpy1 KO embryos shows no detectable protein, with arrows indicating the developing hindbrain. (E) Single-cell RNA-seq of postnatal main olfactory epithelium (MOE) shows expression of markers (Sox2, Ascl1, Mki67, Gap43, Omp, Cnga2) and Cnpy1; gene-gene Pearson correlations indicate limited association of Cnpy1 with mature OSNs. (F-G) Immunohistochemistry at P21 reveals Cnpy1 in VNO but not in OE or Midline Cartilage (MLC), with higher magnification confirming restriction to VNO (arrows). (H-I) Immunofluorescence shows Cnpy1 in VNO neurons (arrows) but absent in OMP-positive OE neurons; in-sets display higher magnification of regions lacking Cnpy1 (Empty arrowheads). Scale bars as indicated.

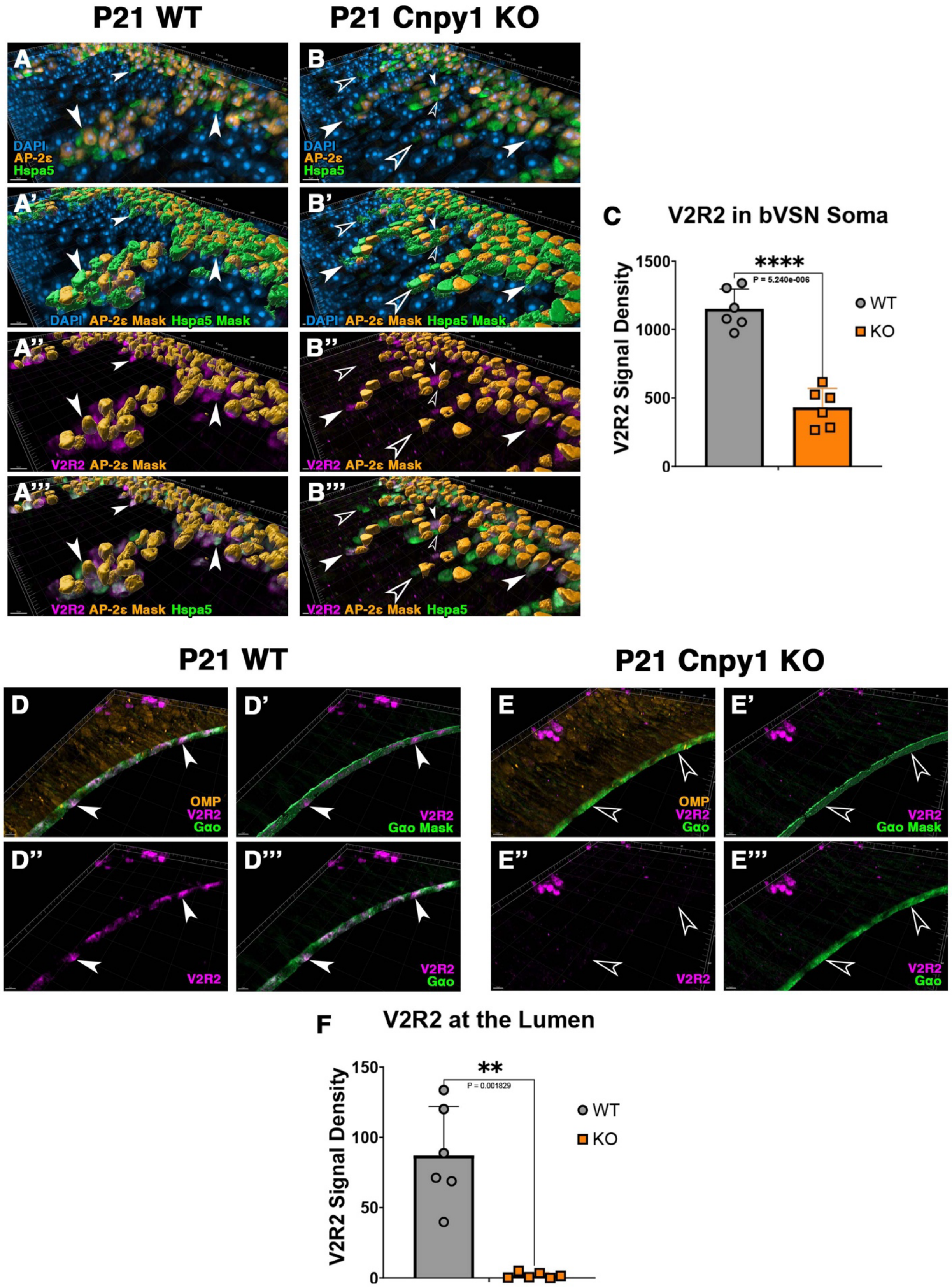

P21 WT
P21 Cnpy1 KO
A
DAPI
AP-2ε
Hspa5
B
A'
DAPI AP-2ε Mask Hspa5 Mask
B'
A''
V2R2 AP-2ε Mask
B''
A'''
V2R2 AP-2ε Mask Hspa5
B'''
C
V2R2 in bVSN Soma
V2R2 Signal Density
****
P = 5.240e-006
WT
KO
1500
1000
500
0
P21 WT
P21 Cnpy1 KO
D
OMP
V2R2
Gαo
D'
V2R2
Gαo Mask
D''
V2R2
D'''
E
E'
E''
E'''
F
V2R2 at the Lumen
**
P = 0.001829
150
100
50
0

Fig. S3. 3D reconstruction of basal VSN ER and luminal surface reveals reduced V2R2 protein in Cnpy1 KO. (A-B) P21 WT and Cnpy1 KO VNO sections stained for V2R2, HSPA5 (ER), AP-2ε, and DAPI; HSPA5 defines basal VSN ER. (A'-B') 3D reconstruction generates ER masks used to quantify V2R2 localization. (A''-B'') ER masks applied to V2R2 signal enable volumetric quantification within ER regions. (A'''-B''') V2R2-ER colocalization shown in white; solid arrows mark V2R2-positive ER, open arrows indicate absence. (C) V2R2 density within ER is significantly reduced in KO (****P = 5.24E-6). (D-E) 3D reconstruction of the luminal surface using Gαo reveals reduced V2R2 signal in KO. (F) Luminal V2R2 density is decreased in KO (**P = 0.0018). Data are mean ± SEM, N≥3, Welch's unpaired t-test.

Development • Supplementary information

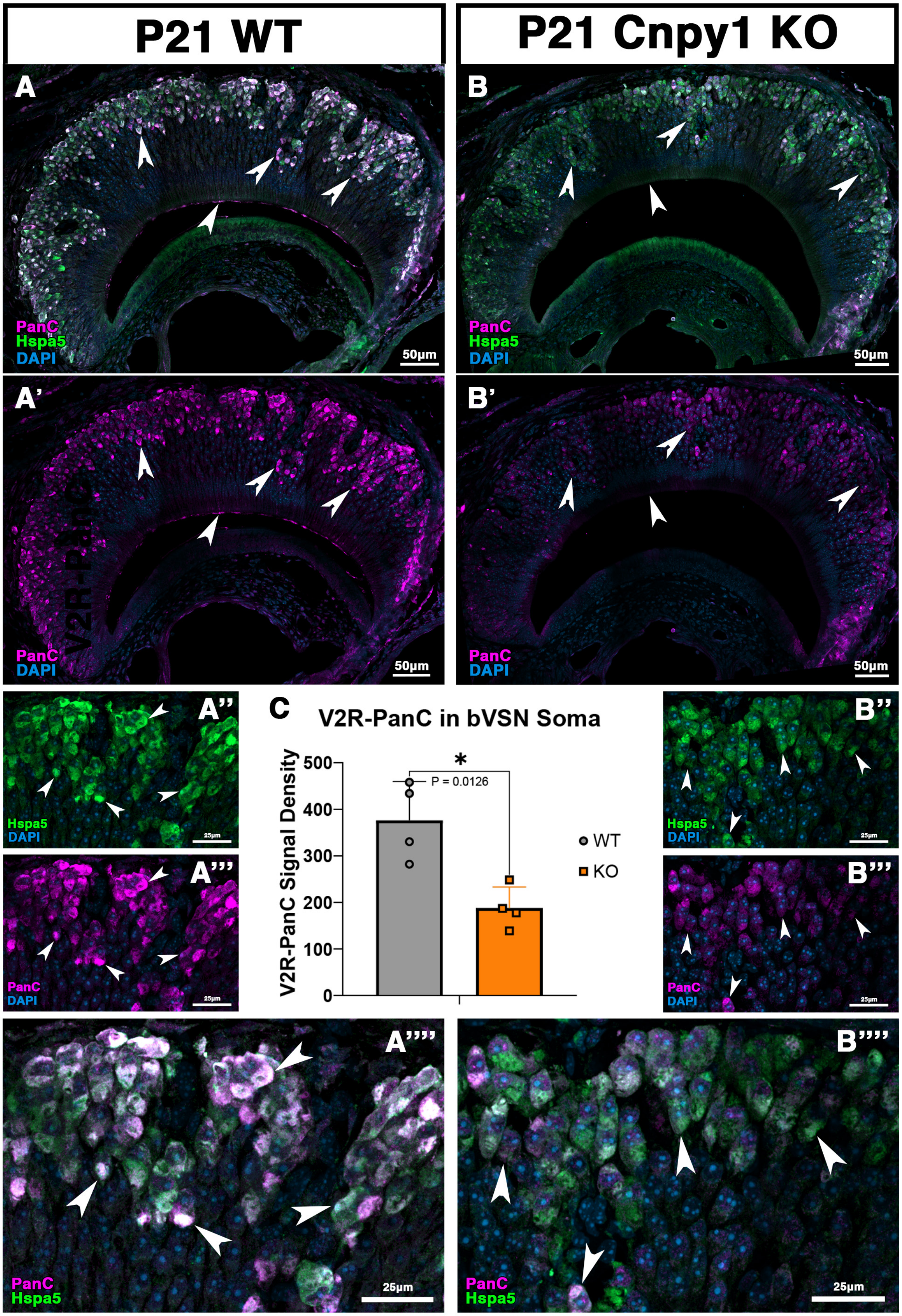

Development • Supplementary information

Fig. S4. Cnpy1 loss significantly alters V2R Family-C protein levels in basal VSNs at P21. P21 WT (A) and Cnpy1 KO (B) VNO sections stained for V2R-PanC (magenta), HSPA5 (green), and DAPI (blue). In WT, robust V2R-PanC signal is observed throughout basal VSN soma and extending toward dendritic compartments (arrowheads). In Cnpy1 KO, V2R-PanC signal is visibly reduced in basal neurons, particularly at the lumen. (A'-B') V2R-PanC and DAPI staining highlights a marked reduction of V2R immunoreactivity in KO (arrowheads). (A''-B''') Higher magnification views of basal VSN soma show co-localization of V2R-PanC with HSPA5 in WT, consistent with ER-associated receptor localization. In Cnpy1 KO, V2R-PanC signal is reduced within HSPA5-positive ER regions. (A''''-B'''') High-magnification images of basal VSN soma further illustrate decreased V2R-PanC immunofluorescence in KO. (C) Quantification of V2R-PanC signal density shows reduction in Cnpy1 KO ($P = 0.0126$). Each point represents one VNO ($N \geq 3$); data are shown as mean ± SEM. Statistical significance determined by Welch's unpaired two-tailed t-test. Scale bars: 50 μm (A-B'), 25 μm (A''-B'''').

Development • Supplementary information

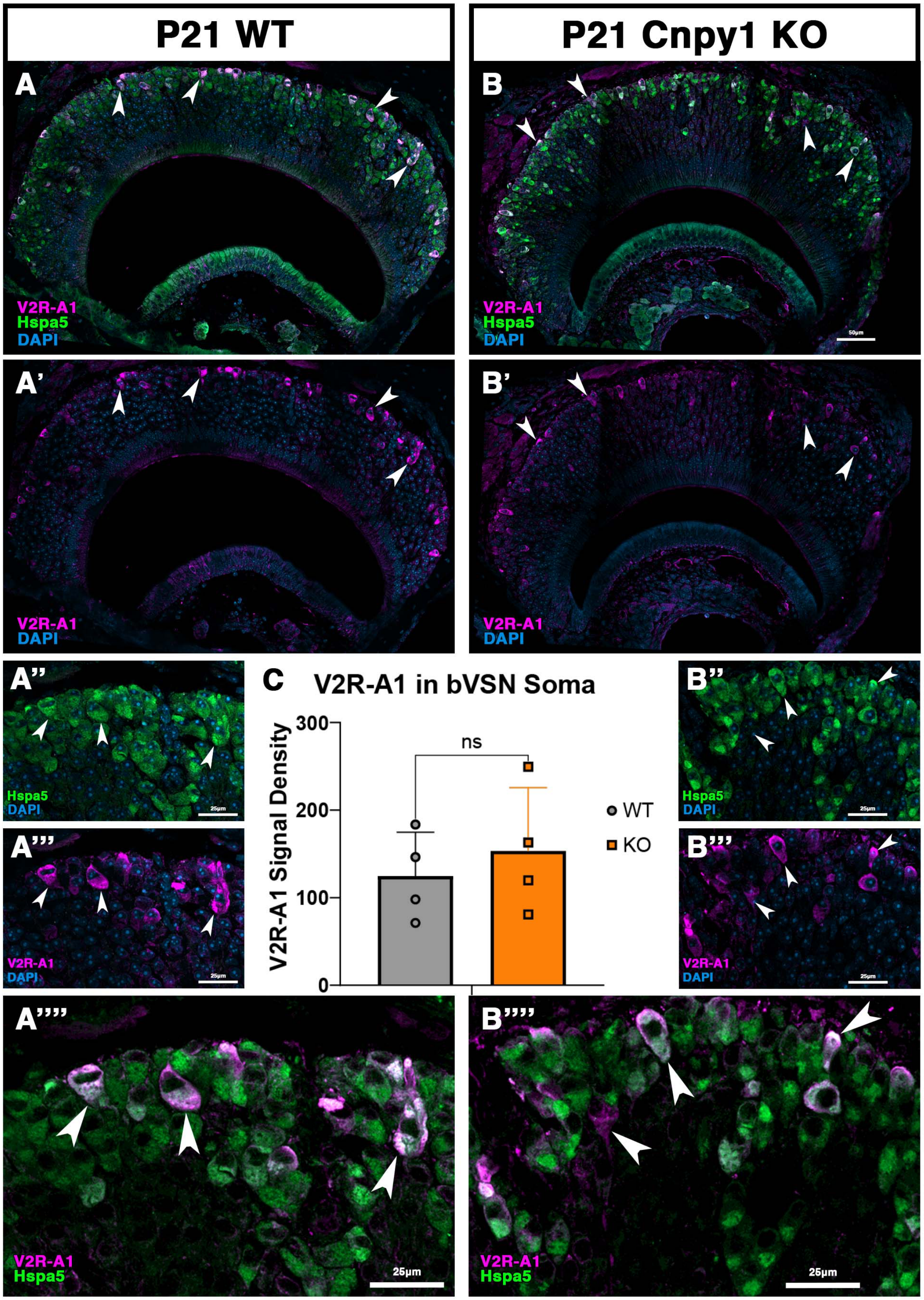

Development • Supplementary information

Fig. S5. Cnpy1 loss does not significantly alter V2R-A1 protein levels in basal VSNs at P21. P21 WT (A) and Cnpy1 KO (B) VNO sections stained for V2R-A1 (magenta), HSPA5 (green; ER marker), and DAPI (blue). V2R-A1-positive basal VSNs are distributed similarly across genotypes (arrowheads). (A'-B') V2R-A1 and DAPI staining shows comparable overall signal intensity and spatial distribution in WT and KO. (A''-B''') Higher magnification views reveal V2R-A1 localization within HSPA5-positive ER regions in both WT and KO basal VSN soma. (A''''B'''') High-magnification images further confirm similar V2R-A1 immunoreactivity between genotypes. (C) Quantification of V2R-A1 signal density in basal VSN soma shows no significant difference between WT and Cnpy1 KO (ns). Each dot represents one VNO (N=3-4); mean ± SEM; Welch's unpaired two-tailed t-test. Scale bars: 50 μm (A-B'), 25 μm (A''-B'''').

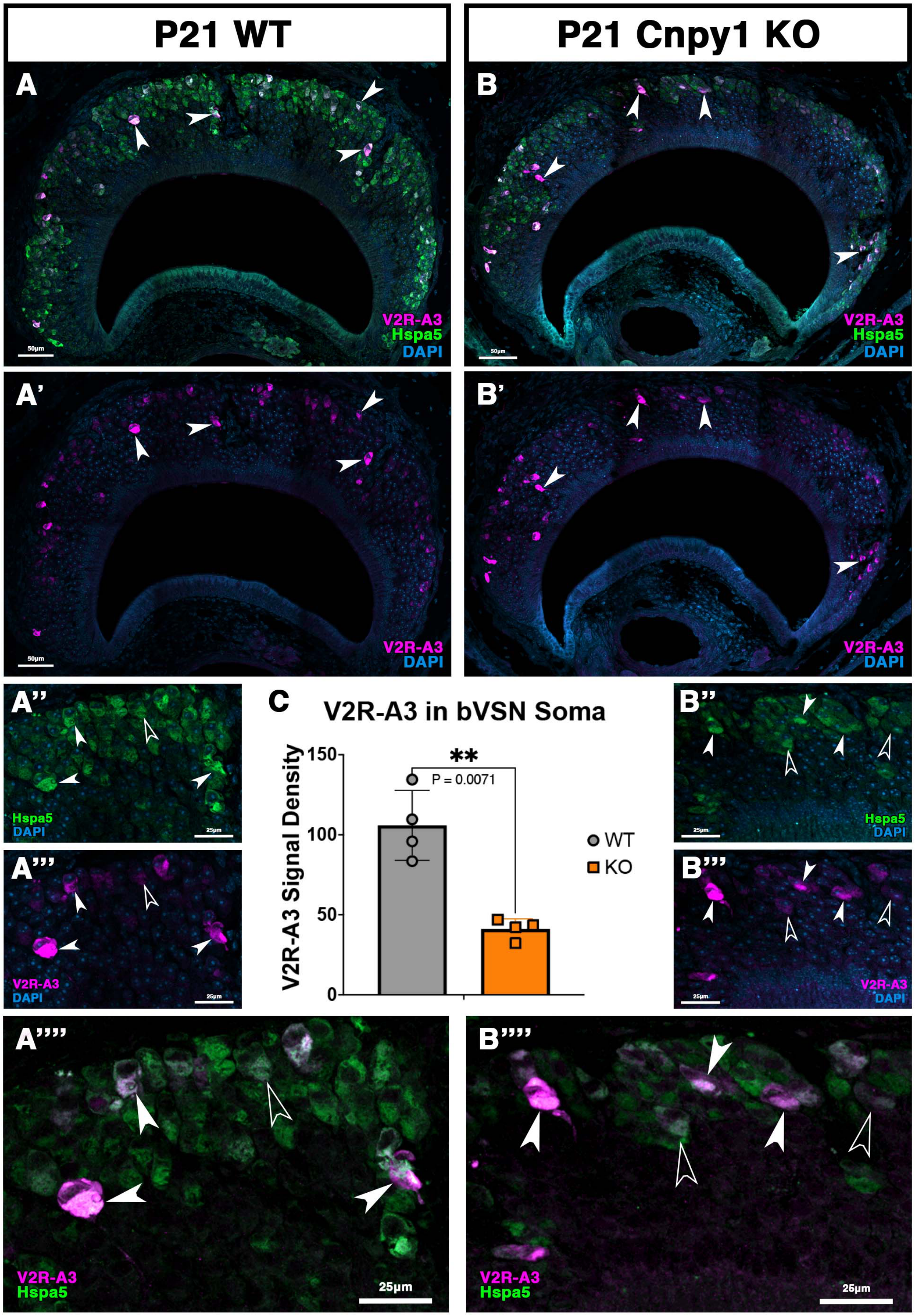
P21 WT
P21 Cnpy1 KO
A
B
V2R-A3
Hspa5
DAPI
50µm
A'
B'
A''
B''
A'''
B'''
C
V2R-A3 in bVSN Soma
V2R-A3 Signal Density
150
100
50
0
**
P = 0.0071
WT
KO
A''''
B''''
25µm

Fig. S6. Cnpy1 loss reduces V2R-A3 protein levels in basal VSNs at P21. (A-B) Coronal sections of P21 WT (A) and Cnpy1 KO (B) VNO stained for V2R-A3 (magenta), HSPA5 (green; ER marker), and DAPI (blue). Arrowheads indicate V2R-A3-positive basal VSNs, empty arrows indicate weak staining. (A'-B') V2R-A3 and DAPI staining shows reduced V2R-A3 signal in KO compared to WT. (A''-B''') Higher magnifications show V2R-A3 localization within HSPA5-positive ER regions in WT and KO. (A''''-B'''') High-magnification views of basal VSN soma further illustrate decreased V2R-A3 immunoreactivity in KO. C) Quantification of V2R-A3 signal density in basal VSN soma shows reduction in Cnpy1 KO P = 0.0071. Each dot represents one VNO (N>=3); mean ± SEM; Welch's unpaired two-tailed t-test. Scale bars: 50 µm (A-B'), 25 µm (A''-B'''').

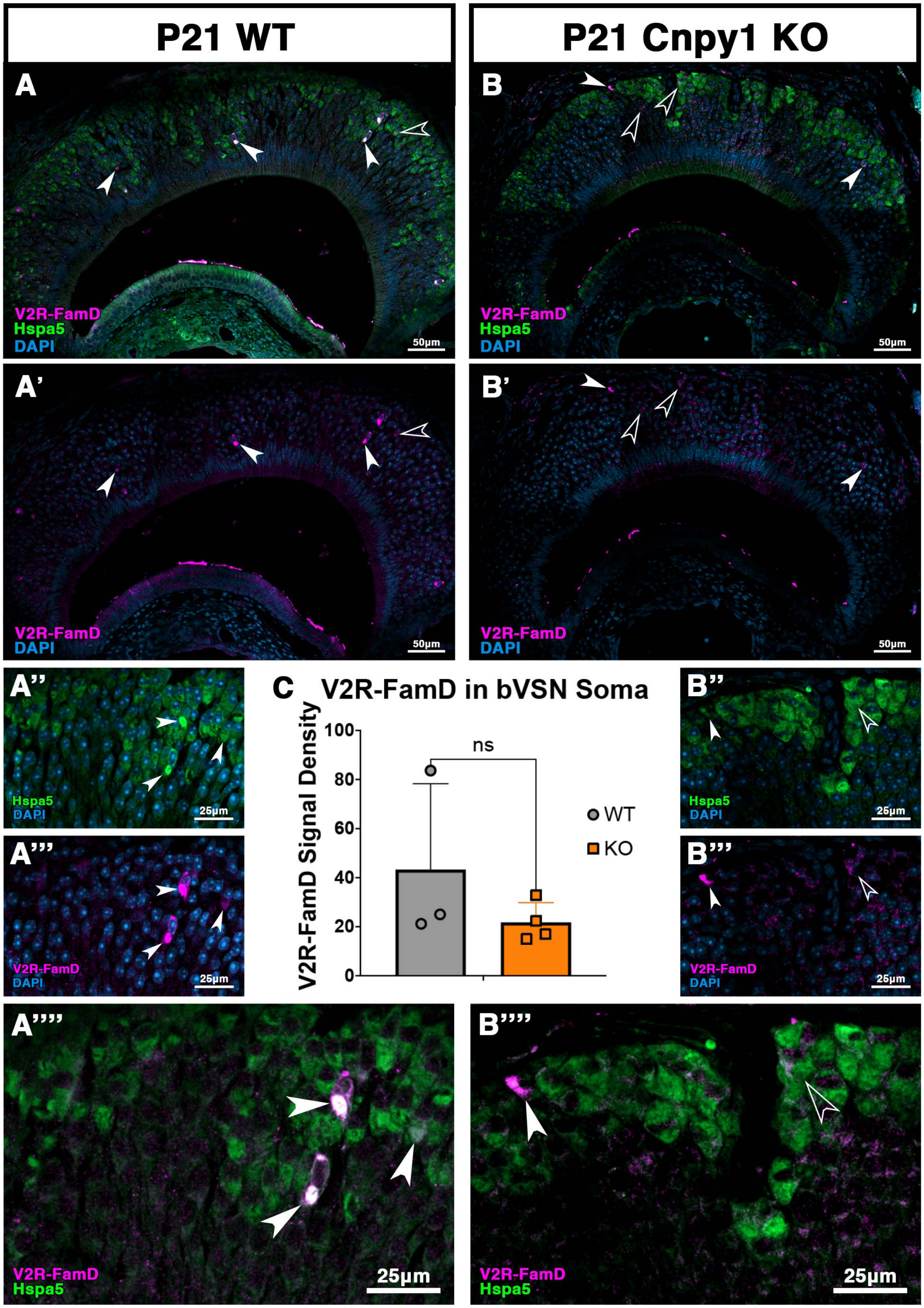
P21 WT
P21 Cnpy1 KO
A
V2R-FamD
Hspa5
DAPI
50µm
B
A'
B'
A''
A'''
A''''
B''
B'''
B''''
25µm
C
V2R-FamD in bVSN Soma
V2R-FamD Signal Density
ns
WT
KO
0
20
40
60
80
100

Fig. S7. Cnpy1 loss reduces V2R-FamD protein levels in basal VSNs at P21. ((A-B) Coronal sections of P21 WT (A) and Cnpy1 KO (B) VNO stained for V2R-FamD (magenta), HSPA5 (green; ER marker), and DAPI (blue). Arrowheads indicate V2R-FamD-positive basal VSNs where empty arrowheads indicate weak or missing V2R-FamD staining (A'-B') V2R-FamD and DAPI staining shows reduced V2R-FamD signal in KO compared to WT. (A''-B''') Higher magnifications show V2R-FamD localization within HSPA5-positive ER regions in WT and KO. (A''''-B'''') Further magnified views of basal VSN soma further illustrate decreased V2R-FamD immunoreactivity in KO. C). Each point represents one VNO (N>=3); mean ± SEM; Welch's unpaired two-tailed t-test. Scale bars: 50 μm (A-B'), 25 μm (A''-B'''').

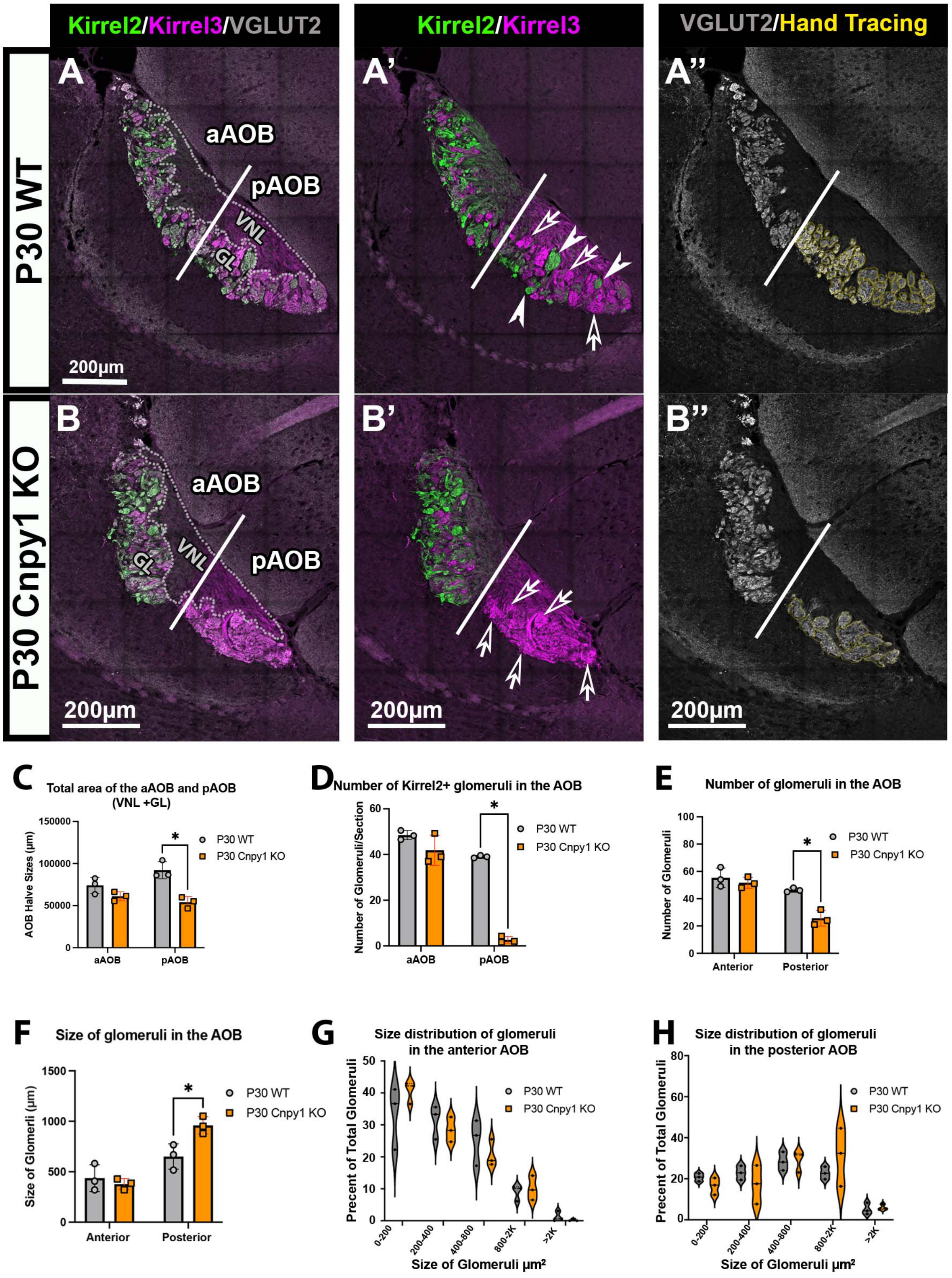
Kirrel2/Kirrel3/VGLUT2
Kirrel2/Kirrel3
VGLUT2/Hand Tracing
P30 WT
P30 Cnpy1 KO
A
A'
A''
B
B'
B''
aAOB
pAOB
VNL
GL
200µm
C
Total area of the aAOB and pAOB (VNL +GL)
AOB Halve Sizes (µm)
P30 WT
P30 Cnpy1 KO
aAOB
pAOB
D
Number of Kirrel2+ glomeruli in the AOB
Number of Glomeruli/Section
E
Number of glomeruli in the AOB
Number of Glomeruli
Anterior
Posterior
F
Size of glomeruli in the AOB
Size of Glomerli (µm)
G
Size distribution of glomeruli in the anterior AOB
Precent of Total Glomeruli
Size of Glomeruli µm²
0-200
200-400
400-800
800-2K
>2K
H
Size distribution of glomeruli in the posterior AOB

Fig. S8. Loss of CNPY1 disrupts adhesion molecule expression and alters posterior AOB organization and glomerular architecture. (A-A'') P30 WT AOB sections stained for Kirrel2, Kirrel3, and VGLUT2 show normal segregation of anterior (aAOB) and posterior (pAOB) glomeruli. (B-B'') Cnpy1KO sections display reduced pAOB territory and disrupted Kirrel2+/Kirrel3+ glomerular patterning, indicating altered anterior-posterior organization. (A'', B'') VGLUT2 labeling with hand tracing highlights glomerular distribution in WT and KO. (C) Total AOB area (VNL + GL) is reduced in KO pAOB ($p < 0.005581$), with no change in aAOB. (D) Kirrel2+/VGLUT2+ glomeruli are decreased in KO pAOB ($p < 0.000002$), unchanged in aAOB. (E) Total VGLUT2+ glomeruli are reduced in KO (aAOB $p < 0.013294$; pAOB $p < 0.002169$). (F) Glomerular size is increased in KO pAOB ($p < 0.000463$), with no change in aAOB. (G-H) Size distribution shows no selective loss of specific glomerular classes. Data are mean ± SEM, N = 3, unpaired t-tests. Scale bars: 200 μm.
