# Supplementary material for: Loss of the endoplasmic reticulum protein canopy 1 disrupts the function and circuit organization of V2R-expressing vomeronasal sensory neurons
Source: Development. 2026 Jun 22;153(12):dev205386. doi: 10.1242/dev.205386 (PMC13354959; doi:10.1242/dev.205386)
Supplement: Supplementary file 1 [file develop-153-205386_review_history.pdf]

Development

Loss of the endoplasmic reticulum protein canopy 1 disrupts the function and circuit organization of V2R-expressing vomeronasal sensory neurons

Nicholas Mathias, Nikki Dolphin, Noah M. LeFever, Enrico Amato, Carlsy S. Ybanez, Anthony Ruquet and Paolo E. Forni

DOI: 10.1242/dev.205386

Editor: James Briscoe

Review timeline

Original submission:	19 November 2025
Editorial decision:	24 December 2025
First revision received:	22 April 2026
Editorial decision:	13 May 2026
Second revision received:	19 May 2026
Accepted:	21 May 2026

Original submission

First decision letter

MS ID#: dev.205386

MS TITLE: Canopy1/Cnpy1 is required for proper V2R processing and transport; its loss impairs the function and circuit organization of basal/V2R vomeronasal sensory neurons.

AUTHORS: Nicholas Mathias; Nikki Dolphin; Anthony Ruquet; Paolo E. Forni

Dear Dr Forni,

I have now received all the referees' reports on the above manuscript, and have reached a decision. The referees' comments are appended below.

As you will see, the referees express considerable interest in your work, but have some significant criticisms and recommend a substantial revision of your manuscript before we can consider publication. The referees are concerned about how generalisable the V2R2 findings will be. Examining typical V2Rs would strengthen the case. Please clarify the mechanism of basal VSN loss by quantifying apoptosis within the basal layer and at earlier postnatal stages to reconcile discrepancies with the Devakinandan study, and assessing cell proliferation to distinguish between cell death and impaired neurogenesis. The referees also request strengthening of the chemosensory activation data and the analysis of the pS6 data.

If you are able to revise the manuscript along the lines the referees suggest, which may involve further experiments, I will be happy to receive a revised version of the manuscript. Your revised paper will be re-reviewed by one or more of the original referees, and acceptance of your manuscript will depend on your addressing satisfactorily the reviewers' major concerns. Please also note that Development will normally permit only one round of major revision. If it would be helpful, you are welcome to contact us to discuss your revision in greater detail. Please send us a point-by-point response indicating your plans for addressing the referees' comments, and we will look over this and provide further guidance.

© 2026. Published by The Company of Biologists under the terms of the Creative Commons Attribution License (https://creativecommons.org/licenses/by/4.0/).

Please attend to all of the reviewers' comments and ensure that you upload both a 'clean' version of your Word file, along with a highlighted version clearly showing where you have made changes in the revised manuscript. Please avoid using 'Tracked changes' in Word files as these are lost in PDF conversion. I should be grateful if you would also provide a point-by-point response detailing how you have dealt with the points raised by the reviewers in the 'Response to Reviewers' box. If you do not agree with any of their criticisms or suggestions please explain clearly why this is so.

Reviewer 1

The manuscript by Nicholas Mathias et al. examines the anatomical organization of the vomeronasal systems in a Cnpy1 KO mouse during postnatal stages. The authors report progressive degeneration and loss of basal VNO sensory neurons, as well as disrupted connectivity to the AOB, by using mainly immunolabeling methods. With some exceptions, the conclusions are generally well supported by the data. The manuscript can be improved with some revisions as suggested below.

Fig1: UMAP representation of VNO cells do not contain non-neuronal cell types such as sustentacular, glial, and other cell types. Please, indicate whether datasets were filtered for neuronal and stem cell lineages only in the Results and methods sections.

Transcriptomic analysis: no data regarding specific V2R expression (apart from Vmn2r2) is reported. In addition, the V2R receptor expression section (fig 7) generalizes about expression levels and fluorescence signal, when this can be only applied to the 1-2 receptors detected by the V2R2 antibody, which incidentally are expressed by most basal VSNs. Are there any other V2Rs among the DE genes? is cell reduction affecting a particular V2R-neuron subpopulation other than V2R2 (based on the RNAseq data)? what about H2-Mv genes and b2m-globulin, calretinin and other reported chaperones?

Chemo-sensory activity analysis (Fig. 6): the pS6 immunolabeling is not very convincing, particularly in WT samples which show too many positive cells. It seems to me that background signal is too high. To have an idea of the basal level of pS6 activity, it is necessary to include a non-stimulated control. In addition, monitoring of c-Fos activity in the AOB of the same animals would strongly support the conclusion.

Fig. 8: The authors suggest that V2Rs do not reach lumen of the VNO, probably because defective trafficking and expression to the plasma membrane in the VSN dendrites/microvilli. To better support this conclusion, please show evidence reporting intact dendrite/microvillar integrity of basal VSNs in the KO mouse.

Fig. 10: the observed increase in glomerular size is somehow puzzling. Are glomeruli indeed larger? Or the observation is rather a decrease in small glomeruli?

Fig. 11C: Using immunofluorescence density signal as a proxy for Cnpy1 expression is not a reliable method, and can be subject to bias derived from technical variability. Other quantitative, more precise methods such as qPCR would be more appropriate. Otherwise, the conclusion that "we observe reduced Cnpy1 expression" (line 367) should be removed. Alternatively, this piece of data is only tangential to the study and can be skipped, as it does not provide much new information.

Discussion: the authors emphasize the lack of studies involving Cnpy1 in mammals. It would be advisable to provide some context information about canopy expression in other cell types outside of the VNO, and potential interaction with other receptors different from V2Rs (perhaps other class C GPCRs) and their trafficking to the plasma membrane. This will provide key information about the specificity of Cnpy1 expression and function in the VNO.

Minor:
- Abstract (and whole text): symbols of specific proteins (alpha, epsilon, gamma…) are sometimes typed in the Latin forms. Please, stick to one specific alphabet (preferably Greek) for consistency.
- Line 60: remove "CUT&RUN" from the text
- line 124: no need to report that the Cnpy1 knockout mouse is "unpublished"

© 2026. Published by The Company of Biologists under the terms of the Creative Commons Attribution License (https://creativecommons.org/licenses/by/4.0/).

- line 191: specify which "lumen" (VNO lumen)
-line 338: "… that ER stress physiologically increases…" better indicate ER stress-related genes
-Fig. 6C: graph data should be expressed in real units (VSNs/section or similar)

Reviewer 2

SUMMARY OF THE ADVANCE MADE IN THIS PAPER AND ITS POTENTIAL SIGNIFICANCE TO THE FIELD

This manuscript by Mathias et al. provides a thorough investigation into the role of the ER protein Canopy1 (Cnpy1) in the development and maintenance of basal vomeronasal sensory neurons (VSNs). The authors convincingly demonstrate that Cnpy1 expression is restricted to differentiating/maturing basal VSNs and that its ablation results in progressive loss of these neurons postnatally, accompanied by reduced V2R expression and mislocalization, ultimately impairing neuronal activation in response to urine chemosignals.
The manuscript is generally well written, and the data are compelling. It is worth noting that the authors acknowledge a recent study by Devakinandan et al. (bioRxiv, 2025) that also addresses Cnpy1 function in basal VSNs. While both studies confirm a role for Cnpy1, they provide complementary insights. Mathias et al. uniquely contribute by characterizing cell surface and axon guidance receptors in basal VSNs, identifying glomerular defects in Cnpy1 mutants, and demonstrating that Cnpy1 expression is reduced in AP2E mutants, implicating AP2E in transcriptional regulation of Cnpy1.
Overall, this is a strong manuscript that makes a valuable contribution to the field. The findings are significant and will be of great interest to developmental biologists studying sensory neuron differentiation and fate specification.

SUGGESTIONS TO AUTHORS

Specific comments:
1. There are two important discrepancies between the results presented by the authors and Devakinandan et al. (cell death and V2R localization in Cnpy1 mutants), which should be explicitly discussed, and for which potential explanations be offered to help readers reconcile the differences.
2. As Devakinandan et al. reported increased apoptosis in the VNO of Cnpy1 mutants at earlier postnatal stages than the one assessed by the authors, they should perform cleaved caspase-3 (CC3) staining at earlier time points to determine whether transient apoptotic events were missed in their analysis.
3. The authors propose that reduced Kirrel2 expression contributes to posterior AOB glomerular defects in Cnpy1 mutants. Since glomerular formation occurs during early postnatal development, it would be important to assess Kirrel2 expression in basal VSNs at earlier stages to strengthen this conclusion.

Minor comments:

1. The increased Vmn2R2 mRNA expression in Cnpy1 KO animals is shown in Figure 7, and mentioned in the discussion, but should also be addressed in the text of the results section.

2. As loss of Cnpy1 leads to reduced Kirrel2 expression, but not Kirrel3, in basal VSNs, the authors should consider referencing the paper by Brignall et al. in 2018, which reports similar glomerular defects to Cnpy1 when Kirrel2 is ablated from VSNs.

3. On Line 115, in the sentence: "However, its mRNA expression rapidly declines in differentiating/maturing (Meis2+; Gai2/Gnai2) V1R VSNs while remaining active and highly correlated with the expression of genes belonging to the V2R basal lineage (Fig. 1A, 2A).
It is unclear what is "remaining active". Do they mean "while remaining expressed"?

4.Line 236: "Quantification of glomeruli number and size showed an overall reduction of the posterior AOB (Fig. 10G), which, like for other mutants, reflects a reduction of basal VSNs." It is unclear which mutants are being referred to in this sentence.

© 2026. Published by The Company of Biologists under the terms of the Creative Commons Attribution License (https://creativecommons.org/licenses/by/4.0/).

5.Line 293 " Cnpy1 is the among the most highly expressed genes " should be "is among the..."

6.In Figure 4, it is unclear how the average number of VSNs/section can be lower in VNOS from P60 mice than VNOs from P21 mice considering that the VNO should be larger in older mice.

7.P values are missing on graphs in Figure 10 8.For Figure 8, p value on graph is p=0.0001, while it is reported as p<0.0001 in the figure legend.

9.The Figure legends should be reviewed to ensure consistency of the information provided across the legends.

Reviewer 3

SUMMARY OF THE ADVANCE MADE IN THIS PAPER AND ITS POTENTIAL SIGNIFICANCE TO THE FIELD

In this manuscript, Mathias et al. investigate the functional role of the ER-associated protein Canopy 1 (Cnpy1), which is one of the genes differentially expressed between V1R- and V2R-expressing VSNs. In the V2R-expressing VSN lineage, Cnpy1 expression is maintained from globose basal progenitors through to mature VSNs, whereas its expression rapidly declines during differentiation of the V1R-expressing VSN lineage.

The authors performed histological and transcriptomic analyses of Cnpy1 knockout (KO) mice and demonstrate that loss of Cnpy1 results in a reduction in the number of V2R-VSNs, altered expression of ER stress-responsive genes and axon guidance molecules, and disorganization of the posterior accessory olfactory bulb (AOB). Upon exposure to male urine, the number of activated VSNs is significantly reduced specifically in V2R-VSNs of Cnpy1 KO males. Furthermore, immunohistochemical analysis of V2R2, an atypical V2R broadly expressed in V2R-VSNs, shows reduced V2R2 immunoreactivity in cell bodies and complete loss of signal in the dendritic lumen of the Cnpy1 KO vomeronasal organ. These results suggest that impaired transport of V2R2 protein to the dendritic lumen leads to reduced neuronal activity in V2R-VSNs.

Overall, this study demonstrates an important role for Cnpy1 in V2R processing and transport, which is essential for proper V2R-VSN function. The manuscript is technically sound and presents potentially important findings, with several conclusions supported by convincing data. However, I would like the authors to address several major issues, which are outlined below.

SUGGESTIONS TO AUTHORS

Major points

1) In this study, the authors performed immunohistochemical analyses of V2R2, a family C V2R. V2R2 is an atypical V2R that is broadly expressed in the basal layer of vomeronasal sensory neurons (VSNs) and is co-expressed with typical V2Rs (family ABD V2Rs). Previous work has shown that loss of Vmn2r1, a member of the family C V2Rs, leads to a reduction in the basal V2R-expressing VSN population (Akiyoshi et al., 2018; doi:10.1111/ejn.13875); however, the functional contribution of family C V2Rs to pheromone-evoked VSN responses remains unclear. In this context, it is essential that the authors also examine the expression of typical V2R proteins. Such analyses would help clarify the basis for the reduced responses of basal VSNs observed in the Cnpy1 knockout (Fig. 6) and would substantially strengthen the reliability of the conclusions and the overall significance of the manuscript.
In addition, the manuscript describes the results of the V2R2 analysis as though they are representative of typical V2Rs, which is not accurate. Conclusions based on V2R2 should be presented with appropriate caution and clearly framed as findings specific to V2R2 rather than generalized to typical V2Rs.

2) Line 147-152, Fig. 4: The mechanism underlying the reduction in basal vomeronasal sensory neurons (VSNs) in the Cnpy1 knockout remains unclear. Although the authors quantified apoptotic cells across the entire vomeronasal epithelium, the reported neuronal loss appears to be restricted

© 2026. Published by The Company of Biologists under the terms of the Creative Commons Attribution License (https://creativecommons.org/licenses/by/4.0/).

to the basal region. Therefore, apoptotic cell counts should be specifically quantified within the basal layer.
Moreover, the authors state that a "progressive neurodegenerative phenotype begins prior to maturation of the neuron" (Lines 162-163). To support this claim, it would be more informative to quantify active caspase-3-positive cells separately in the marginal zone and in the basal mature VSN region, as schematically illustrated in Fig. 12.
In addition, because the observed reduction in VSN numbers could also result from impaired or aberrant cell proliferation, the authors should quantify proliferative cells in the Cnpy1 knockout to more fully address the underlying cellular mechanisms.

3) It is difficult to clearly identify pS6-positive cells in the presented images. Several cells indicated by arrowheads exhibit signal intensities comparable to those observed in the sustentacular cell layer and are therefore likely to represent background staining rather than true pS6 signals. The staining protocol and/or image acquisition conditions should be optimized to obtain more reliable and quantitatively robust data.
In addition, because the number of V2R-expressing VSNs is reduced in the Cnpy1 knockout, pS6-positive cell counts should be presented in a normalized manner (e.g., relative to the total number of basal or apical VSNs), rather than as absolute values.
Furthermore, according to Silvotti et al. (2018; reference cited at line 602), pS6 signals are more prominently detected in mice stimulated with pheromones secreted by the opposite sex. Therefore, I recommend assessing VNO activation in female mice exposed to male urine.

4) Line 212-214: The authors report that endoplasmic reticulum (ER) responses are increased in mature VSNs; however, Fig. 9 appears to show altered expression of signaling molecules associated with ER responses rather than direct evidence of increased ER stress. It is unclear whether the expression changes of all genes described are strictly dependent on activation of the ER stress response.
To more directly support the claim of enhanced ER stress, I suggest assessing the extent to which canonical ER stress signaling pathways are activated. For example, Shayya et al. (2022; reference cited at line 596) quantified the unfolded protein response (UPR) by measuring changes in Atf5 mRNA translation regulated by PERK-mediated eIF2α phosphorylation. Similarly, Dey and Matsunami (reference cited at line 510) evaluated UPR activation based on Xbp1 mRNA splicing mediated by IRE1 activation. Incorporating comparable analyses would substantially strengthen the interpretation of the data.

5) Prince et al. (2013; reference cited at line 582) reported that the expression levels of Kirrel2 and Kirrel3 are activity dependent and suggested that the expression of these cell adhesion molecules in VSNs correlates with the specific V1R or V2R expressed. Given that Fig. 10 shows Kirrel3-expressing basal VSNs projecting to the posterior accessory olfactory bulb (AOB) in Cnpy1 knockout mice, it is possible that V2R-expressing neurons with high Kirrel3 expression are preferentially maintained in this genotype.
In addition, family ABD V2Rs can be subdivided into C1-type V2Rs, which are co-expressed with Vmn1r1, and C2-type V2Rs, which are co-expressed with Vmn2r2-7 (Silvotti et al., 2011; doi:10.1371/journal.pone.0024462). Loss of Vmn2r1 results in a significant reduction in the number of C1-type V2R-expressing VSNs (Akiyoshi et al., 2018; doi:10.1111/ejn.13875). Considering the reported impairment of V2R2 protein expression, it is therefore expected that specific basal VSN subpopulations are selectively reduced in the Cnpy1 knockout.
Because single-cell transcriptomic analysis of Cnpy1 knockout mice is presented in Fig. 5, the expression patterns of individual V1R and V2R genes could be further interrogated. It would be informative if the authors could identify which receptor-expressing VSN populations persist in the Cnpy1 knockout and discuss potential mechanisms underlying the selective survival of these V2R-expressing neurons.

6) Fig. 13: The authors should more clearly articulate the functional role of Cnpy1 as revealed by this study. One plausible interpretation is that Cnpy1, together with other ER-associated proteins, is induced downstream of AP-2ε-regulated effector gene activation, and that these ER proteins are required for proper processing and trafficking of V2R2 proteins (and potentially typical V2Rs, which should be examined by immunohistochemistry as noted in Comment #1). Impaired processing of these receptors may, in turn, influence the expression of cell adhesion molecules, thereby affecting the organization of glomeruli in the accessory olfactory bulb (AOB).

© 2026. Published by The Company of Biologists under the terms of the Creative Commons Attribution License (https://creativecommons.org/licenses/by/4.0/).

Minor points

7) Line 25-26: In the abstract, the authors state "distinct neuronal types depend on unique ER protein repertoires to maintain proper proteostasis". I think this statement holds true only if the same phenomena are obtained in V1R-expressingVSNs. I suggest rewording this sentence.

8) Line 41-42: Four references about FPRs are cited, and references describe V1Rs and V2Rs should be also cited here.

9) Fig 2A: Do red and blue colors indicate the correlation coefficients, and what do the stripes indicate?

10) Fig 4A, B, D, E: Please mention arrowheads indicate in the legend.

11) Related Fig 4C, 4F: Please describe the quantification methods in detail, thickness of section, numbers of quantified sections, and sex of animals is missing in the Materials and Methods.

12) Line 156: "red and blue" is different from Fig 5A legend.

13) Family C V2R includes Vmn2r1-7, and which V2R does the anti-V2R2 antibody detect? In Fig. 7D, is the expression of all family C V2R mRNA increased in Cnpy1 KO?

14) Arrowheads in Fig.8 are referred to as "arrows" in the legend and line 195.

15) Line 205-206: "we observed markedly reduced immunoreactivity for V2R receptors, consistent with defects in receptor processing and trafficking." In this sentence, "suggesting" is more appropriate than "consistent with ".

16) In Fig. 10C and C', "Cnpy1-/-" should be "Cnpy1 KO".

17) In line 264-265, the name of strain "Neurogenin1-CreERt2" is referred to as "Neurog1-cre/ERT2" in the Materials and Methods, "Ngn1-Cre" in Fig. 12, and "Ngn1-CreERT2" in the Fig. 12 legend; consistency is required.

18) Line 217: Atf5 should be Atf6? In addition, Expression levels of Atf5 decrease in Cnpy1 KO, opposite to other genes. Please mention Atf5.

19) Line281-282: References are missing.

20) Line 304-306, the sentence "Cnpy1 has previously been studied" requires citation of relevant reference.

21) In line 349-350, the sentence "the basal VSNs of the Cnpy1 KO are nonfunctional" is not correct because Fig. 6 shows that some pS6-positive basal cells (notched arrowheads) are observed in Cnpy1 KO.

22) References described in line 504 and 546 are now published in eLife and genesis, respectively.

23) The misspellings I found are listed below. Please verify.
In line 12, "Gao" should be "Gαi2".
In line 13, "Gai2" should be "Gαo".
In line 42, "Riviere et al., 2009" should be "Rivière et al., 2009".
In line 114, "Gai2" should be "Gαi2".
In line 117, "Gao" should be "Gαo".
In line 143, "AP-2e" should be "AP-2ε".
In line 145, "(Fig. F)" should be "(Fig. 4F)".
In line 149, "(Fig. 4C-I)" should be "(Fig. 4G-I)".
In line 150, "(Gg8)" should be "(Gγ8 KO)".
Line 183, "Hspa5, was used to highlight the ER" A comma in this sentence should be removed.

© 2026. Published by The Company of Biologists under the terms of the Creative Commons Attribution License (https://creativecommons.org/licenses/by/4.0/).

In line 227, "V2R-VSs" should be"V2R VSNs".
In line 248, "Calr-4" should be "Calr4".
In line 308, "FGFr1" should be "Fgfr1".
In line 363, "Cal4" should be "Calr4".
In line 366, "AP-2e" should be "AP-2ε".
In line 501, "Prols, F." should be "Pröls, F."
In line 509, "2025.2010.2006.680635" should be "2025.10.06.680635".
In line 588, "Riviere, S." should be "Rivière, S."
In line 596, "Duffie, R." should be "Duffié, R."
In line 599, "e3822" should be "e22".
In line 614, "Nolle, A." should be "Nölle, A."
In line 678, "Cnpy1 KO at P2" may be "Cnpy1 KO at P21".
In line 714, "PS6" should be "pS6".
In line 715, "calculated BY" should be "calculated by".

First revision

Author response to reviewers' comments

We thank the reviewers for their prompt and thoughtful comments, which have helped us substantially strengthen the manuscript. In this revised version, we have addressed all major and minor concerns through extensive revisions, including new experiments across multiple postnatal stages, expanded single-cell transcriptomic analyses, improved quantification, additional immunohistochemical data, and the inclusion of new main and supplementary figures. These additions significantly enhance the robustness of our conclusions and clarify key aspects of Cnpy1 function in basal VSN biology.

During the revision process, a related study by Devakinandan et al. was published. Although both studies examine a similar knockout model, they reach markedly different conclusions. We have carefully addressed these discrepancies by incorporating additional analyses and explicitly discussing how differences in methodological approaches, particularly bulk versus single-cell strategies and protein-level analyses, can lead to divergent interpretations. Thanks to the reviewers' request, we have now identified a specific effect of Cnpy1 LOF on family-C receptor V2R expression.

Along with the contrasting findings in recent literature, our work contributes to a timely and important scientific discussion in the field. We therefore hope that the reviewers will support the publication of our extensively revised work.

Response to Reviewers:

Reviewer 1: The manuscript by Nicholas Mathias et al. examines the anatomical organization of the vomeronasal systems in a Cnpy1 KO mouse during postnatal stages. The authors report progressive degeneration and loss of basal VNO sensory neurons, as well as disrupted connectivity to the AOB, by using mainly immunolabeling methods. With some exceptions, the conclusions are generally well supported by the data. The manuscript can be improved with some revisions as suggested below.

Fig1: UMAP representation of VNO cells do not contain non-neuronal cell types such as sustentacular, glial, and other cell types. Please, indicate whether datasets were filtered for neuronal and stem cell lineages only in the Results and methods sections.

We generated a new supplementary figure 1, including UMAPs and heatmaps showing enriched genes in neuronal and non-neuronal clusters.

© 2026. Published by The Company of Biologists under the terms of the Creative Commons Attribution License (https://creativecommons.org/licenses/by/4.0/).

Transcriptomic analysis: no data regarding specific V2R expression (apart from Vmn2r2) is reported. In addition, the V2R receptor expression section (fig 7) generalizes about expression levels and fluorescence signal, when this can be only applied to the 1-2 receptors detected by the V2R2 antibody, which incidentally are expressed by most basal VSNs. Are there any other V2Rs among the DE genes? is cell reduction affecting a particular V2R-neuron subpopulation other than V2R2 (based on the RNAseq data)? what about H2-Mv genes and b2m-globulin, calretinin and other reported chaperones?

We have now added more data to respond to this point and analyzed the transcriptomic data of the V2R of families A-D, we arrived at new exciting conclusions (new Figure 7). While genes of family-C are significantly upregulated, we see no significant changes, regardless of the decrease in cell numbers in the expression of genes belonging to family A, B, D. Moreover, dot plot analysis points to consistent expression of genes belonging to different families across the whole VSN populations of WT and KO. Our single-cell data contrasts the conclusions of Devakinandar et al. as we see no decreases in V2R mRNA levels across families A,B,D, nor selective cell loss. Though mRNA shows increases or no changes, we still see differences in protein expression and detectability in the lumen.

We do not believe there is a major difference between our protein expression analysis and that of Devakinandan et al (see new figure 8, 9 and supplementary material). Their images also appear to show reduced V2R signal in the lumen, although this was not quantified in their study. In our material, we observe a clear reduction in immunoreactivity, with little to no detectable signal for several V2Rs at the level of the microvilli, a region enriched in Gao that we used for masking and quantification.

To further address the discrepancies between our study and that of Devakinandan et al., we performed additional immunostaining and quantification using multiple anti-V2R antibodies (FIGURE). These analyses reveal that the effects of Cnpy1 loss-of-function on V2R detectability vary by receptor and differ between somatic and luminal compartments. We also examined villar markers and provided additional evidence that the overall cytoarchitecture is comparable across genotypes.

Chemo-sensory activity analysis (Fig. 6): the pS6 immunolabeling is not very convincing, particularly in WT samples which show too many positive cells. It seems to me that background signal is too high. To have an idea of the basal level of pS6 activity, it is necessary to include a non-stimulated control. In addition, monitoring of c-Fos activity in the AOB of the same animals would strongly support the conclusion.

After the reviewers' comments, we purchased and tested multiple different anti-Ps6 antibodies. We made new experiments and added new data for, male-to-female, and female-to-male and male-to-male exposures at P21. We believe that the experiments are now much cleaner (new Figure 6). All conditions indicate a significant reduction in activation in basal VSNs we also observed increases in meis2-V1R activation in response female to male in the KO. This is somewhat in line with what was reported by Devakinandar and coworkers, though they do not specify how they merged male-to-female and female-to-male data into a single graph.

Fig. 8: The authors suggest that V2Rs do not reach lumen of the VNO, probably because defective trafficking and expression to the plasma membrane in the VSN dendrites/microvilli. To better support this conclusion, please show evidence reporting intact dendrite/microvillar integrity of basal VSNs in the KO mouse.

The KO show Gao staining in the lumen, which is enriched inn the villi. However, to better address this point, we now include AP-2eCre-A14-traced Cnpy1KOs and controls. We performed multiple labeling of the villi (new Figure 9). These data show that the overall cell architecture is largely similar between the KO and control groups after staining for villin, Tmem16a, and b2M. So, our data indicate that knobs and villi are present in the KO. Our new quantification confirmed a dramatic reduction in immunodetectable V2Rs in the lumen. However, in response to Devakinandar et al., we now show high-magnification, super-resolution images demonstrating that some cells

© 2026. Published by The Company of Biologists under the terms of the Creative Commons Attribution License (https://creativecommons.org/licenses/by/4.0/).

still show low V2R reactivity in the villi (new fig 8 P-Q). So the V2Rs are not completely absent in the villi but drastically reduced.

Fig. 10: the observed increase in glomerular size is somehow puzzling. Are glomeruli indeed larger? Or the observation is rather a decrease in small glomeruli?

Thank you for requesting further clarification. We conducted new quantifications on both P21 and P30 animals and binned the glomeruli by size ranges (see New Fig 13 and S.7). Our analysis shows that as cell loss increases, neurons decrease across glomeruli of all sizes; the loss appears to be distributed, and no particular glomeruli size stands out for higher loss. Consistent with transcriptomic data, we do not observe a clear bias in the loss of VSNs forming glomeruli of specific sizes. However, despite the overall reduction in glomeruli, the proportion of larger or possibly less defined glomeruli is higher, which, together with cell loss, probably reflects dysregulation of adhesion molecules (see Fig 12, 13, and S7).

Fig. 11C: Using immunofluorescence density signal as a proxy for Cnpy1 expression is not a reliable method, and can be subject to bias derived from technical variability. Other quantitative, more precise methods such as qPCR would be more appropriate. Otherwise, the conclusion that "we observe reduced Cnpy1 expression" (line 367) should be removed. Alternatively, this piece of data is only tangential to the study and can be skipped, as it does not provide much new information.

Following the reviewer's suggestion, and to stay within the word limit for Development, we removed these data sets, which, as the reviewer points out, are tangential to the story. We will expand on the Ap-2e control of ER proteins in an upcoming follow-up article that we are currently preparing based on new Sc-RA seq from Ap-2eplilon KOs. We also removed the cartoon (e.g., Fig.13) to keep the message more focused.

Discussion: the authors emphasize the lack of studies involving Cnpy1 in mammals. It would be advisable to provide some context information about canopy expression in other cell types outside of the VNO, and potential interaction with other receptors different from V2Rs (perhaps other class C GPCRs) and their trafficking to the plasma membrane. This will provide key information about the specificity of Cnpy1 expression and function in the VNO.

We address this in Supplementary Figure 2 and in the discussion. Although we observed transient Cnpy1 mRNA expression in the hindbrain and MOE, we could never detect the protein, so we cannot speculate much. However, we expanded the discussion of the results as a general concept about ER diversity.

Minor:

- Abstract (and whole text): symbols of specific proteins (alpha, epsilon, gamma…) are sometimes typed in the Latin forms. Please, stick to one specific alphabet (preferably Greek) for consistency.

- Line 60: remove "CUT&RUN" from the text. Done

- line 124: no need to report that the Cnpy1 knockout mouse is "unpublished". Done

- line 191: specify which "lumen" (VNO lumen)

-line 338: "… that ER stress physiologically increases…" better indicate ER stress-related genes

-Fig. 6C: graph data should be expressed in real units (VSNs/section or similar)

We revised all these

Reviewer 2: SUMMARY OF THE ADVANCE MADE IN THIS PAPER AND ITS POTENTIAL SIGNIFICANCE TO THE FIELD

© 2026. Published by The Company of Biologists under the terms of the Creative Commons Attribution License (https://creativecommons.org/licenses/by/4.0/).

This manuscript by Mathias et al. provides a thorough investigation into the role of the ER protein Canopy1 (Cnpy1) in the development and maintenance of basal vomeronasal sensory neurons (VSNs). The authors convincingly demonstrate that Cnpy1 expression is restricted to differentiating/maturing basal VSNs and that its ablation results in progressive loss of these neurons postnatally, accompanied by reduced V2R expression and mislocalization, ultimately impairing neuronal activation in response to urine chemosignals.

The manuscript is generally well written, and the data are compelling. It is worth noting that the authors acknowledge a recent study by Devakinandan et al. (bioRxiv, 2025) that also addresses Cnpy1 function in basal VSNs. While both studies confirm a role for Cnpy1, they provide complementary insights. Mathias et al. uniquely contribute by characterizing cell surface and axon guidance receptors in basal VSNs, identifying glomerular defects in Cnpy1 mutants, and demonstrating that Cnpy1 expression is reduced in AP2E mutants, implicating AP2E in transcriptional regulation of Cnpy1.

Overall, this is a strong manuscript that makes a valuable contribution to the field. The findings are significant and will be of great interest to developmental biologists studying sensory neuron differentiation and fate specification.

Specific comments:

1. There are two important discrepancies between the results presented by the authors and Devakinandan et al. (cell death and V2R localization in Cnpy1 mutants), which should be explicitly discussed, and for which potential explanations be offered to help readers reconcile the differences.

We note that the statistical approach used by Devakinandan et al. is not described in sufficient detail to fully assess how significance was determined. Based on the presentation of the data, several graphs which were derived from 5 animals include 50-100 points per condition, which may suggest that measurements from individual sections were analyzed as individual data points rather than averaging data from each VNO or animal. We believe that they only reached significant values by overpowering the P value. In our study, data from each animal was considered a replicate, and measurements obtained from multiple sections were averaged to generate a single value.

Importantly, even assuming that the reported statistical significance by Devakinandan et al is valid, the magnitude of the differences appears modest and does not explain the extent of neuronal loss observed.

Regarding the V2R's detectability, we do not believe there are real differences between our and those by Devakinandan et al. ; after performing new staining and quantifications with other V2R antibodies, we stand by our claim: immunoreactivity in the lumen of the KO is severely reduced. High-magnification images show residual detectability of V2Rs in some neurons, so we adjusted the wording accordingly.

2. As Devakinandan et al. reported increased apoptosis in the VNO of Cnpy1 mutants at earlier postnatal stages than the one assessed by the authors, they should perform cleaved caspase-3 (CC3) staining at earlier time points to determine whether transient apoptotic events were missed in their analysis.

As noted above, differences in statistical outcomes between our study and that of Devakinandan et al. may reflect differences in how data from individual sections were used in the statistical analyses. Based on our interpretation of their figures and the lack of description of the statistical approach, it appears that counts from individual sections were used as independent data points rather than averaged per VNO or animal.

Anyway, to facilitate a direct comparison, we have now included new cleaved caspase-3 quantification at P14, a similar developmental stage to that analyzed by Devakinandan et al. Using

© 2026. Published by The Company of Biologists under the terms of the Creative Commons Attribution License (https://creativecommons.org/licenses/by/4.0/).

this approach, we still do not find a significant increase in apoptosis. We now report both total CC3 counts and CC3-positive cells within V2R-expressing neurons (New Figure 4)

3. The authors propose that reduced Kirrel2 expression contributes to posterior AOB glomerular defects in Cnpy1 mutants. Since glomerular formation occurs during early postnatal development, it would be important to assess Kirrel2 expression in basal VSNs at earlier stages to strengthen this conclusion.

We added violin plots from single-cell data in P21 animals and performed RNA Scope at P21 (new Fig. 12), along with the AOB images at P21 (Fig. 13) and P30 (S. 7). These data confirm the loss of Kirrel2 expression and a significant increase in Kirrel3 in the basal VSNs, supporting what is expected given the lack of VSN functionality in animals with a still relatively normal VNO (P21 only -11% decrease in V2R VSNs).

Minor comments:

1. The increased Vmn2R2 mRNA expression in Cnpy1 KO animals is shown in Figure 7, and mentioned in the discussion, but should also be addressed in the text of the results section.

We extensively revised this part, added new data, made a new Figure 7, and revised the text.

2. As loss of Cnpy1 leads to reduced Kirrel2 expression, but not Kirrel3, in basal VSNs, the authors should consider referencing the paper by Brignall et al. in 2018, which reports similar glomerular defects to Cnpy1 when Kirrel2 is ablated from VSNs.

The reference to Price et al was added.

3. On Line 115, in the sentence: "However, its mRNA expression rapidly declines in differentiating/maturing (Meis2+; Gai2/Gnai2) V1R VSNs while remaining active and highly correlated with the expression of genes belonging to the V2R basal lineage (Fig. 1A, 2A).

It is unclear what is "remaining active". Do they mean "while remaining expressed"?

4.Line 236: "Quantification of glomeruli number and size showed an overall reduction of the posterior AOB (Fig. 10G), which, like for other mutants, reflects a reduction of basal VSNs." It is unclear which mutants are being referred to in this sentence.

5.Line 293 " Cnpy1 is the among the most highly expressed genes " should be "is among the..."

6.In Figure 4, it is unclear how the average number of VSNs/section can be lower in VNOS from P60 mice than VNOs from P21 mice considering that the VNO should be larger in older mice.

7.P values are missing on graphs in Figure 10

8.For Figure 8, p value on graph is $p=0.0001$, while it is reported as $p<0.0001$ in the figure legend.

9.The Figure legends should be reviewed to ensure consistency of the information provided across the legends.

We made all minor correction

Reviewer 3: SUMMARY OF THE ADVANCE MADE IN THIS PAPER AND ITS POTENTIAL SIGNIFICANCE TO THE FIELD

In this manuscript, Mathias et al. investigate the functional role of the ER-associated protein Canopy 1 (Cnpy1), which is one of the genes differentially expressed between V1R- and V2R-expressing VSNs. In the V2R-expressing VSN lineage, Cnpy1 expression is maintained from globose basal progenitors through to mature VSNs, whereas its expression rapidly declines during differentiation of the V1R-expressing VSN lineage.

© 2026. Published by The Company of Biologists under the terms of the Creative Commons Attribution License (https://creativecommons.org/licenses/by/4.0/).

The authors performed histological and transcriptomic analyses of Cnpy1 knockout (KO) mice and demonstrate that loss of Cnpy1 results in a reduction in the number of V2R-VSNs, altered expression of ER stress-responsive genes and axon guidance molecules, and disorganization of the posterior accessory olfactory bulb (AOB). Upon exposure to male urine, the number of activated VSNs is significantly reduced specifically in V2R-VSNs of Cnpy1 KO males. Furthermore, immunohistochemical analysis of V2R2, an atypical V2R broadly expressed in V2R-VSNs, shows reduced V2R2 immunoreactivity in cell bodies and complete loss of signal in the dendritic lumen of the Cnpy1 KO vomeronasal organ. These results suggest that impaired transport of V2R2 protein to the dendritic lumen leads to reduced neuronal activity in V2R-VSNs.

Overall, this study demonstrates an important role for Cnpy1 in V2R processing and transport, which is essential for proper V2R-VSN function. The manuscript is technically sound and presents potentially important findings, with several conclusions supported by convincing data. However, I would like the authors to address several major issues, which are outlined below.

SUGGESTIONS TO AUTHORS

Major points

1) In this study, the authors performed immunohistochemical analyses of V2R2, a family C V2R. V2R2 is an atypical V2R that is broadly expressed in the basal layer of vomeronasal sensory neurons (VSNs) and is co-expressed with typical V2Rs (family ABD V2Rs). Previous work has shown that loss of Vmn2r1, a member of the family C V2Rs, leads to a reduction in the basal V2R-expressing VSN population (Akiyoshi et al., 2018; doi:10.1111/ejn.13875); however, the functional contribution of family C V2Rs to pheromone-evoked VSN responses remains unclear. In this context, it is essential that the authors also examine the expression of typical V2R proteins. Such analyses would help clarify the basis for the reduced responses of basal VSNs observed in the Cnpy1 knockout (Fig. 6) and would substantially strengthen the reliability of the conclusions and the overall significance of the manuscript.

In addition, the manuscript describes the results of the V2R2 analysis as though they are representative of typical V2Rs, which is not accurate. Conclusions based on V2R2 should be presented with appropriate caution and clearly framed as findings specific to V2R2 rather than generalized to typical V2Rs.

Based on this reviewer's comment, we extensively revised the whole manuscript.

2) Line 147-152, Fig. 4: The mechanism underlying the reduction in basal vomeronasal sensory neurons (VSNs) in the Cnpy1 knockout remains unclear. Although the authors quantified apoptotic cells across the entire vomeronasal epithelium, the reported neuronal loss appears to be restricted to the basal region. Therefore, apoptotic cell counts should be specifically quantified within the basal layer.

As discussed above, we added new Caspase3 at P14, and counted cells in the basal layer, see new Figure 4.

Moreover, the authors state that a "progressive neurodegenerative phenotype begins prior to maturation of the neuron" (Lines 162-163). To support this claim, it would be more informative to quantify active caspase-3-positive cells separately in the marginal zone and in the basal mature VSN region, as schematically illustrated in Fig. 12.

In addition, because the observed reduction in VSN numbers could also result from impaired or aberrant cell proliferation, the authors should quantify proliferative cells in the Cnpy1 knockout to more fully address the underlying cellular mechanisms.

Ki67 and cell counts at P14 showed no differences in proliferation or in the initial cell number (Fig.4)

© 2026. Published by The Company of Biologists under the terms of the Creative Commons Attribution License (https://creativecommons.org/licenses/by/4.0/).

3) It is difficult to clearly identify pS6-positive cells in the presented images. Several cells indicated by arrowheads exhibit signal intensities comparable to those observed in the sustentacular cell layer and are therefore likely to represent background staining rather than true pS6 signals. The staining protocol and/or image acquisition conditions should be optimized to obtain more reliable and quantitatively robust data.

In addition, because the number of V2R-expressing VSNs is reduced in the Cnpy1 knockout, pS6-positive cell counts should be presented in a normalized manner (e.g., relative to the total number of basal or apical VSNs), rather than as absolute values.Furthermore, according to Silvotti et al. (2018; reference cited at line 602), pS6 signals are more prominently detected in mice stimulated with pheromones secreted by the opposite sex. Therefore, I recommend assessing VNO activation in female mice exposed to male urine.

We purchased new anti-ps6 antibodies, obtained clean results, added new conditions, and consistently observed reduced activation in the Cnpy1KOs. New Fig. 6.

4) Line 212-214: The authors report that endoplasmic reticulum (ER) responses are increased in mature VSNs; however, Fig. 9 appears to show altered expression of signaling molecules associated with ER responses rather than direct evidence of increased ER stress. It is unclear whether the expression changes of all genes described are strictly dependent on activation of the ER stress response.

To more directly support the claim of enhanced ER stress, I suggest assessing the extent to which canonical ER stress signaling pathways are activated. For example, Shayya et al. (2022; reference cited at line 596) quantified the unfolded protein response (UPR) by measuring changes in Atf5 mRNA translation regulated by PERK-mediated eIF2α phosphorylation. Similarly, Dey and Matsunami (reference cited at line 510) evaluated UPR activation based on Xbp1 mRNA splicing mediated by IRE1 activation. Incorporating comparable analyses would substantially strengthen the interpretation of the data.

We believe that gene expression analysis from bulk lysed samples of the entire VNE, as performed by Devakinandan, is not informative due to the asynchrony of cells and molecular heterogeneity across VSNs. They used mRNA from whole VNOs from multiple animals..

Using single cells, we observe that at the transcriptomic level, around 15-25% of cells show increased ER stress response gene markers in Cnpy1KOs (See fig. 10D). We also indicate that, physiologically, the V2RS have higher ER stress gene expression.

To obtain quantifiable data, we used antibodies that specifically recognize XBP1s, the spliced protein form of XBP1 activated by ATF6 in response to increased stress. This staining clearly demonstrated 1) that V2R VSNs have higher stress levels than V1R VSNs and 2) that there is a significant increase in ER stress gene expression confined to the V2R VSNs of Cnpy1 KOs, which we believe corroborates our claims.

5) Prince et al. (2013; reference cited at line 582) reported that the expression levels of Kirrel2 and Kirrel3 are activity dependent and suggested that the expression of these cell adhesion molecules in VSNs correlates with the specific V1R or V2R expressed. Given that Fig. 10 shows Kirrel3-expressing basal VSNs projecting to the posterior accessory olfactory bulb (AOB) in Cnpy1 knockout mice, it is possible that V2R-expressing neurons with high Kirrel3 expression are preferentially maintained in this genotype.

This is possible; however, our data at P21, where cell loss is still modest (-11%) and we do not see Kirrell2 in V2Rs, does not point to loss of specific neuronal types. Cnpy1 KO loose VSNs and glomeruli of different sizes in a similar proportion. We believe that the new Figs. 12, 13, and S.7 address these concerns.

© 2026. Published by The Company of Biologists under the terms of the Creative Commons Attribution License (https://creativecommons.org/licenses/by/4.0/).

In addition, family ABD V2Rs can be subdivided into C1-type V2Rs, which are co-expressed with Vmn1r1, and C2-type V2Rs, which are co-expressed with Vmn2r2-7 (Silvotti et al., 2011; doi:10.1371/journal.pone.0024462). Loss of Vmn2r1 results in a significant reduction in the number of C1-type V2R-expressing VSNs (Akiyoshi et al., 2018; doi:10.1111/ejn.13875). Considering the reported impairment of V2R2 protein expression, it is therefore expected that specific basal VSN subpopulations are selectively reduced in the Cnpy1 knockout.

Because single-cell transcriptomic analysis of Cnpy1 knockout mice is presented in Fig. 5, the expression patterns of individual V1R and V2R genes could be further interrogated. It would be informative if the authors could identify which receptor-expressing VSN populations persist in the Cnpy1 knockout and discuss potential mechanisms underlying the selective survival of these V2R-expressing neurons.

We do not have enough cells in our data to perform specific analysis of changes in expression of individual VRs. However, our data grouped by VR families show increases in mRNA for family C and no or little effect on the mRNA levels of the other V2R families (new Fig. 7).

6) Fig. 13: The authors should more clearly articulate the functional role of Cnpy1 as revealed by this study. One plausible interpretation is that Cnpy1, together with other ER-associated proteins, is induced downstream of AP-2ε-regulated effector gene activation, and that these ER proteins are required for proper processing and trafficking of V2R2 proteins (and potentially typical V2Rs, which should be examined by immunohistochemistry as noted in Comment #1). Impaired processing of these receptors may, in turn, influence the expression of cell adhesion molecules, thereby affecting the organization of glomeruli in the accessory olfactory bulb (AOB).

Thank you, we tried to deliver this message better in the discussion.

The text has been extensively revised, and we believe we have corrected most of the minor points. Thanks for such a thorough review.

Minor points

7) Line 25-26: In the abstract, the authors state "distinct neuronal types depend on unique ER protein repertoires to maintain proper proteostasis". I think this statement holds true only if the same phenomena are obtained in V1R-expressingVSNs. I suggest rewording this sentence.

8) Line 41-42: Four references about FPRs are cited, and references describe V1Rs and V2Rs should be also cited here.

9) Fig 2A: Do red and blue colors indicate the correlation coefficients, and what do the stripes indicate?

10) Fig 4A, B, D, E: Please mention arrowheads indicate in the legend.

11) Related Fig 4C, 4F: Please describe the quantification methods in detail, thickness of section, numbers of quantified sections, and sex of animals is missing in the Materials and Methods.

12) Line 156: "red and blue" is different from Fig 5A legend.

13) Family C V2R includes Vmn2r1-7, and which V2R does the anti-V2R2 antibody detect? In Fig. 7D, is the expression of all family C V2R mRNA increased in Cnpy1 KO?

14) Arrowheads in Fig.8 are referred to as "arrows" in the legend and line 195.

15) Line 205-206: "we observed markedly reduced immunoreactivity for V2R receptors, consistent with defects in receptor processing and trafficking." In this sentence, "suggesting" is more appropriate than "consistent with ".

16) In Fig. 10C and C', "Cnpy1-/-" should be "Cnpy1 KO".

© 2026. Published by The Company of Biologists under the terms of the Creative Commons Attribution License (https://creativecommons.org/licenses/by/4.0/).

17) In line 264-265, the name of strain "Neurogenin1-CreERt2" is referred to as "Neurog1-cre/ERT2" in the Materials and Methods, "Ngn1-Cre" in Fig. 12, and "Ngn1-CreERT2" in the Fig. 12 legend; consistency is required.

18) Line 217: Atf5 should be Atf6? In addition, Expression levels of Atf5 decrease in Cnpy1 KO, opposite to other genes. Please mention Atf5.

19) Line281-282: References are missing.

20) Line 304-306, the sentence "Cnpy1 has previously been studied" requires citation of relevant reference.

21) In line 349-350, the sentence "the basal VSNs of the Cnpy1 KO are nonfunctional" is not correct because Fig. 6 shows that some pS6-positive basal cells (notched arrowheads) are observed in Cnpy1 KO.

22) References described in line 504 and 546 are now published in eLife and genesis, respectively.

23) The misspellings I found are listed below. Please verify.
In line 12, "Gao" should be "Gαi2".
In line 13, "Gai2" should be "Gαo".
In line 42, "Riviere et al., 2009" should be "Rivière et al., 2009".
In line 114, "Gai2" should be "Gαi2".
In line 117, "Gao" should be "Gαo".
In line 143, "AP-2e" should be "AP-2ε".
In line 145, "(Fig. F)" should be "(Fig. 4F)".
In line 149, "(Fig. 4C-I)" should be "(Fig. 4G-I)".
In line 150, "(Gg8)" should be "(Gγ8 KO)".
Line 183, "Hspa5, was used to highlight the ER" A comma in this sentence should be removed.
In line 227, "V2R-VSs" should be"V2R VSNs".
In line 248, "Calr-4" should be "Calr4".
In line 308, "FGFr1" should be "Fgfr1".
In line 363, "Cal4" should be "Calr4".
In line 366, "AP-2e" should be "AP-2ε".
In line 501, "Prols, F." should be "Pröls, F."
In line 509, "2025.2010.2006.680635" should be "2025.10.06.680635".
In line 588, "Riviere, S." should be "Rivière, S."
In line 596, "Duffie, R." should be "Duffié, R."
In line 599, "e3822" should be "e22".
In line 614, "Nolle, A." should be "Nölle, A."
In line 678, "Cnpy1 KO at P2" may be "Cnpy1 KO at P21".
In line 714, "PS6" should be "pS6".
In line 715, "calculated BY" should be "calculated by".

Second decision letter

MS ID#: dev.205386R1

MS TITLE: Canopy1/Cnpy1 is required for proper V2R processing and transport; its loss impairs the function and circuit organization of basal/V2R vomeronasal sensory neurons.

AUTHORS: Nicholas Mathias; Nikki Dolphin; Noah M. LeFever; Enrico Amato; Carlsy Ybanez; Anthony Ruquet; Paolo E. Forni

Dear Dr Forni,

© 2026. Published by The Company of Biologists under the terms of the Creative Commons Attribution License (https://creativecommons.org/licenses/by/4.0/).

I have now received all the referees reports on the above manuscript, and have reached a decision. The referees' comments are appended below.

The overall evaluation is positive and we would like to publish a revised manuscript in Development, provided that the referees' comments can be satisfactorily addressed. Reviewer 3 has some minor issues, please attend to these in your revised manuscript and detail them in your point-by-point response. If you do not agree with any of their criticisms or suggestions explain clearly why this is so.

Reviewer 1

The authors have addressed all of my initial concerns. The new manuscript version is highly improved.

Reviewer 2

SUMMARY OF THE ADVANCE MADE IN THIS PAPER AND ITS POTENTIAL SIGNIFICANCE TO THE FIELD

This manuscript by Mathias et al. provides a thorough investigation into the role of the ER protein Canopy1 (Cnpy1) in the development and maintenance of basal vomeronasal sensory neurons (VSNs). Overall, this is a strong manuscript that makes a valuable contribution to the field. The findings are significant and will be of great interest to developmental biologists studying sensory neuron differentiation and fate specification. The authors have addressed all my previous concerns through the addition of experimental data, as well as by modifying the manuscript text. As such, I recommend publication of the manuscript in Development.

Reviewer 3

SUMMARY OF THE ADVANCE MADE IN THIS PAPER AND ITS POTENTIAL SIGNIFICANCE TO THE FIELD

I have reviewed the revised manuscript and carefully evaluated the authors' responses to my previous comments. I appreciate the considerable effort the authors have made to improve the manuscript. Most of my concerns have been adequately addressed. However, several minor issues remain or have newly arisen in the revised version.

SUGGESTIONS TO AUTHORS

Minor Points (The numbering corresponds to that used in my previous review comments.)
8) Line 41-42 (Lines 28-30 in the revised manuscript): Four references related to FPRs are cited; however, references describing V1Rs and V2Rs should also be included here.
Please revise the manuscript accordingly.

10) Figure 4A, B, D, and E: Please clarify the meaning of the arrowheads in the figure legend. Accordingly, please revise the legend for Figure 4.
In addition, in Figure 3, arrowheads indicate basal VSNs, whereas notched arrowheads indicate apical VSNs. The use and meaning of these symbols should be kept consistent throughout the manuscript.

Newly Arisen Points 1) In Figure 7, "V2R2 (Family A, B, C, D)" should be corrected to "V2R (Family A, B, C, D)."

2) In addition to the above point, the authors describe the classification of V2R genes into Families A-D in the main text (Lines 173-174), but Family E is not mentioned. Please include a description of Family E and cite the appropriate references.

© 2026. Published by The Company of Biologists under the terms of the Creative Commons Attribution License (https://creativecommons.org/licenses/by/4.0/).

3) In Figure 7C, please include data for Vmn2r4 and Vmn2r5, as Vmn2r1-Vmn2r7 belong to Family C V2Rs. Alternatively, please explain in the main text why these genes were not included in the analysis.

4) The results presented in Fig. 8C-C'''' are inconsistent with the corresponding descriptions in the main text (lines 192-195). In addition, the legend for Fig. S7 does not match either the figure or the text. Furthermore, the descriptions associated with Fig. 8D-O, its legend, and the Discussion section (lines 359-361) are also inconsistent with the presented results. The authors should carefully verify that all figure panels, legends, and descriptions in the text are fully consistent with the data shown and revise all discrepancies accordingly.

5) In Fig. 13A, the "Hand tracing" label is displaced outside the image and should be repositioned appropriately.

6) The following citation and textual errors also require correction.
Possible citation errors:
"S.2" in line 198
"Fig. 8A', B''" in line 200
"Fig. 10G" in line 228
"Fig. S7" in line 261
"Fig. S7 G-H" in line 263 Possible textual errors:
"Gαo KO" in line 159 should be changed to "Gαo cKO"
"Ai4" in line 276 should be changed to "Ai14"

Second revision

Author response to reviewers' comments

We sincerely thank the reviewer for the care and time spent on reviewing this paper and finding all of these imperfections/mistakes. This was a very generous contribution to our work.

Reviewer 3: SUMMARY OF THE ADVANCE MADE IN THIS PAPER AND ITS POTENTIAL SIGNIFICANCE TO THE FIELD

I have reviewed the revised manuscript and carefully evaluated the authors' responses to my previous comments. I appreciate the considerable effort the authors have made to improve the manuscript. Most of my concerns have been adequately addressed. However, several minor issues remain or have newly arisen in the revised version.

SUGGESTIONS TO AUTHORS

Minor Points (The numbering corresponds to that used in my previous review comments.)

8) Line 41-42 (Lines 28-30 in the revised manuscript): Four references related to FPRs are cited; however, references describing V1Rs and V2Rs should also be included here.

Please revise the manuscript accordingly.

Added references to (Francia et al., 2014; Herrada and Dulac, 1997; Matsunami and Buck, 1997; Ryba and Tirindelli, 1997; Silvotti et al., 2007)

10) Figure 4A, B, D, and E: Please clarify the meaning of the arrowheads in the figure legend.

Accordingly, please revise the legend for Figure 4.

© 2026. Published by The Company of Biologists under the terms of the Creative Commons Attribution License (https://creativecommons.org/licenses/by/4.0/).

Added "Arrowheads and Notched Arrowheads indicate the V1R+ and V2R+ sensory epithelia respectively"

In addition, in Figure 3, arrowheads indicate basal VSNs, whereas notched arrowheads indicate apical VSNs. The use and meaning of these symbols should be kept consistent throughout the manuscript.

Notched Arrows made consistent with basal VSN Layer.

Newly Arisen Points

1) In Figure 7, "V2R2 (Family A, B, C, D)" should be corrected to "V2R (Family A, B, C, D)."

Corrected.

We also added to the legend of the figure "Module Scores" to specify the used Expression metric.

2) In addition to the above point, the authors describe the classification of V2R genes into Families A-D in the main text (Lines 173-174), but Family E is not mentioned. Please include a description of Family E and cite the appropriate references.

We added reference Francia et al 2014

3) In Figure 7C, please include data for Vmn2r4 and Vmn2r5, as Vmn2r1-Vmn2r7 belong to Family C V2Rs. Alternatively, please explain in the main text why these genes were not included in the analysis.

Vmn2R4 and Vmn2R5 have relatively few cells and were excluded from analysis, we now specified this in the text

4) The results presented in Fig. 8C-C"" are inconsistent with the corresponding descriptions in the main text (lines 192-195). Fixed, we meant to refer supplementary figure S3: creation of Imaris Masks

In addition, the legend for Fig. S7 does not match either the figure or the text. We fixed, that was an old numbering referring to what is now Fig. S8

Furthermore, the descriptions associated with Fig. 8D-O, its legend, and the Discussion section (lines 359-361) are also inconsistent with the presented results. The authors should carefully verify that all figure panels, legends, and descriptions in the text are fully consistent with the data shown and revise all discrepancies accordingly.

We made some edits where we think there may have been ambiguity. For the remainder of the text, the results are coherent with Figure 8; however, we made minor edits to the legend for clarity.

5) In Fig. 13A, the "Hand tracing" label is displaced outside the image and should be repositioned appropriately

The alignment was adjusted.

6) The following citation and textual errors also require correction.

Possible citation errors:

"S.2" in line 198, S.2 mistakenly referred to in line 190, fixed to S3

© 2026. Published by The Company of Biologists under the terms of the Creative Commons Attribution License (https://creativecommons.org/licenses/by/4.0/).

"Fig. 8A', B'" in line 200, changed to Fig. 8D-S

"Fig. 10G" in line 228, this is correct reference

"Fig. S7" in line 261, changed to Fig. S8

"Fig. S7 G-H" in line 263, changed to Fig. S8

Possible textual errors:

"Gαo KO" in line 159 should be changed to "Gαo cKO", changed to "Gao cKO" to reflect the OMP-Cre mediated Gao-flx recombination

"Ai4" in line 276 should be changed to "Ai14", fixed

Third decision letter

MS ID#: dev.205386R2

MS TITLE: Canopy1/Cnpy1 is required for proper V2R processing and transport; its loss impairs the function and circuit organization of basal/V2R vomeronasal sensory neurons.

AUTHORS: Nicholas Mathias; Nikki Dolphin; Noah M. LeFever; Enrico Amato; Carlsy Ybanez; Anthony Ruquet; Paolo E. Forni

Dear Dr Forni,

I am happy to tell you that your manuscript has been accepted for publication in Development, pending our standard publication integrity checks.

© 2026. Published by The Company of Biologists under the terms of the Creative Commons Attribution License (https://creativecommons.org/licenses/by/4.0/).
